# Genomic diversity and post-admixture adaptation in the Uyghurs

**DOI:** 10.1093/nsr/nwab124

**Published:** 2021-09-11

**Authors:** Yuwen Pan, Chao Zhang, Yan Lu, Zhilin Ning, Dongsheng Lu, Yang Gao, Xiaohan Zhao, Yajun Yang, Yaqun Guan, Dolikun Mamatyusupu, Shuhua Xu

**Affiliations:** Key Laboratory of Computational Biology, Shanghai Institute of Nutrition and Health, University of Chinese Academy of Sciences, Chinese Academy of Sciences, Shanghai 200031, China; Key Laboratory of Computational Biology, Shanghai Institute of Nutrition and Health, University of Chinese Academy of Sciences, Chinese Academy of Sciences, Shanghai 200031, China; State Key Laboratory of Genetic Engineering and Ministry of Education (MOE) Key Laboratory of Contemporary Anthropology, Collaborative Innovation Center for Genetics and Development, School of Life Sciences, Fudan University, Shanghai 200438, China; Key Laboratory of Computational Biology, Shanghai Institute of Nutrition and Health, University of Chinese Academy of Sciences, Chinese Academy of Sciences, Shanghai 200031, China; Key Laboratory of Computational Biology, Shanghai Institute of Nutrition and Health, University of Chinese Academy of Sciences, Chinese Academy of Sciences, Shanghai 200031, China; Key Laboratory of Computational Biology, Shanghai Institute of Nutrition and Health, University of Chinese Academy of Sciences, Chinese Academy of Sciences, Shanghai 200031, China; School of Life Science and Technology, ShanghaiTech University, Shanghai 201210, China; Human Phenome Institute, Fudan University, Shanghai 201203, China; State Key Laboratory of Genetic Engineering and Ministry of Education (MOE) Key Laboratory of Contemporary Anthropology, Collaborative Innovation Center for Genetics and Development, School of Life Sciences, Fudan University, Shanghai 200438, China; Department of Biochemistry and Molecular Biology, Preclinical Medicine College, Xinjiang Medical University, Urumqi 830011, China; College of the Life Sciences and Technology, Xinjiang University, Urumqi 830046, China; Key Laboratory of Computational Biology, Shanghai Institute of Nutrition and Health, University of Chinese Academy of Sciences, Chinese Academy of Sciences, Shanghai 200031, China; State Key Laboratory of Genetic Engineering and Ministry of Education (MOE) Key Laboratory of Contemporary Anthropology, Collaborative Innovation Center for Genetics and Development, School of Life Sciences, Fudan University, Shanghai 200438, China; School of Life Science and Technology, ShanghaiTech University, Shanghai 201210, China; Human Phenome Institute, Fudan University, Shanghai 201203, China; Center for Excellence in Animal Evolution and Genetics, Chinese Academy of Sciences, Kunming 650223, China; Henan Institute of Medical and Pharmaceutical Sciences, Zhengzhou University, Zhengzhou 450052, China

**Keywords:** Uyghur, Eurasian, genetic admixture, genomic diversity, local adaptation

## Abstract

Population admixture results in genome-wide combinations of genetic variants derived from different ancestral populations of distinct ancestry, thus providing a unique opportunity for understanding the genetic determinants of phenotypic variation in humans. Here, we used whole-genome sequencing of 92 individuals with high coverage (30–60×) to systematically investigate genomic diversity in the Uyghurs living in Xinjiang, China (XJU), an admixed population of both European-like and East-Asian-like ancestry. The XJU population shows greater genetic diversity, especially a higher proportion of rare variants, compared with their ancestral source populations, corresponding to greater phenotypic diversity of XJU. Admixture-induced functional variants in *EDAR* were associated with the diversity of facial morphology in XJU. Interestingly, the interaction of functional variants between *SLC24A5* and *OCA2* likely influences the diversity of skin pigmentation. Notably, selection has seemingly been relaxed or canceled in several genes with significantly biased ancestry, such as *HERC2*–*OCA2*. Moreover, signatures of post-admixture adaptation in XJU were identified, including genes related to metabolism (e.g. *CYP2D6*), digestion (e.g. *COL11A1*), olfactory perception (e.g. *ANO2*) and immunity (e.g. *HLA*). Our results demonstrated population admixture as a driving force, locally or globally, in shaping human genetic and phenotypic diversity as well as in adaptive evolution.

## INTRODUCTION

Human migration leads to both population differentiation and genetic contact between long-isolated ethnic groups. The ancient trade route known as the Silk Road (∼2000 years before present, YBP), between China, Central Asia, India and Western Europe, was one of the crucial human migration routes, resulting in gene flow between originally isolated populations and shaping the genetic diversity of contemporary Eurasians. Present-day populations in Northwest China, or geographically Central Asia, such as Tajiks, Uyghurs and Uzbeks [1], exhibit genetic admixture of distinct ancestries of East Asian and European origin. For example, Xu *et al.* [2,3] described the genetic make-up of the Uyghurs as an admixture with approximately half East Asian-like and half European-like ancestry. Recently, Feng *et al.* [4] dissected the ancestry composition of the Uyghurs living in Xinjiang (XJU) on a finer scale and identified four major ancestral components, i.e. European (EUR, 25%–37%), South Asian (SAS, 12%–20%), Siberian (SIB, 15%–17%) and East Asian (EAS, 29%–47%). Accordingly, the study unveiled a more complex scenario of ancestral origin and admixture history in the XJU than previously assumed.

Genetic admixture increases genomic variation and creates novel genetic combinations as it brings together genomic segments from genetically disparate populations, thereby affording unprecedented opportunities to investigate the functional implications of natural variation across human genomes. Originally neutral genetic variants derived from ancestral populations may either maintain their neutral status or experience local adaptation post admixture in a new environment. Genetic components from divergent ancestries can also gain new functions via interaction. The admixture and subsequent local adaptations together drive the micro-evolution of admixed populations. Previous admixture mapping studies have identified putatively causative alleles and have successfully mapped disease loci associated with type 2 diabetes in Hispanics [5], hypertension in African Americans [6] and prostate cancer in African-American men [7]. Pre- and post-selection scanning for African Americans identified many selection-candidate genes associated with high-risk diseases, such as prostate cancer and hypertension, implying local adaptations of admixed populations in new environments [8]. A recent study also found evidence for admixture-enabled selection in Latin American populations, for example, the enhanced adaptive immune response [9].

XJU is a well-known admixed population that has experienced even more complex admixture events than many other populations, such as African Americans. However, it remains unclear how admixture has shaped genetic diversity and driven the local adaptation of XJU as well as other Eurasian admixed populations. Under the neutral model of evolution, it is expected that the genome-wide variation of XJU people could be well interpreted by their ancestral populations residing in western and eastern Eurasia. However, this scenario would be complicated by the multiple population admixture events spanning large spatial and temporal scales. The evolution of the genomic diversity and phenotypic diversity of the XJU was likely driven by gene flow from the surrounding populations and by adaptation to the local environment.

We collected 92 samples of XJU from nine prefectures (Kaxgar, Hotan, Kizilsu, Aksu, Bayingolin, Turpan, Changji, Ili and Bortala) and one prefecture-level city (Urumqi) in Xinjiang Uyghur Autonomous Region, China, and sequenced the genomes to high coverage (30–60×) (Fig. S1, Table S1). This dataset enabled us to comprehensively study the population genomics of the XJU people, in particular, their genetic diversity and the genetic landscape of local adaptation. We attempted to comprehensively characterize the genomic variation of XJU to reveal the mechanisms of genomic diversity and phenotypic diversity driven by population admixture.

## RESULTS

### Overview of genetic variation

We obtained 12.03 million single nucleotide variations (SNVs) for the 92 sequenced samples of XJU (2.99 million SNVs on average for each sample), of which 5.56% (668 670) were novel to the Single Nucleotide Polymorphism Database (dbSNP, version 153) (Table S2). According to the annotation based on the software Variant Effect Predictor (VEP, version 96) [10], there were 3097 loss-of-function (LoF) variants and 60 845 non-synonymous variants and other types of functional variants in the sequencing data (Table S3). Detailed information on the data collection, generation, processing and analysis is given in the supplementary data.

### Ancestry make-up and genetic affinity

**Figure 1.**
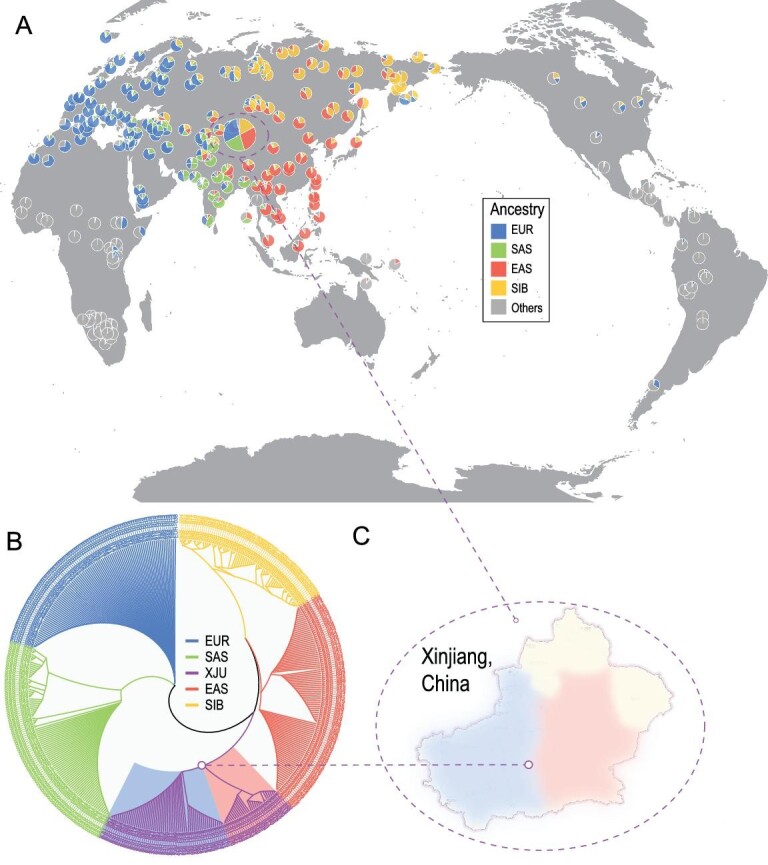
Ancestry make-up and genetic affinity of XJU. (A) Ancestral proportions of worldwide populations at K = 8. The pie chart for XJU is magnified. (B) Individual trees of XJU, EAS, EUR, SAS and SIB populations. Branch lengths were eliminated for better visualization. (C) Simple geographic identification of the XJU individuals in the two subgroups. Review drawing number: GS(2021)6206.

ADMIXTURE [11] analysis (Methods) was applied to the merged XJU dataset [4], together with the Human Origins dataset [12], and the Estonian Biocentre Human Genome Diversity Panel (EGDP) [13]. The global ancestry make-up of XJU was well interpreted by the surrounding populations across the Eurasian continent. When assuming that there are four ancestral groups (K), the ancestral make-up of XJU could be explained by two major ancestral components represented by West Eurasian (mean 47.2%, 26.4%–59.6%) and East Asian populations (mean 46.0%, 33.0%–67.8%) (Fig. S2). At K = 8, XJU shared the majority of their ancestral make-up with populations from EUR, EAS, SAS and SIB, which was in agreement with a previous study [4]. The mean admixture proportions of XJU were 29.5% (15.9%–36.8%), 27.9% (15.5%–51.4%), 22.4% (12.9%–36.6%) and 17.1% (10.1%–25.6%) for the EUR, EAS, SAS and SIB ancestries, respectively, with the remaining <5% related to the early out-of-African ancestries or recent gene flows (i.e. 1.8% American, 0.8% Oceanian and 0.4% African) (Fig. 1A and Fig. S2). These results were fully supported by 10 independent replicates.

The level of genetic affinity for XJU was investigated using fineSTRUCTURE (v2) [14] (Methods), while the EAS, EUR, SAS and SIB populations from the 1000 Genome Project [15] and EGDP were employed as references. The XJU individuals were well clustered together in the individual tree (Fig. 1B), indicating the within-population genetic similarity of XJU individuals, which was likely a result of long-term admixture history. This was further supported by ancestry sharing across populations (Fig. S3). Moreover, two subgroups were also observed in the XJU clade roughly corresponding to the XJU individuals residing in east and west Xinjiang [4] (Fig. 1C). The within-population ancestry sharing inferred by ChromoPainter (v2) [14] as well as the identical-by-state (IBS) showed that XJU individuals had lower genetic ancestry sharing compared with EAS and EUR (Fig. S3), implying the diverse ancestry make-up of XJU.

### Genomic admixture profile of XJU

**Figure 2.**
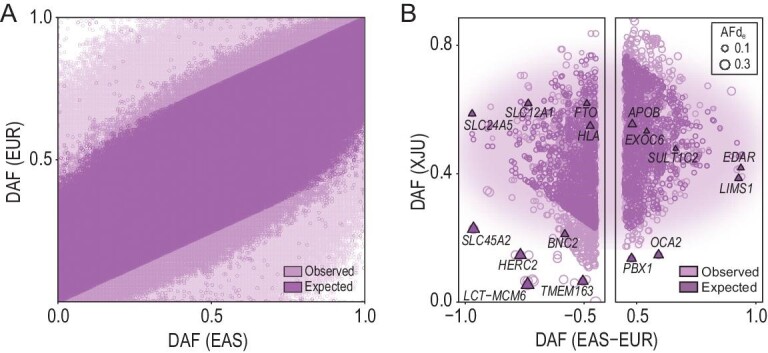
The post-admixture genome-wide frequency profile of XJU. (A) Site frequency spectrum of the frequency profiles among EAS, EUR, XJU and the expected XJU genome. (B) DAF of SNVs showing extreme frequency difference (>0.45, top 1%) between EUR and EAS, and being recorded in the GWAS catalog (v1.0.2). Some well-studied genes are labeled in the figure.

The allele frequency (AF) of the admixed population is expected to be the average of those in the ancestral source populations weighted by the global admixture proportions. Analysis of the site frequency spectrum (SFS) of XJU, EAS and EUR populations showed that XJU had an overall frequency profile close to the expected distribution, while the SFS of EAS and EUR were highly diverged (Fig. 2A and Fig. S4). This pattern was reflected by the small global *F*_ST_ [16] estimated between XJU and the reference populations (*F*_ST [XJU-EUR]_ = 0.031, *F*_ST [XJU-EAS]_ = 0.032 and *F*_ST [EAS-EUR]_ = 0.110). Here, we proposed ‘rules of admixture’, which refer to the general consequences of admixture in shaping the genetic variation in the admixed population.

Nonetheless, frequencies of several local genetic components deviated significantly from the expected values. Of the genetic components of XJU, ∼5% were present in EAS ancestry proportion <41% or >60% and ∼2% of the SNVs across the genome had frequencies indicating a deviation from expectation (AFd_e_) >0.1 (Figs S4–S6). Interestingly, more conserved SNVs showed relatively lower AFd_e_ (*P* < 2.2 × 10^–16^, Wilcoxon rank-sum test), while intron regions were more likely enriched for SNVs with large AFd_e_ (*P* < 7.9 × 10^–12^, Fish exact test) (Fig. S7). Analysis of the ancestry-divergent SNVs recorded in the Genome-Wide Association Studies (GWAS) Catalog [17] also revealed that the majority of the functionally important genetic components followed the ‘rules of admixture’, while some had unexpected frequencies in XJU, including *LCT*–*MCM6*, *SLC45A2* and *HERC2* (Fig. 2B).

### Genetic diversity shaped by population admixture

**Figure 3.**
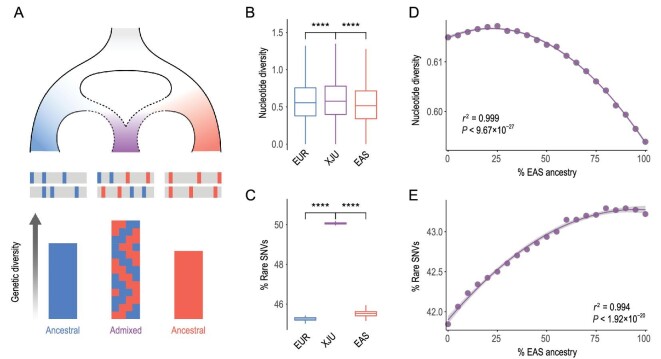
Admixture-driven genetic diversity of XJU. (A) Schematic diagram for admixture-shaped genetic diversity. A combination of genetic components from distinct ancestries results in the higher genetic diversity of an admixed population. (B) Nucleotide diversity (*θ_π_*,/Kb) of XJU, EAS and EUR, which was estimated within sliding windows of 50 Kb in length shifted by 25 Kb across the genome. Fifty individuals were randomly sampled to balance the sample size. Statistical significance from the Wilcoxon rank-sum test: ns: *P* > 0.05; ^*^: *P* < = 0.05; ^**^: *P* < = 0.01; ^***^: *P* < = 0.001; ^****^: *P* < = 0.0001. (C) Rare SNV proportions of XJU, EAS and EUR populations that were estimated as the proportions of SNVs with AF < 0.05. A total of 100 replicates were conducted by random sampling 50 individuals for each association between EAS ancestry and (D) nucleotide diversity as well as (E) rare SNV proportion, which was estimated within sliding windows of 50 Kb in length shifted by 25 Kb across the genome. Median values of nucleotide diversity and rare SNV proportions were used under different ancestry proportions. The curves were fitted using the function ‘lm’ in R.

We investigated the genetic diversity of XJU to understand the influence of population admixture (Methods). Principally, divergent ancestral components introduced by admixture can result in a novel ‘mosaic’ genome and a high level of genetic diversity of the admixed population (Fig. 3A). We found that the nucleotide diversity (*θ_π_*), haplotype diversity (*H*), number of segregating sites (*θ_K_*) and effective population size (*N*_e_) were higher in XJU than in the reference populations (Fig. 3B, Figs S8 and S9). Interestingly, XJU also harbored the highest proportion of rare SNVs (AF < 0.05) (Fig. 3C), followed by EAS, suggesting the contributions of the ancestry-specific SNVs from divergent ancestral populations. This pattern was also reflected by the larger value of *θ_K_* than *θ_π_* in XJU, based on the negative-skew distribution of Tajima's D statistic [18] (Fig. S8). A greater genetic diversity and a higher level of rare SNVs in admixed populations were also confirmed by analysis of the African-American population (Fig. S10).

We further explored how admixture proportion shaped genetic diversity in XJU. We inferred the local ancestry of XJU with Loter [19] (Methods). The ancestry proportion was estimated for the haplotypes within sliding windows of 50 kilobases (Kb) with lengths shifted by 25 Kb across the genome. The genetic diversity of XJU was estimated under different admixture proportions by random sampling of haplotypes with different ancestral origins, with the EAS ancestry ranging from 0% to 100% advanced by 5%. We found that the diversity of XJU haplotypes correlated well with the ancestry proportions (polynomial fitting, *P* < 1.92 × 10^–20^) (Fig. 3D and E, Fig. S11). Specifically, XJU haplotypes with a substantial proportion of both ancestries had the highest genetic diversity, while those mainly contributed to by single ancestral source populations of either EAS or EUR had relatively low genetic diversity. The highest nucleotide diversity and rare SNV proportion for all variants were achieved with ∼25% and ∼90% EAS ancestry, respectively. The correlation of genetic diversity and ancestry composition in XJU was a reflection of admixture-driven micro-evolution.

In addition, we estimated the genetic diversity and effective population size for regional XJU subgroups using the imputed microarray data of ∼1000 XJU individuals with known birthplace information [4] (Methods). A significant correlation between geographic coordinates and genetic diversity was observed as expected (*P* < 1.43 × 10^–3^) (Fig. S12). The mechanism of admixture-driven genetic diversity of the admixed population was further illustrated by the east–west cline of varying effective population size of regional XJU subgroups (*P* < 4.86 × 10^–2^).

### Effects of admixture on functional variants

To investigate the effects of admixture on functional variants, we compared the individual accumulation of LoF SNVs (high impact) and missense SNVs (moderate impact) representing deleterious mutations among XJU, EAS and EUR (Methods). We found that XJU individuals had the most deleterious SNVs among the three populations and were followed by EAS individuals under the dominant model (Fig. S13). In contrast, the number of total deleterious alleles and of homozygotes of XJU individuals are both between EAS and EUR under both the additive and recessive models, while EAS individuals had the most deleterious alleles and homozygotes. These patterns were also observed when the deleteriousness of LoF SNVs and missense SNVs were weighted by their effect size of conservation. The same results were achieved by using all genetic variations, but not only LoF SNVs and missense SNVs, with Combined Annotation-Dependent Depletion (CADD) scores [20] and the Genomic Evolutionary Rate Profiling (GERP) Rejected Substitution (RS) score [21] as the measurements of deleteriousness of variation (Fig. S13, Methods). Population-specific demography may be responsible for the varying numbers of deleterious homozygotes and heterozygotes across populations [22]. The most deleterious heterozygous SNVs being found in XJU individuals should be attributable to their larger number of rare variants introduced by population admixture, while the deleterious homozygous SNVs in EAS individuals may result from the huge number of fixed or nearly fixed SNVs (Fig. S4). The individual genetic load was associated with global ancestry proportion under the additive and recessive models (*P* < 0.03) (Fig. S13), suggesting the admixture-driven genetic diversity of the population.

Analysis of pathways from Kyoto Encyclopedia of Genes and Genomes (KEGG) database [23] revealed that all of the pathways had either higher or similar genetic load in XJU individuals compared with both EAS and EUR under the dominant model, while 31 pathways (8.2%) and 14 genes (0.1%) had significantly more deleterious SNVs in XJU (BH-corrected *P* < 0.05, Wilcoxon rank-sum test) (Tables S4 and S5). For example, pathways related to metabolism, digestion and immunity were identified with higher genetic load in XJU (Fig. S14), suggesting their importance in the local adaptation of XJU post admixture in a new environment. The accumulation of deleterious SNVs in these pathways reflected the admixture-driven genetic diversity in XJU, resulting from the deleterious SNVs introduced by genetic admixture.

### Polygenic adaptation of the admixed genome

High genetic diversity in XJU is expected to further influence the adaptation of XJU. The high genetic load observed in pathways related to metabolism, digestion and immunity suggested the post-admixture accumulation of deleterious variants in local genetic components; this was further supported by the high AFd_e_ of the pathway genes, indicating a potential mechanism of polygenic adaptation.

We applied a modified method based on Gene Set Enrichment Analysis (mGSEA) [24] (Methods) and identified a total of 161 KEGG pathways (42.7%) (Table S6) enriched with genes having high AFd_e_. For example, the metabolism pathway ranked at the top of the enrichment with genes of high AFd_e_ (BH-corrected *P* < 9.79 × 10^–4^) (Fig. S15). There were 699 out of a total 1494 (46.8%) genes in the metabolism pathway that were identified as leading-edge genes, i.e. genes that led to the maximum enrichment score of the corresponding pathway. Among the 699 leading-edge genes, some genes showed significant EAS-biased ancestry (n = 16) and EUR-biased ancestry (n = 11) based on local ancestry inference. For example, *FUCA2* (6q24.2) (Figs S16 and S17), coding an exoglycosidase and related to glycan degradation, was of 65% EAS ancestry which is much higher than the genome-wide average (empirical *P* < 4.93 × 10^–3^). A EUR-biased example is *FHIT* (3p14.2), related to the purine metabolism, which is of only 36% EAS ancestry (empirical *P* < 5.32 × 10^–3^). Notably, the derived allele frequency (DAF) of the missense variant rs6446104 at *FHIT* in XJU (0.522) was lower than that of both EAS (0.592) and EUR (0.566). The signature of a selective sweep on *FHIT* was also identified in XJU by comparing the extended haplotype homozygosity (EHH) between XJU and EUR (Methods), and a significantly higher genetic load at *FHIT* was observed in XJU compared with the reference populations (Table S5).

Moreover, genes in pathways including ‘amino acid metabolism’, ‘lipid metabolism’ and ‘protein digestion and absorption’ were also identified with high AFd_e_ across the genome. For example, *COL11A1* (1p21.1), which encodes components of type XI collagen and is associated with the structure and strength of muscle and skin, was identified with signatures of selection in XJU based on both within- and between-population methods (Methods, Fig. S18). Higher allele sharing was observed between XJU and EAS at the missense variant rs11164663. The haplotype carrying the derived allele (DA) has nearly been lost in XJU (0.016), and this deviated from expectation based on the DA in EAS (0.034) and EUR (0.101). Interestingly, previous studies reported that the DA of rs11164663 is associated with a lower gene expression level of *COL11A1*, and this may be related to some phenotypic variation of muscle and skin in XJU and EAS [25].

**Figure 4.**
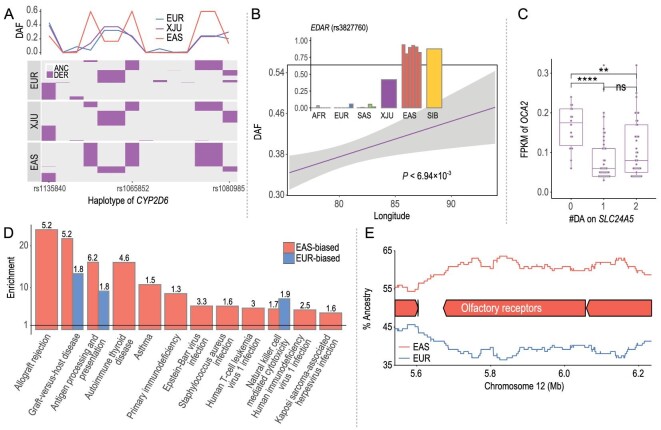
Post-admixture genetic variation and phenotypic diversity of XJU. (A) Haplotype plot of *CYP2D6* in EUR, XJU and EAS, where each horizontal line indicates one haplotype and the x-axis indicates the physical positions. The purple color denotes the derived allele. A similar haplotype structure was observed between XJU and EUR. The plot on the top illustrates the derived allele frequency of each SNV and shows the high similarity between XJU and EUR. (B) Allele frequency of rs3827760 on *EDAR* among the worldwide populations and its association with geographic location across XJU subgroups. (C) Association between the expression level of *OCA2* and the genetic make-up of *SLC24A5* among XJU individuals, indicating the interaction between pigmentation-related components inherited from divergent ancestries. (D) Enrichment of ancestry-biased components in immunity-related pathways (Fisher Exact test, BH-corrected *P*-value < 0.05). The y-axis indicates the odds ratio, and the numbers on the bars are -log_10_ (BH-corrected *P*-values). EAS-biased components were enriched in more immunity-related pathways. (E) EAS-biased local ancestry of *ANO2*, which is one of the olfactory receptor genes. Olfactory perception-related pathways were enriched for EAS-biased components.

The mGSEA approach was further applied to the genes related to absorption, distribution, metabolism, and excretion (ADME) from *PharmaADME* (http://www.pharmaadme.org/). We observed enrichment of genes with high AFd_e_ (BH-corrected *P* < 1.97 × 10^–3^) (Fig. S15 and Table S6). A total of 157 out of the 283 core/extended ADME genes (55.5%) were identified as leading-edge genes. Frequency profile analysis of the core variants in the leading-edge genes indicated relatively low AFd_e_ and small effects of individual ADME genes, suggesting the polygenic adaptation of the metabolism-related pathways in the post-admixture adaptation of XJU. Interestingly, there were two variants of *CYP2D6* (22q13.2), rs1065852 (CYP2D6^*^10) and rs1080985, ranking among the top 0.5% for their high AFd_e_. *CYP2D6* is preferentially expressed in the liver [25] and belongs to the cytochrome P450 superfamily of enzymes. Haplotype analysis revealed a closer relationship between XJU and EUR in *CYP2D6* (Fig. 4A and Fig. S19), which was consistent with findings of a previous study [26]. The DAF values were 0.228 in XJU, 0.602 in EAS and 0.242 in EUR at rs1065852, which is also a missense variant. The P34S substitution at rs1065852 would result in thermal instability together with reduced metabolic activities of drugs [27], indicating lower catalytic activity and thermal stability of *CYP2D6* in XJU than expected. The other variant with high AFd_e_, rs1080985, is a non-coding variant. The DAF values of rs1080985 were 0.299, 0.131 and 0.202 in XJU, EAS and EUR, respectively. A high DAF was associated with lower enzymatic activity and slower drug metabolism [28]. The rs1135840 (CYP2D6^*^2) is another missense variant that may contribute to the decreased metabolic ratio [29], and its DAF of 0.397 in XJU is deviated from the expectation based on EAS (0.243) and EUR (0.434).

In particular, the cytochromes P450 (CYPs) are important for the oxidization and clearance of various compounds in the human body, including clinically used drugs. The genetic basis of the metabolic activities in XJU was determined by both admixture of the divergent genetic components and post-admixture adaptation, meaning that the dose of a drug should be adjusted to take into account the individual's unique metabolism. Decreased metabolic activity of *CYP2D6* was observed in XJU; this enzyme is responsible for the metabolism and elimination of ∼25% of commonly prescribed drugs [30]. Decreased metabolic activity was also inferred for *CYP2C19* based on the frequency profiles of the core variant, i.e. rs4986893, which is also a liver enzyme and processes ∼10% of drugs, including clopidogrel, according to MedlinePlus (https://medlineplus.gov/).

### Phenotypic diversity shaped by admixture

The early contacts of Eurasian populations shaped the phenotypic diversity of the admixed populations in Northwest China during the Bronze Age [4]. Archaeological studies have also revealed the high phenotypic diversity of facial morphology among ancient human samples in Northwest China dating back to ∼3000 YBP [31]. GWASs have found quantitative trait loci such as rs3827760 on *EDAR* (2q12.3) [32] and rs1866188 on *LIMS1* (2q12.3) [33] that are related to the facial morphology of XJU, while *EDAR* is well known for its association with hair thickness [34,35], ear lobe and chin morphology [36], incisor morphology [36] and sweat glands [37,38] in East Asian populations.

Our analysis confirmed that *EDAR* is an admixture-representative (AR) gene (∼47% EAS ancestry) in XJU, as those genes follow the ‘rules of admixture’ and their local ancestry is consistent with the global ancestry estimated from genome-wide data. The AR component was identified by the estimator of population branch statistic (PBS) [39] (Methods, Fig. S20 and Table S7). The haplotypes related to *EDAR* in XJU clustered into two major groups in a network analysis [40] (Methods, Fig. S21) with ancestral source populations of EAS and EUR, indicating the admixture-induced genetic make-up of XJU. The key variant rs3827760 in *EDAR* had DAF values of 0.418, 0.937 and 0 in XJU, EAS and EUR, respectively [41]. Consistent with the geographical distribution of the admixture proportions for the regional XJU groups, a cline of varying DAF was observed for rs3827760 (Fig. 4B, Fig. S22 and Table S8). This suggests that the Uyghur groups residing in western Xinjiang have a higher similarity of phenotypic traits to West Eurasians, and those living in the eastern part are more similar to East Asians [42].

A strong selection signal was found for *EDAR* in EAS based on both the within-population analysis in EAS and a cross-population analysis between XJU and EAS (Fig. S18). However, no signature of natural selection was detected in XJU, as was also proposed in a previous study [43], indicating the relaxation of selection on *EDAR*. Selection relaxation in XJU was further confirmed by comparison between the EAS population and the ancestral EAS population (aEAS) reconstructed from the inferred EAS-ancestry components in XJU (Methods, Fig. S23 and Table S9). High AFd_e_ along the region related to *EDAR* was observed between EAS and aEAS, suggesting the post-admixture differentiation of EAS-ancestry components between EAS and XJU as well as the relaxation of selection in XJU.

Other genes related to the facial morphology of XJU were also investigated. The frequency profiles of *LIMS1* (2q12.3), *PCDH7* (4p15.1), *DCHS2* (4q31.3), *COL23A1* (5q35.3), *SUPT3H*-*RUNX2* (6p21.1) and *GLI3* (7p14.1) were all significantly associated with geographic location; each of the above genes has been reported to be associated with the facial morphology in XJU [32,44] (Fig. S22 and Table S8).

Apart from the functional components related to facial morphology, AR components related to other biological functions were identified, including *ADH1B* (4q23), *ALDH2* (12q24.12) and the *AGO* gene cluster (1p34.3) (e.g. *AGO1*, *AGO3* and *AGO4*) (Fig. S20). *ADH1B* and *ALDH2* may be responsible for alcoholism protection in Asian populations [45,46], and the *AGO* gene cluster is related to RNA interference and silencing [47].

### Interaction of divergent ancestral components

Light skin pigmentation in Eurasian populations has been considered to be the result of convergent evolution during out-of-Africa dispersal. The key components related to skin pigmentation across Eurasian populations were confirmed by GWAS and by experiments on model animals. The identified genetic components include *SLC24A5* (15q21.1) [48–50] and *OCA2* (15q13.1) [51–53], which were the most outstanding genes identified in West Eurasians and East Asians, respectively. Contacts between Eurasian populations resulted in the combination of ancestry-specific components, including those genetic variants that experienced convergent evolution across Eurasia. It was reported that XJU predominantly shows East-Asian-like features of pigmentation [54].

*SLC24A5* was identified as an AR component in XJU with ∼44% EAS ancestry. Both frequency profiles of the key variant rs1426654 and the haplotype network of *SLC24A5* also revealed the balanced contributions from EAS and EUR ancestries (Figs S17 and S19). Similar to *EDAR*, a significant correlation between DAF and the geographic coordinate was also observed for *SLC24A5* (Fig. S22 and Table S8). Another AR component mapped to *SLC12A1* (15q21.1) was located ∼50 Kb downstream of *SLC24A5,* and the intron variant rs11636073 of *SLC12A1* is associated with the pigmentation in EUR populations [55]. The linkage disequilibrium (LD) between rs1426654 and rs11636073 was estimated as *r*^2^ = 0.3 in XJU. Accordingly, signatures of selection were detected in the *SLC24A5*–*SLC12A1* region based on within-population analysis in XJU and cross-population analysis between XJU and EAS (Fig. S18). Interestingly, we observed even higher EHH in XJU than in EUR (Fig. S24). Accordingly, relatively lower genetic diversity was also estimated in XJU compared with EAS (*P* < 2.2 × 10^–16^, Wilcoxon rank-sum test) (Fig. S25).

An ancestry-biased (AB) component (Methods) was identified in the region of *HERC2*–*OCA2*, which had high EAS ancestry (∼62%, empirical *P* < 0.0284) (Fig. S16). The key variant rs12913832 in *HERC2*, associated with eye pigmentation in EUR populations [56,57], showed EAS-biased frequency in XJU (Fig. S17). Analysis of the ancient DNA data showed that the mutation reached a high frequency (>0.5) in EUR before the Bronze Age [58]. Polymorphisms of rs1800414 (allele C) and rs74653330 (allele T) in *OCA2* were associated with skin color in EAS [51–53]. The linkage disequilibrium among the three SNVs in *HERC2*–*OCA2* was relatively weak (*r*^2 ^< 0.03) in all three populations. The DAF distributions of both rs1800414 and rs74653330 were slightly biased, i.e. the DAF of rs1800414 was 0.147 in XJU, 0.592 in EAS and 0 in EUR, while the values were 0.076, 0.019 and 0.005 at rs74653330 in XJU, EAS and EUR, respectively. Although the ancestry make-up of *HERC2*–*OCA2* in XJU was biased towards EAS, the DAF profile at the key variants revealed relatively low allele sharing between XJU and EAS. This was likely due to the relaxation of selection post admixture. The onset of selection on rs1800414 was dated back 15 000 years [52], and a high AF (∼0.5) of rs1800414 was observed in EAS dating back 7000 years (Fig. S17); these dates are much earlier than the admixture events. Moreover, the association was observed between the ancestry of *SLC24A5* and the expression level of *OCA2* (Fig. 4C and Fig. S26), while a relatively higher expression level of *OCA2* was observed for XJU individuals carrying ancestral alleles in rs1426654 on *SLC24A5* (*P* < 5.16 × 10^–5^, Wilcoxon rank-sum test). However, this pattern was not found in *HERC2*, indicating the differences between *HERC2* and *OCA2* in XJU for both AF and expression profiles. This may suggest a new mechanism of local adaptation in the admixed population due to the combination of divergent genetic components with similar biological functions.

### Enrichment of biased ancestry for the perception of stimuli

Enrichment analysis was conducted for genetic components identified with either EAS- or EUR-biased ancestry. Gene ontology (GO) [59] and KEGG enrichment analyses revealed the enrichment of both EAS- and EUR-biased genetic component pathway genes related to immunity, including ‘antigen processing and presentation’, ‘natural killer cell-mediated cytotoxicity’ and ‘graft vs. host disease’ (Fig. 4D and Table S10). Meanwhile, EAS-biased genetic components were also enriched for pathway genes related to immunity (e.g. *HLA*) as well as the sensory perception of smell, e.g. *OR10V1* (11q12.1) and *OR10D3* (11q24.2). There were two LoF/missense variants, rs499037 and rs2466584, in *OR10V1* and *OR10D3*, respectively, with frequency profiles significantly biased toward the EAS ancestry. The DAF of rs499037 in XJU (0.060) is higher than that in both EAS (0.029) and EUR (0); similarly, the DAF of rs2466584 in XJU (0.266) was also higher than in both EAS (0.150) and EUR (0.111). The observation of the highest DAF in XJU compared with the reference populations suggested admixture gain of the functional components and post-admixture local adaptation.

Enrichment of ancestry-biased components for genes related to sensory and immune systems indicated the shared driving forces between XJU and the ancestral populations, and these genetic components may play important roles in the local adaptation of XJU. The enrichment of the EAS-biased components for pathway genes related to olfactory perception could be confirmed by the differentiation between the contemporary EUR population and the ancestral EUR population (aEUR) that was reconstructed from the inferred EUR-ancestry components in XJU (Methods, Fig. S23). Genetic components that were highly differentiated between EUR and aEUR were also enriched toward olfactory receptor (OR) genes (Table S9), e.g. *OR8U1* (11q12.1) and *ANO2* (12p13.31) (Fig. 4E). The DAF of the missense variant of rs11228166 in *OR8U1* in XJU (0.658) was biased toward EAS (0.641) and was much higher than that in EUR (0.369), indicating the large differentiation between XJU and EUR.

Many OR genes identified as having biased ancestry in our study are physically close to each other. A large cluster of OR genes is located downstream of the centromere of chromosome 11 (chr11: 55350000–59600000), while some of them are associated with the variation in human olfactory perception of particular volatile chemicals [60]. Recent studies suggested the Neanderthal ancestry of the centromeric region encompassing the cluster of OR genes [61,62]. Using ArchaicSeeker 2.0, we also identified a large Neanderthal haplotype that segregates in both XJU and EUR populations and spans across the whole region encompassing the cluster of OR genes [63] (Fig. S27). The frequency of the Neanderthal haplotype is ∼2.99% in XJU and ∼12.12% in EUR, while it is almost absent in EAS.

Both OR genes and immune-related genes had the highest levels of genetic variability across the genome [64]. This corresponds to their ability to perceive and process huge amounts of environmental stimuli, including chemical compounds, bacteria and viruses. The sensory and immune systems must evolve rapidly to adapt to the local environment. Although high genetic variability for both sensory and immune systems may influence the local ancestry inference, similar results were also obtained by the inferred local ancestry assuming four ancestral populations (Table S10).

## DISCUSSION

### A valuable population of genetic admixture

It has been suggested that ancient admixture ∼40 000 YBP between modern humans and archaic populations may have provided selective advantages, such as the high-altitude adaptation [65,66]. Other studies have suggested the rapid adaptive evolution in African-American [8] and Latino-American populations [9], which are recently admixed populations formed ∼500 YBP. XJU is the result of multiple contacts between populations across the Eurasian continent during the human dispersal in Northwest China, and areas that experienced multiple waves of admixture events dating back to the Bronze Age (∼3000–4000 YBP) [4]. Consequently, the XJU’s gene pool harbors diverse components, i.e. 15.9%–36.8% EUR, 15.5%–51.4% EAS, 12.9%–36.6% SAS, 10.1%–25.6% SIB, and also <5% related to the early out-of-African populations or recent gene flows. The mixture of divergent ancestral components in a new environment might create novel genetic combinations that have the potential for adaptive evolution. Therefore, XJU is a valuable population for genetic admixture studies. Our study provides new insights into the functional implications of variation and adaptive evolution of admixed genomes.

### Genetic diversity shaped by admixture and its potential impacts on phenotypic diversity

**Figure 5.**
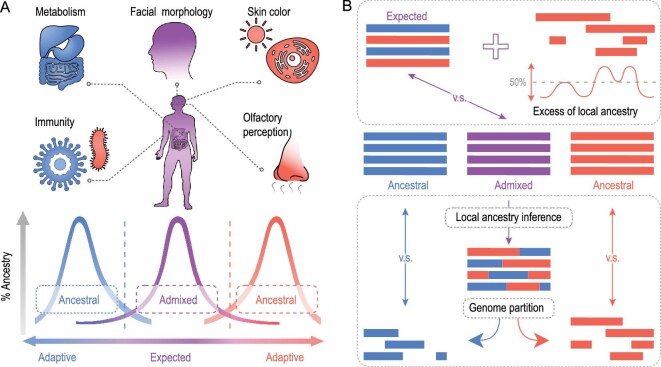
Modeling the post-admixture local adaptation of XJU. (A) States of genetic components in admixed population post admixture. We modeled the states of genetic components in XJU as either expected or adaptive. The purple curve indicates the ancestry composition of XJU, while the other two curves in blue and red denote the composition of the ancestral populations. Two vertical dotted lines indicate the threshold for ‘outliers’ of genetic components in XJU. In our study, we found that EAS-biased components in XJU were related to skin pigmentation, olfactory perception and immunity, while EUR-biased components were associated with metabolism and immunity. Genes related to facial morphology were identified as admixture-representative components in XJU. (B) Schematic presentation of methodology for identifying signatures of local adaptation in an admixed population. We compared the observed and expected genomes of XJU. Candidates of adaptation signals could be indicated by allele frequency and local ancestry proportion deviations from expectation. Comparisons can also be conducted between ancestral populations and reconstructed ancestral populations from the inferred ancestral components.

We investigated the ‘rules of admixture’, i.e. how admixture shapes genome-wide variation in the admixed population. It is expected that admixture on large spatial and temporal scales results in high levels of genetic diversity in the admixed population, due to the genetic components contributed by all the ancestral populations, including both common and rare variants (Fig. 3A). In line with this, we did observe that haplotypes with genetic contributions from both ancestries were most diverse. An increase in rare variants implies more heterozygotes as well as a higher genetic load when considering the dominant model. Here, a large genetic load was estimated for both the genome-wide variation and pathway genes related to metabolism and immunity. These ‘rules of admixture’ highlight the role of admixture in shaping the genetic diversity of admixed populations, and these factors are expected to further influence phenotypic diversity (Fig. 5A). Indeed, large phenotypic diversity has been observed in XJU in traits such as facial morphology, which is one of the most prominent traits of human populations [32,35,36].

### Post-admixture local adaptation

In our data, the majority of genetic components of the admixed population follow the ‘rules of admixture’ and this pattern can be well explained by the ancestral populations. In contrast, local adaptation would result in ancestry-biased local genetic components (Fig. 5A). The admixture gain of functional adaptive components from ancestral populations could facilitate the adaptation of the admixed population, yet such components may also be subjected to random drift under the relaxation of selection. It remains a challenge to detect signatures of local adaptation in admixed populations, due to the confounding effects of admixture that can distort the local haplotype structure by introducing new haplotypes. Different methods were applied in our analysis to obtain a full picture of the putative local adaptations in the admixed genome (Fig. 5B). This included comparative analysis between reconstructed ancestral populations and reference populations, comparisons between the observed XJU genome and reconstructed admixed genomes, and other haplotype-homozygosity-based methods.

A genome-wide scan of the genetic components in XJU revealed that local ancestral components may have had different fates due to their biological properties in both ancestral and admixed populations. For example, EAS-biased local ancestry along with low derived allele sharing indicates the relaxation of selection on *OCA2* in XJU that may be associated with the potential interaction of pigmentation-related genes inherited from distinct ancestries, e.g. *SLC24A5*. Population admixture provided a unique opportunity to study genetic components of divergent ancestry with similar or different biological functions in the same genome. In addition, the admixture gain of genetic components related to immunity and olfactory perception from EAS ancestry indicates the local adaptation of XJU. Both the high genetic diversity and accumulation of mutations in pathways related to metabolism and immunity indicate the potential mechanism of polygenic adaptation post admixture. Both the combination of ancestry-specific genetic components and enrichment of functional components in an admixed population provides new insights into the impact of admixture in human micro-evolution. Driving forces from both the external environment and potentially the compatibility of the admixed genome would further influence the genetic variation present in the admixed genome. We expect that our findings will advance the understanding of admixed genomes, and the results in this study should facilitate further studies of other admixed populations.

## MATERIALS AND METHODS

### Populations and samples

Peripheral blood samples of 92 Uyghur individuals were collected from nine prefectures (Kaxgar, Hotan, Kizilsu, Aksu, Bayingolin, Turpan, Changji, Ili and Bortala) and one prefecture-level city (Urumqi) in the Xinjiang Uyghur Autonomous Region, China. The individuals enrolled in this study were randomly chosen, with sample sizes roughly balanced across all regions. Each individual was the offspring of a non-consanguineous marriage of members of the same nationality within three generations.

### Ethical statement

All procedures performed in studies involving human participants were approved by the Biomedical Research Ethics Committee of Shanghai Institutes for Biological Sciences (no. ER-SIBS-261408), and were in accordance with the 1964 Helsinki Declaration, its later amendments or comparable ethical standards. Informed consent was obtained from all individual participants included in the study. The personal identifiers of all samples, if any existed, were stripped off before sequencing and analysis.

### Genome sequencing and data processing

Whole-genome sequencing, with high target coverage (30–60×) for 150 bp paired-end reads, was carried out on an Illumina HiSeq X Ten platform according to Illumina-provided protocols with standard library preparation at WuXi NextCODE (Shanghai). Each sample was run on a unique lane with at least 90 GB of data that had passed filtering, and read data were quality controlled so that 80% of the bases achieved at least a base quality score of 30. Reads were merged, adaptors were trimmed and sequences were mapped to the human reference genome (GRCh37) using the Burrows-Wheeler Aligner [67]. Variant calling was carried out with the HaplotypeCaller module in the Genome Analysis Toolkit (GATK) [68,69]. Joint-calling of XJU was performed together with samples from another ∼1000 individuals who were also whole-genome sequenced.

### Statistical and population genetic analysis

We applied ADMIXTURE [11] to the merged dataset of XJU together with other reference populations for the global ancestry inference. The shared ancestry profiles and individual trees for XJU were constructed using CHROMOPAINTER and fineSTRUCTURE [14]. AFd_e_ from the genome-wide variation of XJU was quantified based on the difference between the observed allele frequencies (AF_obs_) and those expected (AF_exp_) in XJU. The genetic diversity was estimated using various statistics, including nucleotide diversity (*θ_π_*), haplotype diversity (*H*), numbers of segregating sites (*θ_K_*), the proportion of rare SNVs (AF < 0.05), Tajima's D [18] and effective population size (*N*_e_). Genetic load was estimated based on the SNVs of ‘high’ and ‘moderate’ impact as annotated by VEP (version 96) [10]. The local ancestry inference of XJU was conducted with Loter [19]. Gene Set Enrichment Analysis (GSEA) [70] was modified to identify the enrichment of biologically functional categories in the ranked gene lists. Haplotype networks were constructed using Network (version 10) [40]. We applied the PBS [39] as an estimator to quantify the degree of genetic admixture. We used various methods to detect the potential selective sweeps for XJU, including the excess of local ancestry, allele frequency difference between observed and expected, allele frequency difference between reconstructed ancestral population and reference population, integrated haplotype score (iHS) [71], cross-population extended haplotype homozygosity (XP-EHH) [72], H12 [73] and G12 [74]. Detailed descriptions of the methods are available in the supplementary data.

## DATA AVAILABILITY

Genome data of 92 Xinjiang Uygur samples are available at NODE (https://www.biosino.org/node) with the accession number OEP001377. Software for calculating genetic diversity is available at https://github.com/Shuhua-Group/Theta_D_H.Est/. Software for merging the replicates of ADMIXTURE analysis is available at https://github.com/Shuhua-Group/ADMIXTURE.merge/.

## Supplementary Material

nwab124_Supplemental_File
