# Supplementary material for: Genomic diversity and post-admixture adaptation in the Uyghurs
Source: Natl Sci Rev. 2021 Sep 11;9(3):nwab124. doi: 10.1093/nsr/nwab124 (PMC8953455; doi:10.1093/nsr/nwab124)
Supplement: nwab124_Supplemental_File [file nwab124_supplemental_file.pdf]

Supplementary Data

Genomic diversity and post-admixture adaptation in the Uyghurs

Yuwen Pan, Chao Zhang, Yan Lu, Zhilin Ning, Dongsheng Lu, Yang Gao, Xiaohan Zhao, Yajun Yang, Yaqun Guan, Dolikun Mamatyusupu, Shuhua Xu*

*Correspondence and requests for materials should be addressed to S.X. (Email: xushua@picb.ac.cn).

Contents

Text S1. Populations and Samples 4

Text S2. DNA Sample Preparation and Sequencing 4

Text S3. Alignment, variant identification and filtering 5

Text S4. RNA sample preparation 6

Text S5. Summary of variants calling, evaluation of sequencing error, estimation of genetic distance, and determining ancestral allele 6

Text S6. Phasing and imputation for microarray data sets 9

Text S7. Public and Published Data 10

Text S8. Determining surrogate ancestral populations 11

Text S9. Ancestry analysis with ADMIXTUER 13

Text S10. Ancestry sharing and clustering of XJU individuals 14

Text S11. Population structure and genetic affinity 15

Text S12. Allele frequency spectrum of XJU 16

Text S13. Genome-wide allele frequency deviation 18

Text S14. Local ancestry inference 20

Text S15. Post-admixture genetic diversity of XJU 21

Text S16. Effective population size of XJU 23

Text S17. Genetic burden 24

Text S18. Modified Gene Set Enrichment Analysis 26

Text S19. Within-population signatures of selective sweep (iHS) 27

Text S20. Within-population signatures of selective sweep (H12 + G12) 30

Text S21. Cross-population signatures of selective sweep 33

Text S22. Admixture-representative genetic components 35

Text S23. Reconstructed ancestral populations 38
Text S24. Ancestry-biased genetic components 41
Text S25. Genetic components of XJU with Archaic ancestry 45
Supplementary Figures 50
Figure S1 | Geographical distribution and sample size of XJU groups. 51
Figure S2 | ADMIXTURE analysis of XJU with the reference populations. 53
Figure S3 | Ancestry sharing among XJU and reference populations. 53
Figure S4 | Site frequency spectrum of XJU, HAN, EAS, and EUR. 54
Figure S5 | Distribution of frequency deviation between observed allele frequencies and those of expected. 55
Figure S6 | Local ancestry of XJU.. 56
Figure S7 | Variant conservation and frequency deviation.. 57
Figure S8 | Genetic diversity of XJU and the reference populations.................................... 58
Figure S9 | Effective population size of XJU and the reference populations. 59
Figure S10 | Genetic diversity of ASW and the reference populations................................. 60
Figure S11 | Association between local ancestry and genetic diversity. 61
Figure S12 | Association between geographic coordinates and genetic diversity as well effective population size for regional XJU groups.. 62
Figure S13 | Individual genetic burden of XJU and the reference populations. 63
Figure S14 | Genetic burden of pathways... 64
Figure S15 | Analysis of mGSEA for pathway genes... 65
Figure S16 | Ancestry-biased (AB) genetic components. 66
Figure S17 | DAF of some key variants among world-wide modern human populations and ancient human populations.. 67
Figure S18 | Genomic signatures of selective sweeps in XJU indicated by haplotype-homozygosity-based methods... 68
Figure S19 | Haplotype plot for local genes.. 69
Figure S20 | Identification of admixture-representative (AR) components in XJU. 70
Figure S21 | Haplotype network... 71
Figure S22 | Association between geographic coordinate and allele frequency of XJU individuals... 72
Figure S23 | Genomic signatures of allele frequency deviation between reconstructed ancestral populations and reference populations... 73
Figure S24 | Extended haplotype homozygosity of local genes. 74
Figure S25 | Genetic diversity of local genes.. 75
Figure S26 | Expression profiles of *OCA2* in XJU individuals. 76

Figure S27 | Neanderthal haplotypes along the region of OR gene cluster across different populations. 77

Supplementary Tables 78

Table S1 | Information of the samples for sequencing in this study. 79

Table S2 | Summary of SNVs discovered from deep whole-genome sequencing. 80

Table S3 | SNV counts of different consequence impacts. 81

Table S4 | Pathways of higher genetic burden in XJU individuals. 82

Table S5 | Genes of higher genetic burden in XJU individuals. 87

Table S6 | Pathways enriched for genes of high AFd_e in analysis of mGSEA. 89

Table S7 | Variants with PBS values <-0.5. 95

Table S8 | Association between geographic coordinate and allele frequency of facial-morphology as well pigmentation related key variants among XJU individuals. 96

Table S9 | Enrichment of the ancestry-biased components between reconstructed ancestral population and reference population. 98

Table S10 | Enrichment of the ancestry-biased components. 99

Text S1. Populations and Samples

Peripheral blood samples of 92 Uyghur (XJU) were collected from 9 prefectures (Kaxgar, Hotan, Kizilsu, Aksu, Bayingolin, Turpan, Changji, Ili, and Bortala) and 1 prefecture-level city (Urumqi) in Xinjiang Uyghur Autonomous Region, China. The samples enrolled in this study were randomly collected with samples sizes roughly balanced across all the regions (Fig. S1, Table S1). Each individual was the offspring of a non-consanguineous marriage of members of the same nationality within three generations. All samples were collected with informed consent and approved by the Biomedical Research Ethics Committee of Shanghai Institutes for Biological Sciences. Prior to sequencing and analysis, all samples were stripped of personal identifiers (if any existed). All procedures performed were in accordance with the ethical standards of the Responsible Committee on Human Experimentation (approved by the Biomedical Research Ethics Committee of Shanghai Institutes for Biological Sciences) and the Helsinki Declaration of 1975 (revised in 2000).

Text S2. DNA Sample Preparation and Sequencing

Genomic DNA for the 92 XJU was extracted from the blood samples using QIAGEN DNeasy Blood & Tissue Kit. DNA concentrations were measured with the NanoDrop 2000 (Thermo Fisher Scientific), and sheared with Covaris S220 Sonicator (Covaris) to target of 500–600 base pairs (bp) average size. Fragmented DNA was purified using Sample Purification Beads (Illumina). Adapter-ligated libraries were prepared with the TruSeq Nano DNA Sample Prep Kits (Illumina) according to Illumina-provided protocol. DNA concentrations of the resulting sequencing libraries were measured with the Qubit 2.0 fluorometer dsDNA HS Assay (Thermo Fisher Scientific). Quantities and sizes of the resulting sequencing libraries were analyzed using Agilent BioAnalyzer 2100 (Agilent). The libraries were used in cluster formation on an Illumina cBOT cluster generation system with HiSeq X HD PE Cluster Kits (illumina). Whole-genome sequencing, with high target coverage (30–60×) for 150 bp paired-end reads, was performed in WuXi NextCODE at Shanghai using an Illumina HiSeq X following Illumina-provided protocols. Each sample was run on a unique lane with at least 90 GB pass filter data and the quality of the reads data were controlled for ensuring that 80% of the bases achieved at least a base quality score of 30.

Text S3. Alignment, variant identification and filtering

Per-individual sequence reads were aligned using 'mem' algorithm "bwa mem –M –R @RG\tID:name\tSM:name" in the Burrows-Wheeler Algorithm (BWA) version 0.7.10-r789 (1) to the reference human genome (GRCh37), and then converted to BAM format, sorted by genomic position and indexed using samtools version 0.1.19-44428 (2). To make full use of our computational resources in parallel in the downstream sequence data processing steps, we filtered out the reads with MAPQ < 20 using 'samtools view –q20' and split the single BAM file according to chromosomes.

Picard toolkit version 1.117 (http://broadinstitute.github.io/picard/) was used to mark the potential duplicate reads inherited from library construction step, in which the amplified PCR errors can introduce the wrong variants in variants calling (3, 4). The MarkDuplicates.jar in Picard was used for chromosome-wise duplicates marking per-individual.

Alignments in the combined BAM file were then locally realigned around known insertions/deletions (INDELs) using INDELs reported in the 1000 Genomes Project (KGP) Phase I as the training dataset. Base quality score was recalibrated to reduce the base quality score bias from the sequencer, using INDELs and dbSNP (version 147) reported in KGP Phase I as the training data sets.

HaplotypeCaller module in GATK version 3.2-0-g289df4b (3, 4) was used for SNPs and INDELs calling chromosome-wise simultaneously for each sample, as it is more accurate to call variants in some special region with *de novo* local assembly method, especially in calling INDELs. For population-based analyses, GATK GenotypeGVCFs module was applied to the GVCFs generated in the previous step to call the variants for each chromosome of the combined totally 1025 whole-genome sequencing samples, including 92 XJU and 90 Han Chinese samples (HAN) (5, 6).

The chromosome-wise raw variants were combined to genome-wide raw variants for population-based variants VCFs and individual-based GVCFs. GATK variants quality score recalibration (VQSR) module was used to filter the population based raw SNPs and INDELs, separately. Briefly speaking, VQSR used maximized sensitivity on these variants first, and then used some variants collections as training dataset to estimate the levels of specificity to filter these raw variants. For SNPs filtering, the variants collections contained

HapMap 3.3 genotyping result, OMNI genotyping dataset, KGP Phase I high confident SNPs and dbSNP dataset. For INDELs filtering, the variants collections contained Mills and KGP Phase I gold standard INDELs.

After VQSR, we constructed the universal mask as described elsewhere (7). It is a sample independent mask that identifies complex regions in the human reference genome where variant calling can be challenging. We filtered variants with the universal masks including regions with (a) low mappability mask used in the Simons Genome Diversity Project (SGDP) (7); (b) low complexity mask encompring regions made by mDUST, and homopolymers and repeat regions obtained from UCSC (8).

Text S4. RNA sample preparation

RNA sample for 208 XJU (data unpublished, also collected with sample sizes roughly balanced across all the regions in Xinjiang) was extracted using PAXgene Blood RNA Kit (QIAGEN). RNA sequencing, with high target coverage (30–60×), for 125 bp pair-end reads, was performed in WuXi NextCODE at Shanghai using Illumina HiSeq2500 platform following Illumina-provided protocols. Raw sequence reads were trimmed by Trim Galore (0.4.3) (http://www.bioinformatics.babraham.ac.uk/projects/trim_galore/), followed by mapping to the reference human genome (GRCh37) using STAR (9). Gene and transcript expression were quantified and normalized to FPKM (fragments per kilobase of transcript per million mapped reads) using RSEM (10).

Text S5. Summary of variants calling, evaluation of sequencing error, estimation of genetic distance, and determining ancestral allele

We discovered totally 29,394,613 single nucleotides variations (SNVs) among all of the 1025 individuals. There are 12,031,412 SNVs identified in 92 XJU samples, of which 5.56% of the SNVs are novel to dbSNP (version 153) (Table S2). The transition and transversion ratio (Ti/Tv) for XJU genomes is 2.02. We used the VEP (version 96) (11) to predict the functional effect of variants obtained from XJU, including the consequence impact, Combined Annotation–Dependent Depletion (CADD) score (12), and Genomic Evolutionary Rate Profiling (GERP) Rejected Substitution (RS) score (13). There are 3,097 Loss-of-Function (LoF) variants, 60,845 non-synonymous variants and other types of functional variants in the sequencing data (Table S3).

Significantly more segregating sites were observed in XJU compared with HAN (*P* <2.2×10^{-16}, Wilcoxon rank sum test) (Fig. S5.1A), which was conducted by sampling 50 individuals from each group at random without replacement for 100 times. Meanwhile, XJU individuals had significantly more heterozygotes but smaller number of alternative alleles or homozygotes (*P* <2.2×10^{-16}, Wilcoxon rank sum test) (Fig. S5.1B). It was consistent with the pattern revealed by runs of homozygosity (ROH) (Fig. S5.1C), which was defined when two haplotypes in an individual DNA carried identical sequences across a region. We analyzed the data set using PLINK1.9 (14) and chose the ROH with size of 1 mega base (Mb) or larger. The relationship of individual number of ROH and cumulative length ROH was inferred. On average, XJU individuals have smaller number of ROH (*P* <4.14×10^{-9}, Wilcoxon rank sum test) but higher cumulative length of ROH compared with HAN (*P* <2.12×10^{-4}, Wilcoxon rank sum test).

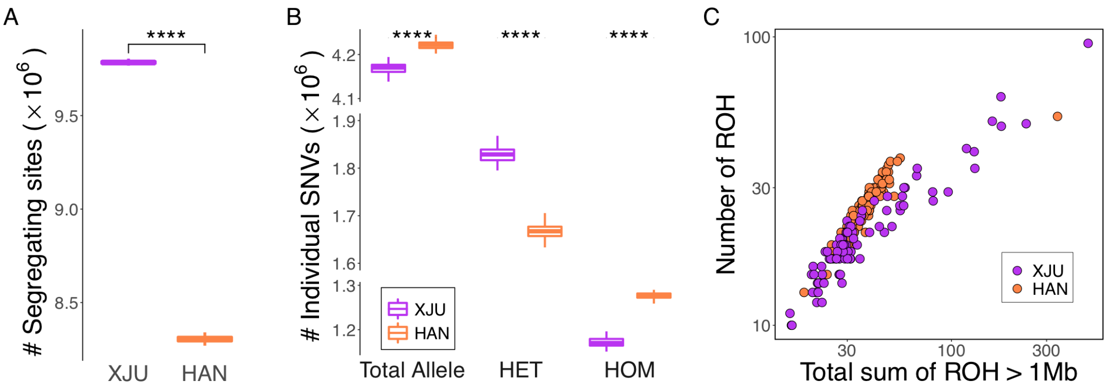

Figure S5.1 | Summary statistics for genome-wide variation of XJU and HAN
A. Numbers of segregating sites in XJU and HAN. We counted variants among 50 random sampled individuals from each population, with totally 100 replicates; **B.** Individual counts of heterozygotes (HET), homozygotes (HOM), and total alternative alleles (Total Allele) in XJU and HAN; **C.** Distribution of numbers of runs of homozygosity (ROH) per individual and cumulative length of ROH in XJU and HAN. Significance test was performed based on Wilcoxon rank sum test: ns: p > 0.05; *: p <= 0.05; **: p <= 0.01; ***: p <= 0.001; ****: p <= 0.0001.

Sequencing error was further evaluated for XJU by comparison between sequencing data and microarray data of the same individuals (15). Totally 789,910 overlapping loci were used. For each individual, we counted the numbers of loci that had different genotypes between sequencing and microarray data as well the total allele differences across the genome. The mismatch rate was estimated as ~0.5% for the genotype difference between

sequencing and microarray data, while it was ~0.3% for the allele difference (Fig. S5.2).

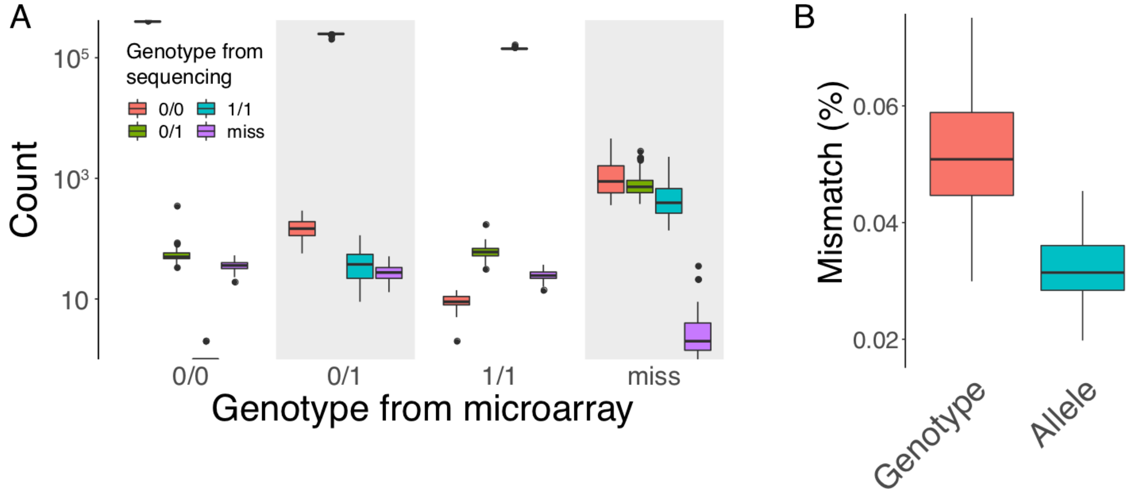

Figure S5.2 | Evaluation of the sequencing error for XJU individuals.
A. Comparison of genotypes on the same loci between the sequencing and microarray data sets of XJU individuals. The x-axis indicates different genotypes from the microarray data, and colors of boxes indicates the genotypes from the sequencing data; **B.** Mismatch rates of genotype and allele between sequencing and microarray data sets for XJU individuals. Genotype mismatch rate for each XJU individual was calculated as the number of loci with different genotypes divided by the total number of loci identified in both data sets. Allele mismatch rate was measured as the number of allele differences divided by the total number of alleles.

Genetic distance and recombination rate for each locus was calculated based on the published recombination map, accessed from http://hapmap.ncbi.nlm.nih.gov/donwloads/recombination/2011_phaseII_B37/. PLINK1.9 was employed to calculated the genetic distance and recombination rate, using the command "--cm-map".

To determine the ancestral and derived alleles for SNVs obtained from our sequenced genomes and public data integrated in out analyses, we downloaded the ancestral sequence from the 1000 Genome database, which were inferred from 6 primates (ftp://ftp.1000genomes.ebi.ac.uk/vol1/ftp/phase1/analysis_results/supporting/ancestral_alignments/). We annotated the ancestral base for each locus in our analyzed data sets according to the ancestral sequence. Only loci with called ancestral allele (A, T, C, G or a, t, c, g) that matching the "REF" or "ALT" allele in the VCF files would be remained, and the derived allele would be the other allele. Finally, 28,655,389 loci with ambiguous

derived allele were remained in our sequencing data set of XJU.

Text S6. Phasing and imputation for microarray data sets

Phasing was performed using SHAPEIT2 (16) for the sequencing data of totally 1025 samples without reference panel, with all parameters of default set. Subset of the phased data set including 92 XJU and 90 HAN samples was used for the downstream analyses. We further imputed two microarray data sets of XJU from the previous study (15). The reference panel for imputation was merged from the 1000 Genome Project (n=2504) and the phased data set of 1025 samples using IMPUTE2 (17) with the command "-merge_ref_panels". Totally 29,122,345 SNVs with overlapping physical positions to our sequencing data set were left in the merged reference panel.

Initial quality control was conducted for the two microarray data sets before imputation. There remained 839,819 loci and 241 samples in the Affymetrix data set, and 865,027 loci and 726 samples in the Illumina data set, after removing loci and individuals with missing data >10% using PLINK1.9 (14) (--geno 0.1 --mind 0.1). Pre-phasing was applied to the two data sets respectively using SHAPEIT2 with the merge the reference panel. There left 821,977 and 820,412 loci respectively for the Affymetrix and Illumina data sets. The imputation procedure was conducted separately for the two data sets using IMPUTE2, with the imputation interval set as 5 Mb in length (the "-int" parameter). The accuracy of imputation is ~98%, estimated as the overall concordance by cross-validation (Fig. S6.1). We got 28,710,768 and 29,014,856 loci respectively for the Affymetrix and Illumina data sets, after imputation and following filtration of loci with missing rate >10% as well those failed the Hardy–Weinberg disequilibrium ($P<10^{-6}$). The imputed data sets were then merged together after re-phasing, which was conducted in the same way as pre-phasing, remaining totally 27,925,304 loci.

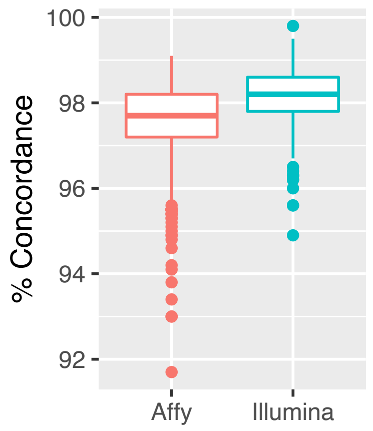

Figure S6.1 | **Concordance of the genotype imputation.**

Text S7. Public and Published Data

Affymetrix Human Origins genotyping dataset (HumanOrigins)

Affymetrix Human Origins genotyping dataset (18) was obtained with a signed letter delivered for restrictive full data access, and was used for admixture analyses under context of worldwide populations. We used the 'Simple population ID' instead of 'Verbose population ID' to assign the population identification for each individual. Finally, 49 populations from Africa, 60 populations from WestEurasia (EUR), 22 populations from SouthAsia (SAS), 23 populations from CentralAsiaSiberia (SIB), 22 populations from EastAsia (EAS), 3 populations from Oceania, 11 populations from SoutheastAsian, and 24 populations from America, and totally 2,419 individuals from 214 populations were employed.

1000 Genomes Project Phase III

The variants from 1000 Genomes Project Phase III (KGP) (19) were downloaded from (http://ftp.1000genomes.ebi.ac.uk/vol1/ftp/release/20130502/). We chose CHB, CEU, and GIH in the KGP data set to respectively represent the ancestral EAS, EUR, and SAS populations contributed to the formation of XJU, largely according to the results of principal component analysis (PCA) and ADMIXTURE (20) analysis (Fig. S8.1). We compared the allele frequency (AF) profiles of HAN and CHB, and 1,423 SNVs with frequency difference >0.2 between HAN and CHB were removed to eliminate the potential bias.

Estonian Biocentre Human Genome Diversity Panel

The data set of Estonian Biocentre Human Genome Diversity Panel (EGDP) (21) was download from (www.ebc.ee/free_data). There are 108 SIB samples in the EGDP data set sampled from multiple Siberian populations. Totally 73 SIB samples were employed in our analyses to represent the ancestral SIB population, after filtering 34 “South Siberia” and 1 “West Siberia” samples due to their complex ancestry makeups (Fig. S7.1).

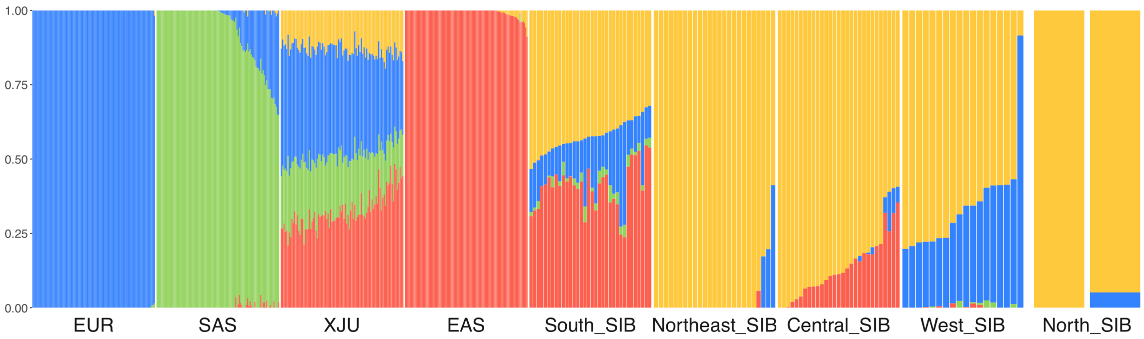

Figure S7.1 | ADMIXTUE analysis for selection of SIB samples.

HAN Chinese genomes

To ensure the potential batch effect to be minimal in comparative analyses, we employed 90 HAN Chinese genomes (30–60×) (5, 22) sequenced with the same platform and procedure as that for XJU.

Ancient human sequencing and genotyping data sets

Summary data of allele frequency for ancient European populations were accessed from the database PGG.SNV (https://www.pggsnv.org) (23). The ancient human genomes integrated in PGG.SNV were collected from the previous published studies (24-30), including 999 ancient European genomes, representing 107 ancient European populations. The data set of ancient East Asian samples was downloaded from (https://bigd.big.ac.cn/gsa-human/browse/HRA000123) (31), including 24 ancient East Asian samples, representing 12 ancient East Asian populations.

Text S8. Determining surrogate ancestral populations

Principal Component Analysis (PCA) and ADMIXTURE (20) analysis were conducted using the merged data set of XJU sequencing data, EUR, SAS, and EAS populations from KGP, as well SIB population from EGDP, including totally 17 populations. We pruned the

merged data set by excluding one variant from each pair closer than 50 kilobases (Kb) using PLINK1.9 (--bp-space 50000) to eliminate the influence of the linkage disequilibrium (LD), leaving totally 44,121 SNVs. We ran ADMIXTURE using the merged data set with all parameters set as default. The number of ancestral cluster (K) was 4. PCA was then performed using EIGENSOFT (v7.2.1) (32) for XJU together with other populations of the same ancestries.

Ancestry makeup of XJU is well explained by the ancestries of EAS, EAS, SAS, and SIB according to the ADMIXTUE analysis (Fig. S8.1A). Relatively homogenous EUR ancestry was observed in CEU (98.6%) and GBR (99.0%). CEU and GBR were clustered together in the PCA plot, while both of them were also on the direct line formed by XJU samples (Fig. S8.1B). Among the SAS populations, both GIH and PJL lie on the line of XJU samples (Fig. S8.1C), while GIH had higher SAS ancestry (84.7%) compared with PJL (76.0%), suggesting GIH as a better surrogate ancestral population. For the EAS populations, both CHB and JPT were on the line of XJU samples (Fig. S8.1D), and higher EAS ancestry was identified in CHB (83.9%) compared with JPT (72.6%). Finally, CEU, GIH, and CHB were selected as surrogate ancestral populations for XJU, respectively representing the ancestral EAS, EUR, and SAS populations contributed to the formation of XJU.

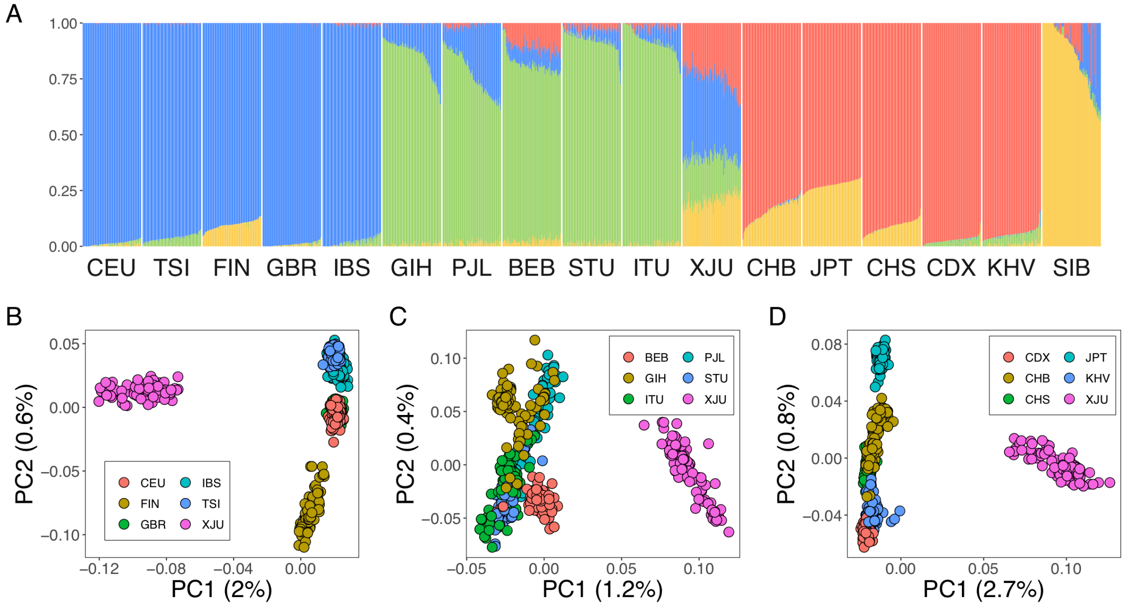

Figure S8.1 | ADMIXTUE analysis and PCA for surrogate ancestral population selection.

A. ADMIXTURE analysis of XJU together with EUR, SAS, EAS, and SIB populations; **B.** PCA of XJU and EUR populations; **C.** PCA of XJU and SAS populations; **D.** PCA of XJU and EAS populations.

Text S9. Ancestry analysis with ADMIXTUER

We applied the model-based ancestry estimation method ADMIXTURE on the merged data set of XJU and other reference populations, including the microarray data sets of XJU (967), HAN (90), HumanOrigins (2,419), and SIB (73) from EGDP, consisting of totally 3,549 samples from 217 populations across the world. ADMIXTURE inferred individual genetic ancestry conditional on a specific number of ancestral clusters (K). We used it mainly to re-analyze the population structure of XJU on the context of the world-wide populations. We then partitioned the genome into non-overlapping 50 Kb windows and pruned the merged data set by random sampling one SNV within each window to eliminate the influence of LD, remaining totally 33480 SNVs for the ADMIXTUER analysis. We ran ADMIXTURE for the merged data set under unsupervised mode, assuming K ranging from 2 to 10. Totally 10 replicates were conducted for the same K using different sets of genetic variants generated in the same way.

As individual ancestry proportions varied, we evaluated the consistency of the results across all the replicates. Consensus of ancestry components from independent replicates were determined based on the pairwise correlation of the inferred individual ancestry proportions. For any two replicates, we calculated the pairwise correlation coefficients between the individual ancestry proportions of ancestry components from different replicates. Given an ancestry component in any replicate, we took another one from a different replicate as its best match if their correlation coefficient was at least 0.5 higher than other pairs of ancestry components. Further, two replicates were considered as consensus if every ancestry component had its own best match. The supporting rate for each ancestry component was then calculated as the maximum proportion of replicates that had the corresponding ancestry component matching to each other. And the final estimate of the individual ancestry proportions was estimated by averaging all the consensus replicates. The supporting rate for each ancestry component estimated under the world-wide context with K≤9 was 100%, but only one replicate had different patterns of SIB ancestry at K=10.

The global ancestry makeup of XJU was well interpreted by the surrounding

populations across the Eurasian continent. At K=4, the ancestral makeup of XJU could be well explained by 2 major components represented by EUR (mean 47.2%, 26.4–59.6%) and EAS (mean 46.0%, 33.0–67.8%). At K=8, XJU shared majority of their ancestral makeup with populations from EUR, EAS, SAS, and SIB. The mean admixture proportions of XJU were 29.5% (15.9–36.8%), 27.9% (15.5–51.4%), 22.4% (12.9–36.6%), and 17.1% (10.1–25.6%) for the EUR, EAS, SAS, and SIB ancestries, respectively, with the remaining <5% related to the early Out-of-African ancestries or recent gene flow (i.e., 1.8% American, 0.8% Oceanian, and 0.4% African) (Fig. S2).

Further, the global ancestry makeup of XJU was re-estimated using the sequencing data, together with the surrogate ancestral populations of EAS and EUR, assuming the K=2. And K=4 when surrogate ancestral populations of SAS and SIB were also employed. The merge data sets were pruned in the same way, leaving 52,990 and 52,789 SNVs respectively for 2-reference and 4-reference analyses. And results were consistent across 10 independent replicates. The estimated global ancestry proportions were 51.4% EUR (36.0–68.0%) and 48.6% EAS (32.0–64.0%) for XJU under the 2-way admixture model, and they were 35.6% EUR (24.2–43.9%), 32.6% EAS (18.8–48.8%), 18.8% SAS (11.1–32.9%), and 13.0% SIB (8.2–19.7%) under the 4-way admixture model (Fig. S2).

Text S10. Ancestry sharing and clustering of XJU individuals

Ancestry-sharing matrix was constructed using CHROMOPAINTER v2 (33) following the recommended procedures. We merged sequencing data of XJU together with the reference populations of EUR, EAS, SAS, and SIB, including totally 470 samples. SNVs in the merged data set with AF <5% or >95% among all the populations were removed to accelerate the program, remaining totally 5,796,018 SNVs across the genome. The initial genome-wide average "switch" rate (n) and global "emission" rate (M) were firstly estimated on the merged data set using CHROMOPAINTER after 10 iterations of Expectation-Maximization (E-M) algorithm. We then average the results over all individuals and chromosomes, weighted by the number of SNVs on each chromosome. We re-run CHROMOPAINTER to have all the individuals "painted" by each other using the 2 estimated parameters, with others set to the software defaults. Ancestry sharing was evaluated based on the output of co-ancestry matrix indicated by either sharing chunk counts or chunk lengths.

Similar patterns were observed for both chunk-counts and chunk-lengths based analyses (Fig. S3). Population-specific profiles of ancestry sharing were observed among all the populations. Within-population ancestry sharing had higher level than that of cross-population, indicating the genetic similarity across individuals within each population. On the other hand, XJU individuals had relatively lower ancestry sharing with each other compared with the reference populations, suggesting the diverse ancestry makeup among XJU individuals. Higher ancestry sharing was identified while assigning XJU as ancestry recipient and another reference population as ancestry donor, compared with the opposite scenario. It was consistent with the finding in a previous study that majority of the genetic component in XJU could be found in the reference populations (34).

Individual tree was further constructed using fineSTRUCTURE v2 (fs) (33) based on the co-ancestry matrix of chunk counts. Visualization of the tree was done by the R package "ggtree" (35).

Text S11. Population structure and genetic affinity

PCA was performed for XJU using the same data set as for the ADMIXTUER analysis. In the PCA plot, XJU samples were surrounded by all the reference populations (Fig. S11.1A), which was consistent with the previous finding (15).

Pairwise global F_{ST} (36) was further calculated using the whole genome sequencing data of XJU and the surrogate ancestral populations. Relatively small F_{ST} values were estimated between XJU and other reference populations (Fig. S11.1B, Table S11.1), indicating the sharing genetic makeup. Meanwhile, the closest genetic distance was observed between XJU and SAS as well SIB ($F_{ST\ [XJU\text{-}EUR]}$ =0.0308, $F_{ST\ [XJU\text{-}EAS]}$ =0.0318, $F_{ST\ [XJU\text{-}SAS]}$ =0.0195, and $F_{ST\ [XJU\text{-}SIB]}$ =0.0284).

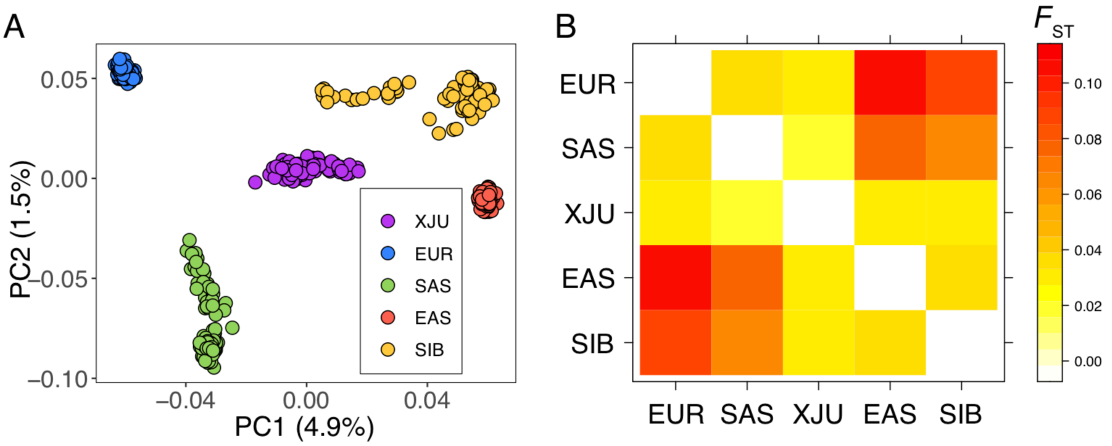

Figure S11.1 | Ancestry analysis and genetic affinity of XJU.

A. PCA of XJU and surrogate ancestral populations; **B.** Heatmap of pairwise global F_{ST} estimated among XJU and surrogate ancestral populations. Global F_{ST} was estimated using the whole-genome sequencing data.

Table S11.1 | Pairwise global F_{ST} among XJU and the reference populations.

	XJU	EAS	SIB	EUR	SAS
XJU	-	0.0318	0.0284	0.0308	0.0195
EAS	(0.0315, 0.0321)	-	0.0344	0.1067	0.0738
SIB	(0.0281, 0.0287)	(0.0339, 0.0348)	-	0.0875	0.0657
EUR	(0.0305, 0.0311)	(0.1060, 0.1076)	(0.0869, 0.0882)	-	0.0349
SAS	(0.0193, 0.0197)	(0.0732, 0.0745)	(0.0651, 0.0663)	(0.0345, 0.0353)	-

Numbers in the brackets indicate the 95% confidence interval of the F_{ST} statistics, which were estimated based on 100 moving block bootstraps (37).

Text S12. Allele frequency spectrum of XJU

We investigated the site-frequency-spectrum (SFS) of XJU and other reference populations, including HAN, EAS, and EUR populations (Fig. S4). Distributions of both minor allele frequency (MAF) and derived allele frequency (DAF) revealed the important role of low frequency SNVs in XJU. There observed more low frequency SNVs (AF <0.01) in XJU compared with other reference populations, followed by HAN and EAS, while relatively lower proportion of SNVs with AF >0.05 were observed in XJU. Higher proportion of SNVs were found in EAS and HAN with DAF almost fixed (DAF >0.99), while it was the lowest in XJU. Similar patterns were also observed for the genome-wide deleterious SNVs, which were defined as those with either high and moderate consequence impact, CADD score (12) >15, or GERP score (13) >2.

The frequency profiles were further compared among different populations based on

the 2-dimensional (2D) SFS (Fig. 2A). We estimated the expected allele frequencies (AF_{exp}) of XJU by averaging those in the surrogate ancestral populations, weighted by the global admixture proportions (EAS 49%, EUR 51%). The results showed that XJU had observed frequency profile similar to that of expected and was closely related to the reference populations, while the frequency profile between EAS and EUR were highly divergent.

We calculated the allele frequency deviation from expectation (AFd_e) for genome-wide SNVs of XJU as the absolute difference between the observed AF (AF_{obs}) and AF_{exp}. Among the 12 million SNVs identified in XJU, EAS, and EUR populations, half of them had AFd_e <0.01, and only ~2% SNVs had AFd_e >0.1, ~0.01% SNVs >0.2 (Fig. S5). SAS and SIB populations were further employed to re-calculate the AF_{exp} of XJU under the 4-way admixture model, and the AF_{exp} of XJU was constructed in the same way. Comparison of AF_{exp} and AF_{obs} of XJU revealed the same pattern as was observed, while it was relatively more conserved. Among the over 10 million SNVs identified in all the 5 populations, about half of them had AFd_e <0.009, <1% SNVs had AFd_e >0.1, and ~0.02% SNVs had AFd_e >0.16 (Fig. S5).

We grouped the genome-wide SNVs of XJU into different classes according to their consequence impact, CADD score, and GERP score. It was found that SNVs with high conservation had significantly lower AFd_e ($P < 3.4\times10^{-4}$, Wilcoxon rank sum test) (Fig. S7), which was as expected. All the SNVs, according to their functions and positions relative to genes, were classified into transcript, genic, intergenic, exon, intron, CDS, 5'-UTR, 3'-UTR, start codon, and stop codon based on the GTF annotation from Ensembl (release 90). Enrichment analysis (Fish exact test) revealed that SNVs with large AFd_e (empirical P <0.01) were enriched in intron, genic, and transcript regions, while under-representation of SNVs with large AFd_e were observed in intergenic regions and also genetic components with high conservation (Fig. S7).

To evaluate the functional impact of population admixture on XJU, we intersected all the SNVs identified among XJU, EAS, and EUR with significant associations reported in the GWAS Catalog (38). We got totally 69,654 SNVs, with majority of them having AF under expectation, and only 1,050 SNVs (1.5%) had significant AFd_e (empirical P <0.01). There were 567 out of the 1,050 SNVs (54%) had AF profile biased toward EUR, and the other 483 (46%) toward EAS. Further, we investigated SNVs with AF difference >0.45

(top ~1%) between EAS and EUR. About 2.67% SNVs (64/2397) had significant AFd_e, with 54 of them having AF profile deviated toward EAS and the other 10 toward EUR, involving some well-known genes including *LCT–MCM6*, *SLC45A2*, and *HERC2*.

Text S13. Genome-wide allele frequency deviation

Allele frequency deviation from expectation (AFd_e) was quantified for the genome-wide variations of XJU. AFd_e was calculated as the absolute difference between the observed allele frequencies (AF_{obs}) and those of expected (AF_{exp}) in XJU. The AF_{exp} of XJU was estimated as the admixture-proportion-weighting average of the frequencies in ancestral populations. And the weights were assigned as the global admixture proportions estimated by the ADMIXTURE analysis (49% EAS, 51% EUR).

AFd_e was calculated as $|AF_{obs} - AF_{exp}|$ for all the SNVs identified among the 3 populations. SNVs with AF <0.01 or >0.99 in all the 3 populations were dropped, remained totally 9,004,531 SNVs. The significance of AFd_e for each SNV was further investigated based on the AFd_e rank. To eliminate the potential effect of minor allele frequency (MAF), we grouped all the variants according to their expected MAF (MAF_{exp}) into bins of size 0.01. Meanwhile, the potential influence of the allele frequency difference between the two reference populations ($AF_{EAS-EUR}$) was also under control. Variants were binned into ranges of $AF_{EAS-EUR}$ [0.0,0.01), [0.01,0.02), [0.02,0.03), [0.03,0.04), [0.04,0.05), [0.05,0.06), [0.06,0.10), [0.10,0.15), [0.15,0.20), [0.20,0.30), and [0.30,1.0], which also ensured sufficient variants within each bin. We got totally 505 bins after removing the empty sets. The empirical *P*-value for each SNV was estimated within the corresponding bin as the percentage of SNVs with larger AFd_e value. We grouped all the SNVs according to their empirical *P*-values into different bins, and distributions of AFd_e values across all the bins revealed significant shift of AFd_e for SNVs ranking the top 1% ($P_{top\ 1\%\ vs.\ top\ 2–3\%} < 2.2\times10^{-16}$, Wilcoxon rank sum test) (Fig. S13.1A). Therefore, we took empirical *P*-value <0.01 as the threshold to identify SNVs of large AFd_e, obtaining totally 89,798 SNVs.

Another 2 reference populations, SAS and SIB, were further employed to investigate the genome-wide AFd_e under the 4-way admixture model. The AF_{exp} of XJU was re-estimated by averaging the frequencies of EAS, EUR, SAS, and SIB, weighted by the global admixture proportions (35% EUR, 33% EAS, 19% SAS, and 13% SIB). AFd_e under the 4-way admixture model was calculated as $|AF_{obs} - AF_{exp}|$ for all the SNVs identified

among the 5 populations. We excluded SNVs with AF <0.01 or >0.99 and obtained totally 8,672,885 SNVs for the downstream analyses. The significance of AFd_e for each SNV was estimated in the same way. We grouped all the variants by their MAF_{exp} into bins of size 0.01, and the empirical *P*-value for each SNV was estimated as the percentage of SNVs with larger AFd_e value within the corresponding bin. We grouped all the SNVs according to their empirical *P*-values into different bins. And significantly larger AFd_e values were also observed among SNVs ranking the top 1% ($P_{\text{top 1\% vs. top 2–3\%}} < 2.2\times10^{-16}$, Wilcoxon rank sum test) (Fig. S13.1B). There are 86,705 SNVs with empirical *P*-value <0.01.

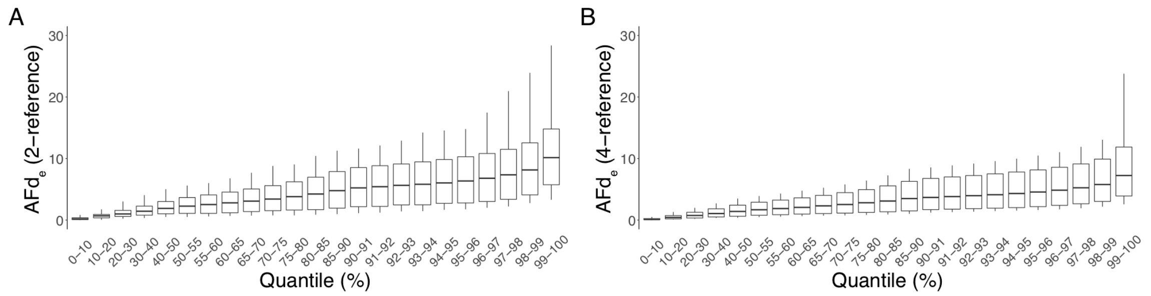

Fig. S13.1 | Distribution of AFd_e in XJU across the quantile bins.
AFd_e and quantiles were estimated using either **A.** 2 reference populations or **B.** 4 reference populations. SNVs were grouped into bins of similar quantiles. Extreme shifts of AFd_e were observed in both analyses for SNVs ranking the top 1%.

Furthermore, AFd_e was characterized for regional components. We partitioned the genome into sliding windows of 50 Kb in length shift by 25 Kb and calculated the proportions of SNVs with large AFd_e (empirical P <0.01) in each window. We chose this window size primarily due to more than two-third of the sliding windows with SNV counts >50, and because the mean value of intermarker linkage disequilibrium (LD) across the genome decays below one-third of its maximum value (Fig. S13.2). Since the variant density varied considerably across the sliding windows, we grouped all the windows by the variant counts into bins of size 10. Windows with <60 SNVs were grouped together, as well those with >280 SNVs, to ensure sufficient windows within each bin. Meanwhile, windows with <50 variants were removed from the further analyses, leaving totally 104,190 sliding windows. We got 23 bins with variant counts ranging from 60 to 280 advanced by 10. The empirical *P*-value for each window was estimated within the corresponding bin as the percentage of windows with larger proportion of large AFd_e SNVs.

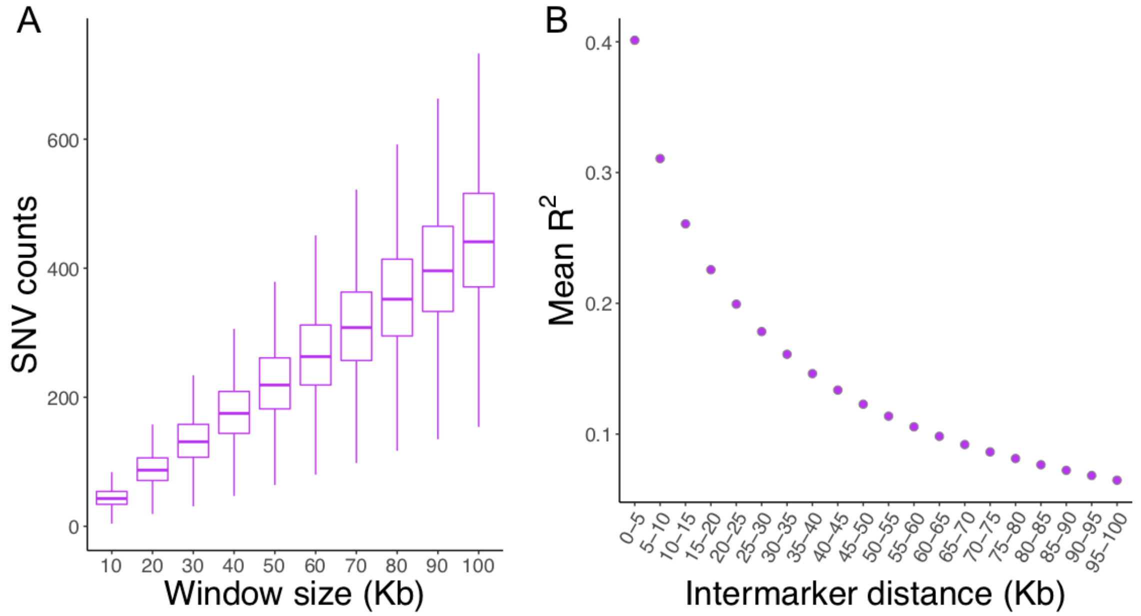

Fig. S13.2 | Evaluation of the SNV counts and LD decay for different window sizes.
A. Distributions of SNV counts for sliding windows of different sizes in XJU; **B.** Mean decay of the R^2 measure of LD between pairs of loci in XJU.

We investigated AFd_e for the genome-wide genes in the same way. Gene annotations including the gene boundary were accessed from Ensembl (v96) using the R package "biomaRt" (39). All of the annotated genes were partitioned into sliding windows of 50 Kb in length shift by 25 Kb increment. For genes shorted than 50 Kb, the gene region would be extended to 50 Kb on both side by the same length. We calculated the proportions of SNVs with large AFd_e in each window, and grouped all the windows by their variant counts into bins of size 10. Windows with <60 SNVs were grouped together, as well those with >280 SNVs. Meanwhile, windows with <50 variants were removed, leaving totally 91,145 windows. Sliding windows were ranked according to their empirical *P*-values, which were calculated in the same way as mentioned above. Finally, for genes with multiple windows, we left only the one with the smallest empirical *P*-value. And 49,869 genes were remained for the 2-reference analysis, while it was 49,461 for the 4-reference analysis.

Text S14. Local ancestry inference

Loter (40) was used for the local ancestry inference of XJU, since it was proved to have

higher accuracy for ancient admixture (>100 generations) and multi-way admixture compared with other tools. We run local ancestry inference for XJU under both the 2-way and 4-way admixture models. There were 99 individuals random sampled from both EAS and EUR populations for the 2-reference analysis. For the 4-reference local ancestry inference, 73 samples were sampled from EAS, EUR, SAS, and SIB at random to balance the sample size of the reference populations. Parameters were set to the default in our analyses, and all the SNVs identified among XJU and the reference populations were used.

The local ancestry proportions were then averaged using the site-specific-ancestry within the sliding windows of 50 Kb in length shift by 25 Kb across the genome for each haplotype. The boundary between every two neighboring variants was firstly determined, so that the ratios of distance to global ancestry proportion of the corresponding ancestry were the same on both sides of the boundary. The ancestry proportion within each sliding window was estimated as proportion of the cumulative segment length for each ancestry. For the 2-reference analysis, the local ancestry proportions were around 0.5 for both ancestries across the genome (Fig. S6). There were about half of windows had EAS ancestry proportions ranging from 0.48 to 0.55, and ~95% ranging from 0.41 to 0.60, with the extremum reaching 0.30 and 0.70. Sliding windows were then ranked based on either the EAS or EUR ancestry proportions, and those with more extreme ancestry proportions had smaller ranks. The empirical *P*-value for each window was then calculated as the percentage of windows with smaller ranks.

Text S15. Post-admixture genetic diversity of XJU

We analyzed the genetic diversity of XJU as well the reference populations to understand the genetic diversity of the admixed population. Estimators including the nucleotide diversity (θ_π), haplotype diversity (H), normalized numbers of segregating sites (θ_K), numbers of singletons, proportions of rare SNVs (AF <0.05), and Tajima's D statistics (41) were employed.

Nucleotide diversity (θ_π) is measured as the average number of nucleotide differences per site between all pairs of DNA sequences within population (42). It is defined as

$$\theta_\pi = \sum_{ij} x_i x_j \pi_{ij},$$

in which x_i is the frequency of the ith sequence in the proportion and π_{ij} is the number of

nucleotide differences per nucleotide site between *i*th and *j*th sequence. Haplotype diversity measures the uniqueness of a particular haplotype in a given population (43). It is calculated as

$$H = \frac{N}{N-1}(1-\sum_i x_i^2),$$

where N is the number of haplotypes and x_i is the sample frequency of the *i*th haplotype, which is analogous to the heterozygosity at a single locus. The number of segregating sites (K) is equal to the number of mutations since the most recent common ancestor of the sequence in the sample, and it was normalized as

$$\theta_K = \frac{K}{\sum_2^n \frac{1}{n-1}}.$$

Estimators of genetic diversity including θ_π, H, θ_K, and Tajima's D were estimated within sliding windows of 50 Kb in length shift by 25 Kb increment, while proportions of rare SNVs were estimated across the whole genome. We random sampled 50 samples from each population to balance the sample size, and 100 replicates were conducted for the estimation of rare SNV proportions. Comparisons among different populations were conducted based on the Wilcoxon rank sum test. It was found that XJU had higher genetic diversity than the reference populations (Fig. S8). Genetic diversity estimated by θ_π and H were higher in XJU. And a higher proportion of rare SNVs was also found in XJU, indicating the contribution of the ancestry-specific SNVs from divergent ancestral populations and also the important role of the rare SNVs in XJU. This observation was also reflected by the larger value of θ_K than that of θ_π in XJU based on the negative-skew distribution of Tajima's D values. The same patterns were also observed for another admixed population, African American (Fig. S10).

To further investigate the effect of admixture, we analyzed the genetic diversity of XJU under different admixture proportions. Only haplotypes of homogenous ancestry within each sliding window were left, and windows including >80 EAS-ancestry-specific haplotypes and >80 EUR-ancestry-specific haplotypes were remained for the downstream analyses. Genetic diversity was then estimated within sliding windows using 80 random sampled haplotypes under different ancestry proportions, with EAS ancestry ranging from

0% to 100% advanced by 5% (corresponding to 4 haplotypes). Five replicates were conducted for each estimate, and the median value was finally employed. We then inferred the association between admixture proportion and genetic diversity based on the polynomial regression using R with command "lm(Y ~ poly(X, 2))", in which the independent variable X denotes admixture proportion and the dependent variable Y is the median value of genetic diversity across the genome. It was found that haplotypes with a substantial proportion of both ancestries had the highest genetic diversity, while those mainly contributed by either of the EAS ancestry or EUR ancestry had relatively lower genetic diversity (Fig. S11). The highest genetic diversity estimated by nucleotide diversity, haplotype diversity, numbers of segregating sites, numbers of singletons, and proportions of rare SNVs were achieved with EAS ancestry around 25%, 40%, 40%, 60%, and 90% (Fig. 3, Fig. S11), respectively. Besides, genetic diversity of ancestry-specific haplotypes was higher than that of the corresponding reference populations.

Genetic diversity was also estimated for regional XJU groups using the microarray data. Ten individuals were sampled at random without replacement from each regional group to balance the sample size. The median value of genetic diversity estimated within sliding windows across the genome was employed as the point estimate for each regional XJU group. Significant correlation between geographic coordinate and genetic diversity was also observed, while the genetic diversity of XJU groups residing in east was lower than that in west (Fig. S12), suggesting the admixture driven genetic diversity of XJU groups.

Text S16. Effective population size of XJU

We calculated the linkage disequilibrium (LD) for XJU, HAN, EAS, EUR, SAS, SIB, and AFR, using their merged data set together with the microarray data of XJU and HumanOrigins, including totally 426,722 SNVs. LD was estimated as the squared correlation (r^2_{LD}) for each population using vcftools (44) with command "--maf 0.05 --hap-r2". Only pairs of variants separated by genetic distances ranging from 0.01 to 0.25 centi-morgans (cM) were remained, leaving about 11 million pairwise LD observations. Then all of the r^2_{LD} values were grouped into 240 genetic distance categories of size 0.001 cM. The effective population size (N_e) over history were calculated based on the following relationship,

$$E(r_{LD}^2) = \frac{1}{2 + 4 \times N_e \cdot c},$$

in which c was the genetic distance between loci in morgans and $E(r_{\mathrm{LD}}^2)$ was estimated by averaging the r_{LD}^2 values within the given genetic distance bin (45). The long-term N_e was further calculated as the harmonic mean over all genetic distance categories.

We random sampled 50 samples from each population to balance the sample size. Relatively weaker LD and larger N_e of XJU were observed compared with other reference populations (Fig. S9), consistent with the large genetic diversity estimated in XJU. We also analyzed the regional XJU groups in the same way, while 10 individuals were random sampled from each group. Relatively stronger LD as well lower N_e were identified for XJU groups residing in the east compared with those in the west (Fig. S12), supporting the admixture-driven genetic diversity of XJU groups.

Text S17. Genetic burden

Analysis of genetic burden was performed by comparing the individual accumulation of deleterious SNVs among XJU, EAS, and EUR. The deleterious SNVs were defined based on the variant consequence impact. Both the loss-of-function (LoF) SNVs (high impact) and missense SNVs (moderate impact) were considered as deleterious. Genetic burden for each individual was measured as the total number of deleterious SNVs across the genome under the additive (# heterozygous + 2×homozygous loci), dominant (# heterozygous + homozygous loci), and recessive (# 2×homozygous loci) models. Comparisons of genetic burden between XJU and reference populations were conducted by Wilcoxon rank sum test. The results showed that XJU individuals had the most deleterious SNVs compared with other reference populations when considering the dominant model, followed by EAS individuals. But XJU individuals had the number of total deleterious alleles and homozygotes between EAS and EUR under the additive and recessive models, while EAS individuals had the most deleterious alleles and homozygotes (Fig. S13). Population-specific demography may interpret the varying deleterious homozygotes and heterozygotes across populations (46), the higher number of deleterious homozygotes in EAS is largely due to the increasing number of fixed or nearly fixed variants, and the highest number of deleterious heterozygotes found in XJU individuals should be attributable to their large

number of rare SNVs introduced by population admixture (Fig. S4). The same pattern was also observed when the numbers of deleterious SNVs were weighted by the Combined Annotation–Dependent Depletion (CADD) score (12) (Fig. S13). Further, association analyses revealed the significant correlation between individual ancestry proportion and genetic burden under both additive and recessive models, while higher EAS ancestry may indicate more deleterious SNVs for XJU individuals.

In addition to the mutation consequence impact, another 2 estimators were also employed to represent different degrees of mutational effects, including CADD score and Genomic Evolutionary Rate Profiling (GERP) Rejected Substitution (RS) score (13). The annotation source of CADD score was obtained from http://cadd.gs.washington.edu/download, with larger CADD scores implying the more deleterious state. And the annotation source of GERP score was accessed from the UCSC genome browser (http://hgdownload.cse.ucsc.edu/gbdb/hg19/bbi/All_hg19_RS.bw). GERP score was an estimator of the conservation across a phylogeny, and a large positive GERP score reflect the high degree of conservation. To eliminate the potential reference bias in assessing levels of conservation (46), reference-derived sites were removed from our analyses, as well as those with ambiguous derived alleles. All the variants were grouped into bins with CADD score ranges (min, 15), [15, 25), and [25, max), representing the neutral, moderate, and large biological impact, respectively. We then measured the genetic burden for XJU individuals as the total number of derived deleterious SNVs as well that weighted by the CADD score within each CADD score bin under the additive, dominant, and recessive models. For the analyses of GERP score, all the SNVs were sorted into 3 bins, also reflecting the different mutational effects, with ranges (min, 2), [2, 4), and [4, max). We estimated the genetic burden of each individual in the same way. Comparisons of the genetic burden indicated by CADD and GERP scores among XJU, EAS, and EUR also revealed the highest genetic burden of XJU under the dominant model (Fig. S13). And we also observed the positive correlation between the admixture proportion and genetic burden of XJU individuals, confirming the admixture-driven genetic burden.

Further, we investigated genetic burden for all the KEGG pathways (47) and genes across the genome. It was revealed that XJU individuals had either higher or similar genetic burden compared with EAS and EUR individuals across those reginal components under

the dominant model. There were 31 pathways (8.2%) and 14 genes (0.1%) having significantly more deleterious SNVs in XJU individuals (BH-corrected P <0.05, Wilcoxon rank sum test), including pathways related to metabolism, digestion, and immunity (Table S4–S5, Fig. S14). We also identified 57 pathways and 3 genes with the highest genetic burden in XJU individuals based on the estimator of CADD, while there were 130 pathways and 56 genes using the estimator of GERP. Pathways related to metabolism, digestion, and immunity were found in all of these analyses. Besides, pathways related to the sensory system were identified based on the estimator of GERP score, including the pathway related to taste transduction.

Text S18. Modified Gene Set Enrichment Analysis

Gene Set Enrichment Analysis (GSEA) is a statistical method developed based on the Kolmogorove-Smirnov test to identify the enrichment of biologically functional categories in a ranked gene list (48). It is different from the hypergeometric method. GSEA employs the genome-wide genes and is independent of any threshold, but gene ranks are required. It would help to characterize the genome-wide AFd_e of coding genes in XJU, especially those sharing the similar biological functions. However, genes were not strictly ranked according to their quantiles of AFd_e in our analysis, since the limiting and unequal number of windows across SNV density bins would result in different ranges of the empirical P-values.

We further modified the traditional GSEA method (mGSEA). All genes were sorted by their AFd_e quantiles into bins of size 0.005, and we got totally 200 bins in rank. For a given gene set S (a pathway or a priori functional gene set) and a AFd_e quantile bin B, we defined the "hit" score $P_{hit}(S,B)$ and "miss" score $P_{miss}(S,B)$ as

$$P_{hit}(S,B) = \frac{n_S}{N};\; P_{miss}(S,B) = \frac{N - n_S}{N},$$

in which the n_s stood for the number of genes in B belonging to S, and N was the number of genes in B. So that we defined the net score $P_{net}(S,B)$ for a given bin as

$$P_{net}(S,B) = P_{hit}(S,B) - P_{miss}(S,B).$$

The cumulative score $P_{net}(S)$ was calculated as

$$P_{net}(S) = \sum_{i=1}^{n} P_{net}(S, B_i)$$
,

in which B_i was the ith bin in the ranking list. Further, the enrichment score *ES(S)* was defined as the maximum deviation of $P_{net}(S)$ from zero. The significance of an observed *ES(S)* was estimated by comparing it with the NULL distribution of *ES* (ES_{NULL}), which was generated by permuting the gene-pathway relationship 20,000 times. Finally, the nominal empirical *P*-value for the gene set *S* was calculated by the one-tail significance test using either the positive or negative portion of the NULL distribution according to the sign of the observed *ES(S)*.

Genome-wide genes were ranked according to their AFd_e quantiles in either 2-reference or 4-reference analysis. We then used the mGSEA method to investigate the AFd_e of pathway genes. There identified totally 161 pathways enriched for genes with high AFd_e accumulation based on the gene list ranked in 2-reference analysis (Table S6). And 99 out of the 161 pathways (61.5%) were related to "Organismal Systems" and "Human Diseases", including the pathways related to immunity, digestion, and nervous system. Another 22 pathways (13.7%) were related to metabolism (e.g., carbohydrate metabolism and lipid metabolism) (Fig. S15). Notably, it was interesting to find the under-representation of genes with high AFd_e accumulation for pathways related to olfactory transduction, which may be associated with the conservation of most olfactory receptor genes in XJU. For the ranked gene list from 4-reference analysis, we identified 165 pathways enriched for genes with high AFd_e accumulation (Table S6). And 114 out of the 165 pathways were related to "Organismal Systems" and "Human Diseases" (69.1%), while another 13 were related to metabolism (7.9%).

Text S19. Within-population signatures of selective sweep (iHS)

Haplotype-homozygosity-based method iHS (49) was applied to detect the signatures of putative selective sweeps in XJU as well the reference populations. Software Selscan (v1.1.0a) was used for the genome-wide scan with all parameters set to the default. We calculated the proportions of SNVs with |iHS| score >2 for sliding windows of 50 Kb in length shift by 25 Kb across the genome. To eliminate the potential bias caused by the varying variant densities across sliding windows, we grouped all sliding windows by the

variant counts into bins of size 10. Windows with >180 SNVs were grouped together, while those with <10 SNVs were dropped, leaving totally 101,132 windows. Next, windows were ranked within the corresponding bin by their proportions of SNVs with |iHS| score >2, and the empirical *P*-value for each window was calculated as the percentage of windows with higher ranks. We picked out windows with empirical *P*-value <0.5% as candidates of selective sweeps.

There were totally 192 regions identified in XJU, 241 in EAS, and 235 in EUR, while 39 and 31 regions respectively in EAS and EUR were also found in XJU, encompassing genes such as *PDE11A* (2q31.2) and *ATP6V1E1* (22q11.21) in EAS and *EXOC6B* (2p13.2) and *SLC24A5–SLC12A1* (15q21.1) in EUR. *PDE11A* and *ATP6V1E1* were respectively related to the nucleotide metabolism and energy metabolism, while *EXOC6B* was associated with the blood pressure. Population-specific signatures of selective sweep in XJU included *COL11A1* (1p21.1) and *ICA1* (7p21.3), which were respectively related to the "Protein digestion and absorption" and "Type I diabetes mellitus" pathways. *COL11A1* encodes components of type XI collagen, which could provide structure and strength to muscle and skin. Higher allele sharing was observed between XJU and EAS at the missense variant rs11164663, which had DAF of 0.016, 0.034, and 0.101 in XJU, EAS, and EUR. The haplotype carrying the DA were almost lost in XJU. Meanwhile, we inferred lower expression level of *COL11A1* in XJU and EAS, as it was reported that low DAF at rs11164663 may down-regulate the expression of *COL11A1* (50).

We found the highest proportions of iHS signals on chromosome 4 (1.37%), 17 (1.08%), and 20 (1.03%) in XJU, while it was 0.62% for the genome-wide mean (Fig. S19.1). Meanwhile, the lowest proportions of iHS signals were found on chromosome 1 (0.23%), 9 (0.32%), and 13 (0.11%) in XJU. We partitioned the genome into sliding windows of 5 Mb in length shift by 1 Mb increment, and analyzed the local enrichment of the iHS signals within each sliding window. The local enrichment of iHS signals was analyzed by calculating the numbers of segments with significant iHS signals within each sliding window, assuming the Poisson distribution across the chromosome. We picked out windows with BH-corrected *P*-value <0.001 as candidates, obtaining 81 sliding windows with significant signals in XJU (Fig. S19.1), while the most significant signal was found on chromosome 4, encompassing lincRNAs including *RP11-79E3.3* and *RP11-548L20.1*

(4p15.1). There also identified signals ranking the top on chromosome 3 and 6, respectively encompassing *RPL15* (3p24.2) and *TRMT11* (6q22.32). *RPL15* was also identified as a selective sweep in EUR, encoding a ribosomal protein.

Notably, it was interesting to find that *LIMS1* (2q12.3) and *SLC24A5–SLC12A1* (15q21.1) were also identified as selective sweeps in XJU. *LIMS1* is associated with eyebrow thickness in both Han Chinese and Uyghur (51) at the intron variant rs1866188, which is ~200 Kb downstream to *EDAR*. The DAF of rs1866188 is 0.389 in XJU, 0.927 in EAS, and 0 in EUR, suggesting the unbiased genetic makeups of XJU post admixture. Signature of selection was observed in both XJU and EAS on *LIMS1*. All of the SNVs on the gene region of *LIMS1* had |iHS|>2 in XJU. Meanwhile, we observed long extended haplotype homozygosity (EHH) on *LIMS1* (Fig. S24) as well lower genetic diversity in XJU compared with EUR ($P < 2.2\times10^{-16}$, Wilcoxon rank sum test) (Fig. S25). Although *LIMS1* is in linkage with *EDAR* (r^2=0.71), no signal of selective sweep was observed in XJU on *EDAR*, and the genetic diversity estimated on *EDAR* was the highest in XJU compared with the reference populations ($P < 2.2\times10^{-16}$, Wilcoxon rank sum test) (Fig. S25).

SLC24A5–SLC12A1 is associated with the skin pigmentation (52-54). The similar pattern was observed on the *SLC24A5–SLC12A1* region. Frequency profiles on *SLC24A5–SLC12A1* also suggest the unbiased ancestry makeup of XJU post admixture. The DAF of key variant rs1426654 on *SLC24A5* is 0.587, 0.029, and 1 in XJU, EAS, and EUR, respectively. And DAF of rs11636073 on *SLC12A1* was 0.620 in XJU, 0.184 in EAS, and 0.919 in EUR. Selective sweeps on the *SLC24A5–SLC12A1* region were identified in both XJU and EUR. Lower genetic diversity was observed in XJU compared with EAS. About 80–90% SNVs on the *SLC24A5–SLC12A1* region had |iHS|>2 in XJU. And long EHH was also observed, indicating the long haplotypes introduced by admixture and limited recombination events post admixture.

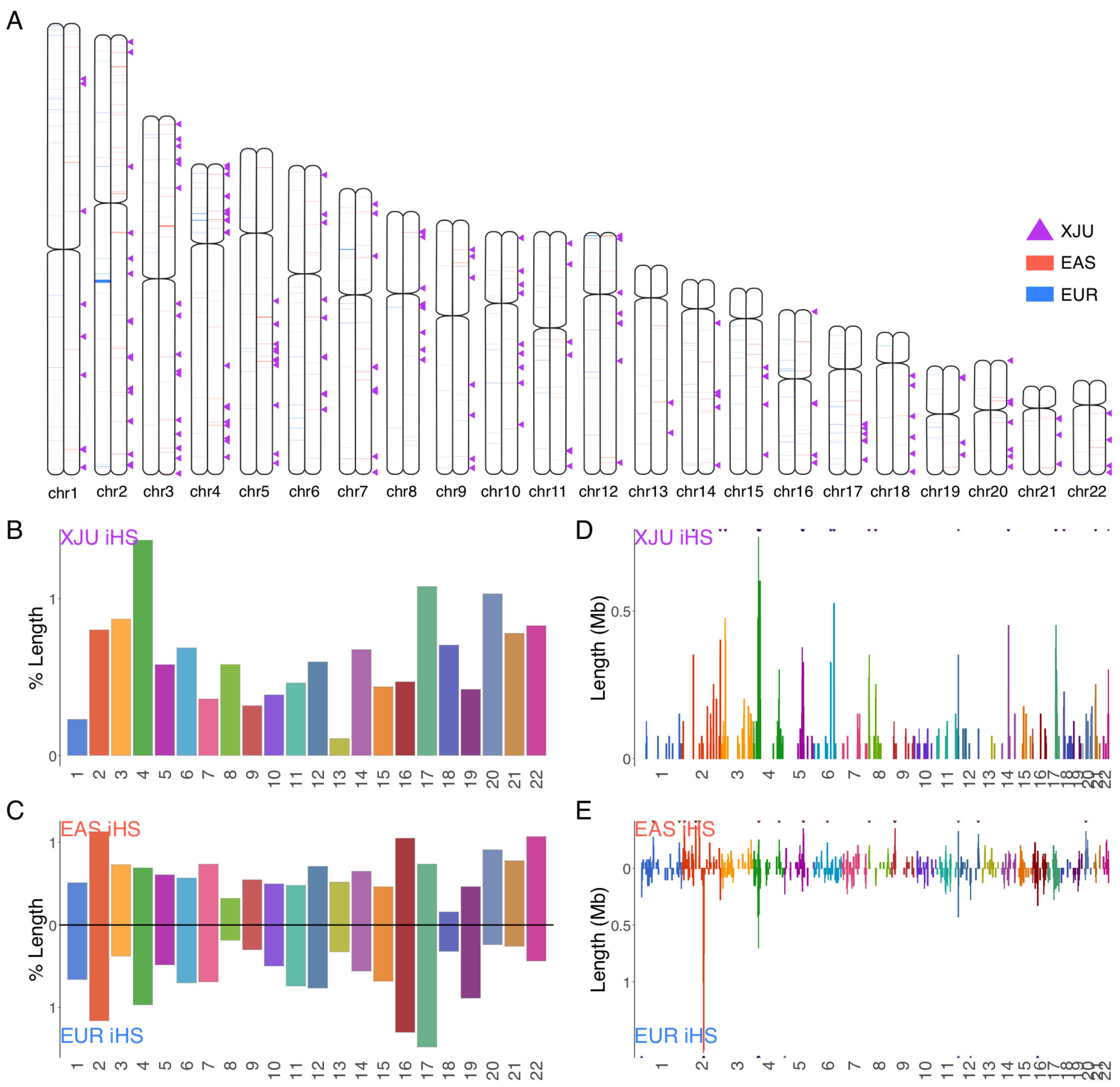

Figure S19.1 | Selective sweeps indicated by the iHS signals.

A. Chromosome plot for signatures of selective sweep indicated by iHS. Visualization was achieved by tagore (55); **B.** Proportions of the cumulative length of segments identified as selective sweeps in XJU across chromosomes; **C.** Proportions of the cumulative length of segments identified as selective sweeps in EAS and EUR across chromosomes; **D.** Cumulative lengths of segments identified as selective sweeps within sliding windows of 5 Mb in length shift by 1 Mb across the genome of XJU. Windows with *P* <0.001 (Poisson test on each chromosome, BH-corrected) were indicated by triangles on the top; **E.** Cumulative lengths of segments identified as selective sweeps within sliding windows across the genomes of EAS and EUR.

Text S20. Within-population signatures of selective sweep (H12 + G12)

The H12 (56) and G12 (57) statistics were also applied to investigate the selective sweeps for XJU, since the two methods were reported to be robust to the admixture effect and were powerful in detecting soft sweeps. The H12 is a haplotype-homozygosity-based method,

while the G12 method was based on unphased multi-locus genotypes. Following the procedures processed in the original papers, both H12 and G12 statistics were calculated within the sliding windows of 40Kb by increment of 20Kb. All of the sliding windows were ranked by either G12 or H12 (G12/H12) values. To control the influence of the variant density, we grouped all windows by their SNV counts into bins of size 10. Windows of >300 SNVs were grouped together, as well as those of <120, while those containing <40 SNVs were dropped. The empirical *P*-value for each window was then calculated as the percentage of windows with larger G12/H12 values in the same bin.

We picked out windows as candidates with G12/H12 values ranking the top 1% both in the corresponding bins and across the whole genome, obtaining totally 317 regions with significant G12 values and 309 with significant H12 values, with ~70% sharing signals. We got totally 407 segments by merging the regions identified by H12 and G12 statistics. And there were 558 regions identified in EAS and 497 in EUR, while 31 regions were shared among all of the 3 populations. Totally 247 regions were specifically identified in XJU, encompassing genes including *COL11A1* (1p21.1) and *CBR4* (4q32.3), which were respectively related to “Protein digestion and absorption” and “Lipid metabolism”, while *COL11A1* was also identified with iHS signal in XJU. Sharing signals were found between XJU and EAS on genes including *PGA3* (11q12.2) and *FADS1–FADS2* (11q12.2). *PGA3* is related to the pathway “Protein digestion and absorption” and *FADS1–FADS2* is related to fatty acid desaturase. Meanwhile, positive selection of *FADS1–FADS2* was also reported in SAS, AFR, and EAS populations (58). Signals on *GRM5* (11q14.3) and *KRTAP21* (21q22.11) were shared between XJU and EUR, while *GRM5* is associated with skin pigmentation and *KRTAP21* is related to the keratinization. Notably, we also identified *LIMS1* (2q12.3) and *SLC24A5–SLC12A1* (15q21.1) as selective sweeps in XJU, indicating the sharing signals between G12/H12 and iHS. G12/H12 signal was also found on *SULT1C4*, which is ~150 Kb upstream to *LIMS1*. And the variant rs4149433 on *SULT1C4* is associated with facial morphology, including lobe attachment (59) and ear protrusion (60).

We found the highest proportions of G12/H12 signals on chromosome 2 (1.09%) and 17 (1.11%) in XJU, while it was 0.72% estimated as the genome-wide mean (Fig. S20.1). The lowest proportions of G12/H12 signals were on chromosome 21 (0.29%) and 22

(0.19%) in XJU. Regional enrichment analysis identified 59 sliding windows in XJU (Fig. S20.1), with the most significant signal found on chromosome 1, encompassing *PKN2* (1p22.2) and *EVI5* (1p22.1), which are respectively related to protein kinase activity and GTPase activator activity. G12/H12 signals of *PKN2* were also observed in EUR, while the signal of *EVI5* were also found in EAS.

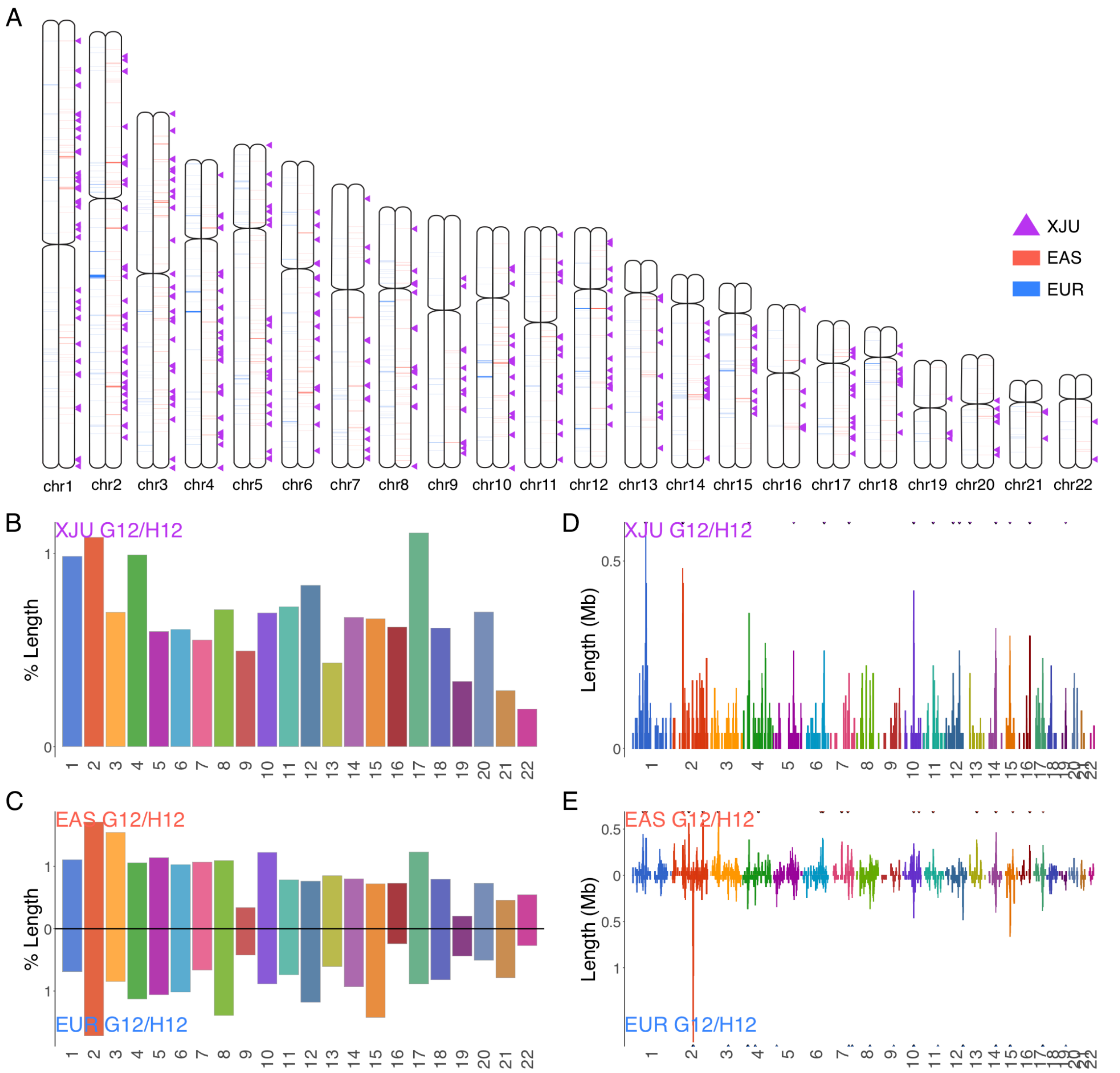

Figure S20.1 | Selective sweeps indicated by the G12/H12 signals.

A. Chromosome plot for signatures of selective sweep indicated by G12/H12; **B.** Proportions of the cumulative length of segments identified as selective sweeps in XJU across chromosomes; **C.** Proportions of the cumulative length of segments identified as selective sweeps in EAS and EUR across chromosomes; **D.** Cumulative lengths of segments identified as selective sweeps within sliding windows of 5 Mb in length shift by 1 Mb across the genome of XJU. Windows with *P*

<0.001 (Poisson test on each chromosome, BH-corrected) were indicated by triangles on the top; **E.** Cumulative lengths of segments identified as selective sweeps within sliding windows across the genomes of EAS and EUR.

Text S21. Cross-population signatures of selective sweep

The XP-EHH (61) method was applied to investigate the cross-population signatures of selective sweeps between XJU and the reference populations. We used Selscan (v1.1.0a) with all parameters set as default. We calculated the proportions of SNVs with |XP-EHH| score >2 for the sliding windows of 50 Kb in length shift by 25 Kb across the genome. We then grouped all of the windows by variant counts into bins of size 10. Windows with <170 SNVs were grouped together, as well those with >430 SNVs, while windows with <10 SNVs were dropped. There remained totally 103,632 windows. We ranked sliding windows within the corresponding bins according to their proportions of SNVs with |XP-EHH| score >2. The empirical *P*-value for each window was estimated within the corresponding bin as percentage of windows with higher ranks. Windows with empirical *P*-value <0.5% were picked as the candidates.

There identified totally 266 regions between XJU and EAS ($XP_{XJU\text{-}EAS}$), and 282 between XJU and EUR ($XP_{XJU\text{-}EUR}$). The genome-wide mean proportions of both $XP_{XJU\text{-}EAS}$ and $XP_{XJU\text{-}EUR}$ components were 0.7%. We found the highest proportions of $XP_{XJU\text{-}EAS}$ on chromosome 2 (1.61%), 3 (1.15%), and 10 (1.22%), while the lowest were found on chromosome 18 (0%) and 19 (0.08%) (Fig. S21.1). The highest proportions of $XP_{XJU\text{-}EUR}$ signals were on chromosome 2 (1.46%), 12 (0.99%), and 15 (1.68%), while the lowest were on chromosome 16 (0.22%), 17 (0.31%), and 20 (0.12%). The highest proportions of $XP_{XJU\text{-}EAS}$ and $XP_{XJU\text{-}EUR}$ components identified on chromosome 2 were related to regions encompassing *EDAR* (2q12.3) and *LCT–MCM6* (2q21.3), respectively. And the XPEHH scores on both regions indicated the selective sweeps in the reference populations. Moreover, signals of XPEHH between XJU and EUR were also observed on some well-known genes including *SLC45A2* (5p13.2), *BNC2* (9p22.2), and *HERC2–OCA2* (15q13.1), while all of these signals indicated the selective sweeps in EUR.

Signals of selective sweep in XJU were observed among 60 $XP_{XJU\text{-}EAS}$ components (22.56%) and 75 $XP_{XJU\text{-}EUR}$ components (26.60%). For the $XP_{XJU\text{-}EAS}$ components, there identified genes including *PNLIPRP3* (10q25.3) and *LRP1* (12q13.3), which were

respectively related to lipid metabolism and cholesterol metabolism. And genes including *COL11A1* (1p21.1) and *PGA3* (11q12.2) were found as the $XP_{XJU\text{-}CEU}$ components, while both genes were related to protein digestion and absorption. Meanwhile, gene *COL11A1* was identified by iHS in XJU, and *PGA3* was identified by G12/H12 statistics in XJU. There shared 9 regions between $XP_{XJU\text{-}EAS}$ and $XP_{XJU\text{-}EUR}$, including the region encompassing *SLC24A5–SLC12A1*, which was also identified by iHS in XJU. The scenario would be relatively complex for the *SLC24A5–SLC12A1* region, since signal of selective sweep was observed in EUR when comparing XJU and EUR, and it was in XJU when comparing XJU and EAS.

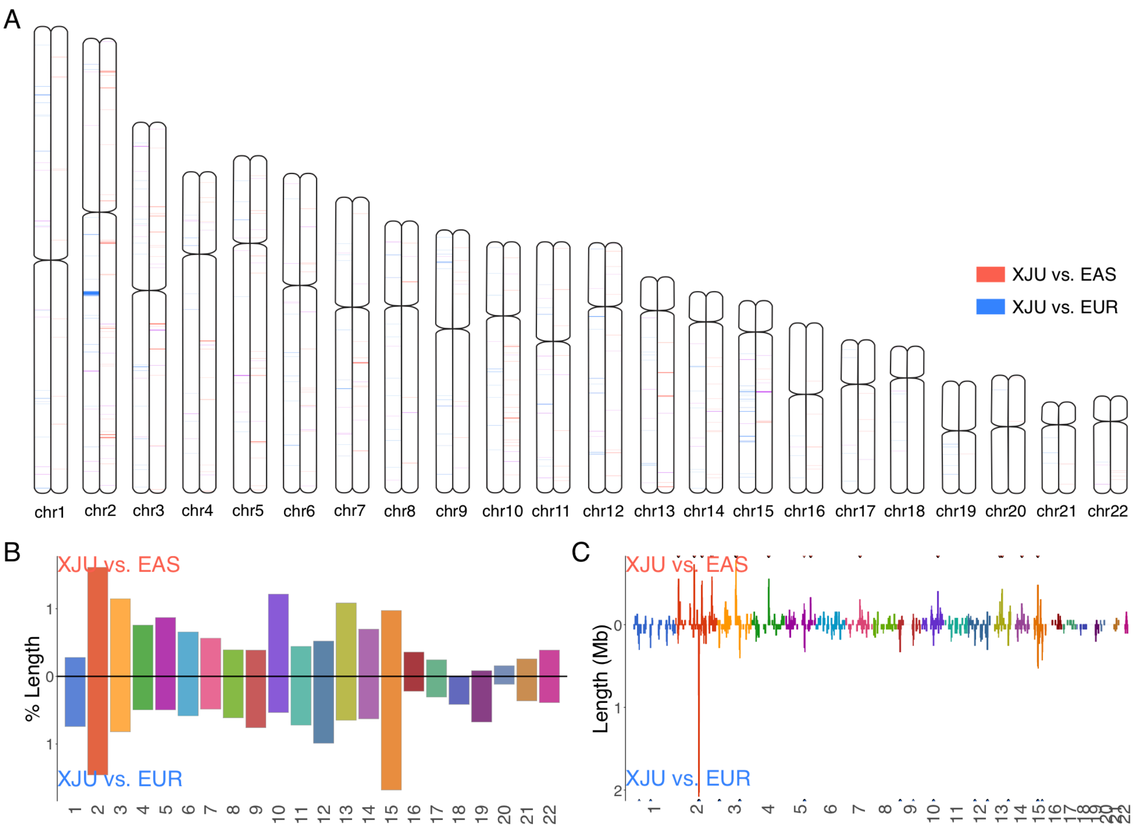

Figure S21.1 | Selective sweeps indicated by the XPEHH signals.

A. Chromosome plot for signatures of selective sweep indicated by XPEHH; **B.** Proportions of the cumulative length of segments identified as selective sweeps across chromosomes; **C.** Cumulative lengths of segments identified as selective sweeps within sliding windows of 5 Mb in length shift by 1 Mb across the genome. Windows with *P* <0.001 (Poisson test on each chromosome, BH-corrected) were indicated by triangles.

Text S22. Admixture-representative genetic components

Admixture-representative (AR) genetic components were defined as the segments that contributed equally by the highly divergent ancestries. Population branch statistics (PBS) (62) was applied to quantify the local population admixture. PBS was calculated based on the frequency differentiation (F_{ST}) and was formally used as an indicator of the selective sweep. Strong positive selections would result in the long population-specific branches as well extremely positive PBS values. Due to the sharing genetic components between the admixed population and ancestral populations, population-specific branch length of the admixed population would be negative. We evaluated the relationship between PBS values and admixture proportions by simulate both ancestral and admixed populations. To simplify the analysis, we constrained the allele frequencies in the ancestral populations as f_1 = 100% - f_2, where f_1 and f_2 respectively denote the frequencies of the two ancestral populations and f_1 <0.5. The frequencies of the admixed population (f_{adm}) range from f_1 to 0.5. We calculated the admixture proportion (m) as m=(f_{adm}- f_1)/(f_2- f_1). The results revealed that increasing admixture proportion and frequency difference between ancestral populations would result in lower PBS values (Fig. S22.1). So that PBS could be principally considered as an estimator for the local population admixture.

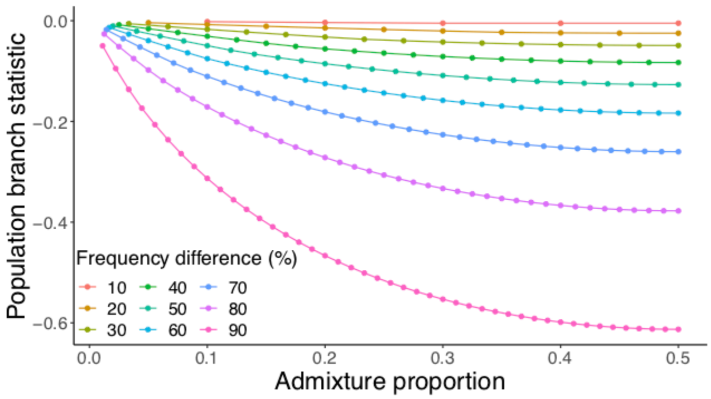

Figure S22.1 | Evaluation of the population branch statistic (PBS) as an estimator of population admixture.

We calculated PBS for XJU, with EAS and EUR assigned as reference populations. For each SNV and local segment that identified in all 3 populations, PBS value was calculated as

$$PBS = \frac{T_{XJU-EAS} + T_{XJU-EUR} - T_{EAS-EUR}}{2},$$

in which the T statistics was a measurement of the divergence between each pair of populations and was calculated as

$$T = -\log(1 - F_{ST}).$$

Meanwhile, SNVs with AF <0.01 or >0.99 in all of the 3 populations were removed. It was observed that the genome-wide distribution of the PBS values was negative-skewed, consistent with that of expected. There were 5,568,357 SNVs (69.2%) having negative PBS values across the genome of XJU. There were 3 most significant segments that harbor 34 SNVs with PBS values <-0.5 (Table S7), and they were at 2q12.3 (encompassing 6 genes: *SULT1C4*, *GCC2*, *LIMS1*, *RANBP2*, *CCDC138*, and *EDAR*), 5p13.2 (encompassing gene *SLC45A2*), and 15q21.1 (encompassing 3 genes: *SLC24A5*, *CTXN2*, and *SLC12A1*). The frequencies of the 34 variants were highly differentiated between EAS and EUR with one allele almost fixed in EAS and the other in EUR, but middle in XJU. And 3 out of the 34 SNVs were missense mutants, including rs3827760 (*EDAR*), rs16891982 (*SLC45A2*), and rs1426654 (*SLC24A5*). The *EDAR*-V370A mutation at rs3827760 was associated with the hair thickness (63, 64), ear lobe and chin morphology (65), and incisor morphology (65-68) in East Asian populations, as well as the sweet gland (69, 70). Its DAF was 0.418 in XJU, 0.937 in EAS, and 0 in EUR, implying the balanced ancestry makeups in XJU. The other two variants on *SLC45A2* and *SLC24A5* were related to the skin, hair, and eye colors in European populations (52-54, 71-74). The DAF was 0.228 in XJU, 0.015 in EAS, and 0.980 in EUR at rs16891982. And they were 0.587, 0.029, 1.0 in XJU, EAS, and EUR at rs1426654, respectively.

PBS values were further calculated for sliding windows of 50 Kb in length shift by 25 Kb across the genome, using the regional F_{ST} calculated within all of the sliding windows. The distribution of the PBS values was also negative-skewed, with 99,222 windows (93.6%) having negative PBS values. We kept local segments with PBS values <-0.1 (top 1%) as candidates of AR components, while all of them had $F_{ST[EAS-EUR]}$ values larger than 0.32. And regions with biased ancestry were further filtered, remaining totally 522 AR segments (Fig. S22.2). Besides *EDAR* and *SLC24A5*, there also identified other genes related to important biological functions, including *ADH1B* (4q23), *ALDH2* (12q24.12), and the

AGO gene cluster (1p34.3) (e.g., *AGO1*, *AGO3*, and *AGO4*). *ADH1B* and *ALDH2* might be responsible for the alcoholism protection in Asian populations (75, 76) and the *AGO* gene cluster is related to the RNA interference and silencing (77). Balanced ancestry makeup in XJU was confirmed by the frequency profiles. The *ADH1B**2 allele at rs1229984 had frequency of 0.375 in XJU, 0.709 in EAS, and 0.015 in EUR, and the allele frequencies were 0.038 in XJU, 0.160 in EAS, and 0 in EUR at rs671 on *ALHD2*. There were also AR components related to some fundamental biological functions, such as melanogenesis, UV-B response, neuron development, and metabolism, but without signature of enrichment.

We further looked into the physical distribution of the AR components, since the AR components were expected to be evenly distributed across the genome under the null hypothesis. Over-/under-representation of AR components were found on different chromosomes. The highest proportions of AR component were identified on chromosome 1 (1.91%), 2 (2.16%), and 12 (2.13%), and the lowest were on chromosome 7 (0.82%), 18 (0.32%), and 22 (0.78%), while the mean proportion of AR components was 1.35% across the genome. Regional enrichment analysis was conducted by partitioning the whole genome into sliding windows of 5 Mb in length shift by 1 Mb and assuming that the count of AR regions within each sliding window follows the Poisson distribution. There identified totally 158 sliding windows with significant signals for enriching AR components. The most significant signals were found on chromosome 1, 2, 6, and 12 (BH-corrected P =0), while regions encompassing the *AGO* gene cluster contributed to the signature on chromosome 1.

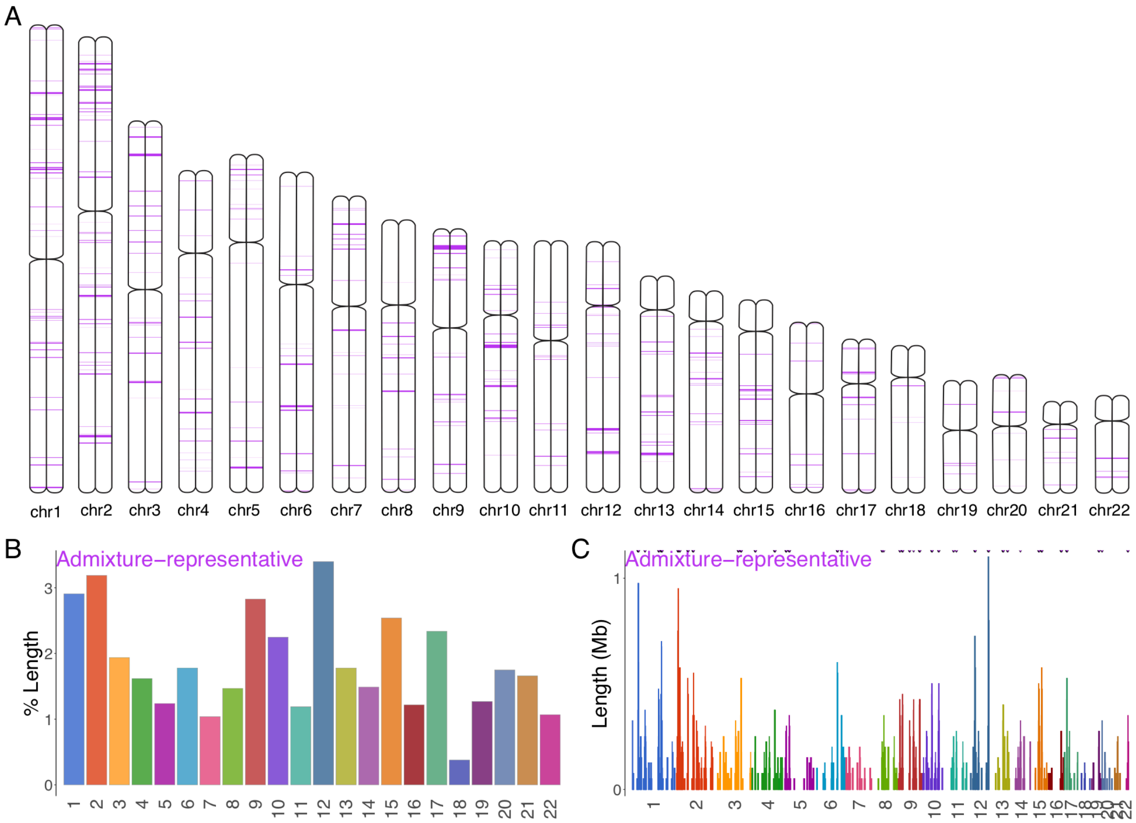

Figure S22.2 | Distribution of the admixture-representative (AR) components.
A. Chromosome plot for AR components identified in XJU; **B.** Proportions of the cumulative length of AR components across chromosomes; **C.** Cumulative lengths of AR components within sliding windows of 5 Mb in length shift by 1 Mb across the genome. Windows with P <0.001 (Poisson test on each chromosome, BH-corrected) were indicated by triangles on the top.

Text S23. Reconstructed ancestral populations

Based on the inferred locus-specific ancestry, we partitioned the genomes of XJU to reconstruct the ancestral populations. Comparison of the genomes between reconstructed ancestral population and reference populations may help reflect the local adaption of the ancestral genetic component in XJU post admixture. We calculated AF for both the reconstructed ancestral population (AF_{anc}) and reference population (AF_{ref}), then AFd_e was calculated as $|AF_{anc} - AF_{ref}|$. SNVs with AF <0.01 or >0.99 in all of XJU, EAS, and EUR were dropped, leaving totally 9,004,531 SNVs. The significances of AFd_e were further estimated based on their ranks. To eliminate the potential influence of MAF, we grouped all SNVs across the genome according to their MAF in the reconstructed ancestral population (MAF_{anc}) into bins of size 0.01. Then the empirical P-value for each SNV was estimated within the corresponding bin as the percentage of SNVs with larger AFd_e value.

We grouped all the SNVs according to their empirical *P*-values into different bins. And significantly larger AFd_e values were observed among SNVs ranking the top 1% ($P_{top\ 1\%\ vs.\ top\ 2–3\%}$ <2.2×10^{-16}, Wilcoxon rank sum test) (Fig. S23.1). We took empirical *P*-value <0.01 as the threshold to identify SNVs of large AFd_e. There were 90,020 SNVs with empirical *P*-value <0.01 between EAS and the reconstructed ancestral EAS population (aEAS), while 90,020 SNVs were identified between EUR and the reconstructed ancestral EUR population (aEUR).

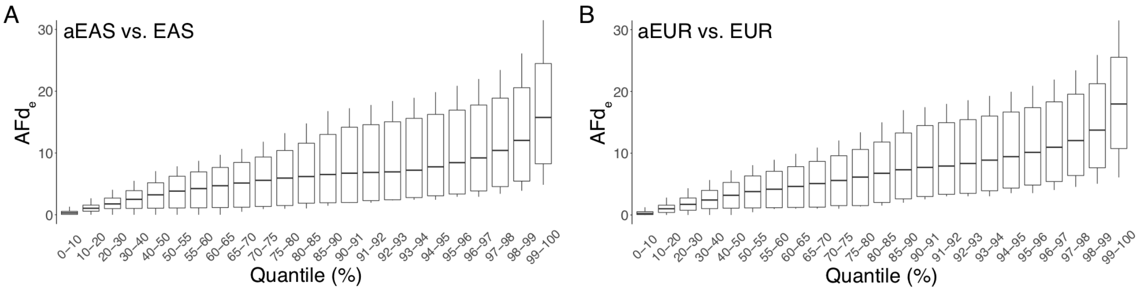

Figure S23.1 | Distribution of AFd_e across the quantile bins.
AFd_e and quantiles were estimated between either **A.** aEAS and EAS or **B.** aEUR and EUR. SNVs were grouped into bins of similar quantiles. Extreme shifts of AFd_e were observed in both analyses for SNVs ranking the top 1%.

Proportions of SNV with large AFd_e (empirical *P* <0.01) were calculated within sliding windows of 50 Kb in length shift by 25 Kb across the genome. Since the SNV density varied considerably across the sliding windows, we grouped all windows by the variant counts into bins of size 10. Windows with <60 SNVs were grouped together, as well those with >280 SNVs, while those with <50 variants were excluded from the further analyses, leaving 104,190 windows. Then the empirical *P*-value for each window was calculated within the corresponding bin as the percentage of windows with higher proportion of large AFd_e SNVs. Regions ranking the top 0.5% were selected as candidates representing the ancestry-biased (AB) components between the reconstructed ancestral population and reference population. We obtained 243 segments between aEAS and EAS (AB_{aEAS}), and 228 between aEUR and EUR (AB_{aEUR}).

Analyses of the physical distribution revealed the local enrichment of both AB_{aEAS} and AB_{aEUR} components. The mean proportion was 0.7% for both AB_{aEAS} and AB_{aEUR} components across the genome. For the AB_{aEAS} component, the highest proportions were

identified on chromosome 7 (1.48%), 10 (1.03%) and 12 (1.36%), while the lowest were on chromosome 18 (0.22%), 19 (0.25%) and 21 (0.16%) (Fig. S23.2). Meanwhile, the highest proportions for AB_{aEUR} components were on chromosome 8 (0.92%), 11 (1.46%), and 17 (1.05%), while the lowest were on chromosome 14 (0.37%), 18 (0.35%), and 21 (0.36%) (Fig. S23.2). Majority of the ancestry-biased components between reconstructed ancestral population and reference population identified on chromosome 7 and 12 were differentiated between aEAS and EAS, while those on chromosome 11 and 17 were differentiated between aEUR and EUR.

There were 44 sliding windows identified with regional enrichment for AB_{aEAS} components (Fig. S23.2). The most significant signal was observed on chromosome 12 (BH-corrected P =0), encompassing genes including *ALDH2* (12q24.12), which encode the aldehyde dehydrogenase family of proteins and is related to the major oxidative pathway of alcohol metabolism. Besides, we also found genes including *EDAR* (2q12.3), *HLA* (6p21.32), *CYP3A* (7q22.1), and *ARHGAP42* (11q22.1) among the AB_{aEAS} components. The *EDAR*-V370A mutation at rs3827760 was associated with the hair thickness (63, 64), ear lobe and chin morphology (65), and incisor morphology (65-68) in East Asian populations, as well as the sweet gland (69, 70). Allele frequency difference was observed between aEAS and EAS. The DAF was 0.418 in XJU, 0.937 in EAS, 0 in EUR, 0.605 in aEAS, and 0.272 in aEUR. Signal observed on *EDAR* may indicate the post-admixture recombination and selection relaxation in XJU. EUR-biased ancestry was observed on *ARHGAP42*. The DAF of rs7947761, which was reported to be associated with the coronary artery disease (78), was 0.745 in XJU, 0.985 in EAS, 0.768 in EUR, 0.909 in aEAS, and 0.626 in aEUR. For rs6590810 that associated with the hair color (79), XJU had the DAF equaling 0.582, and it was 0.456 in EAS, 0.495 in EUR, 0.494 in aEAS, and 0.645 in aEUR. In addition, there found 85 sliding windows with significant signals for their regional enrichment of AB_{aEUR} components (Fig. S23.2). The most significant signal was observed on chromosome 2 and 11 (BH-corrected P =0). The AB_{aEUR} components on chromosome 11 were enrichment for Olfactory Receptor genes (11q11–q12), and those on chromosome 2 encompassed genes including *TMEM163* and *LCT–MCM6* (2q21.3). EAS-biased ancestry was also found on both *TMEM163* and *MCM6–LCT*. The DAF of key variant rs4988235 on *MCM6–LCT* was 0.05 in XJU, 0.0 in EAS, 0.737 in

EUR, 0.036 in aEAS, and 0.081 in aEUR. For the key variant rs6739706 on *TMEM163*, its DAF was 0.766 in XJU, 0.825 in EAS, 0.364 in EUR, 0.792 in aEAS, and 0.731 in aEUR.

We did GO and KEGG enrichment analyses for AB_{aEAS} and AB_{aEUR} components (Table S9). The results revealed the enrichment of AB_{aEUR} components for OR genes (e.g., hsa04740: Olfactory transduction, GO:0050911: detection of chemical stimulus involved in sensory perception of smell, GO:0007608: sensory perception of smell, and GO:0050907: detection of chemical stimulus involved in sensory perception).

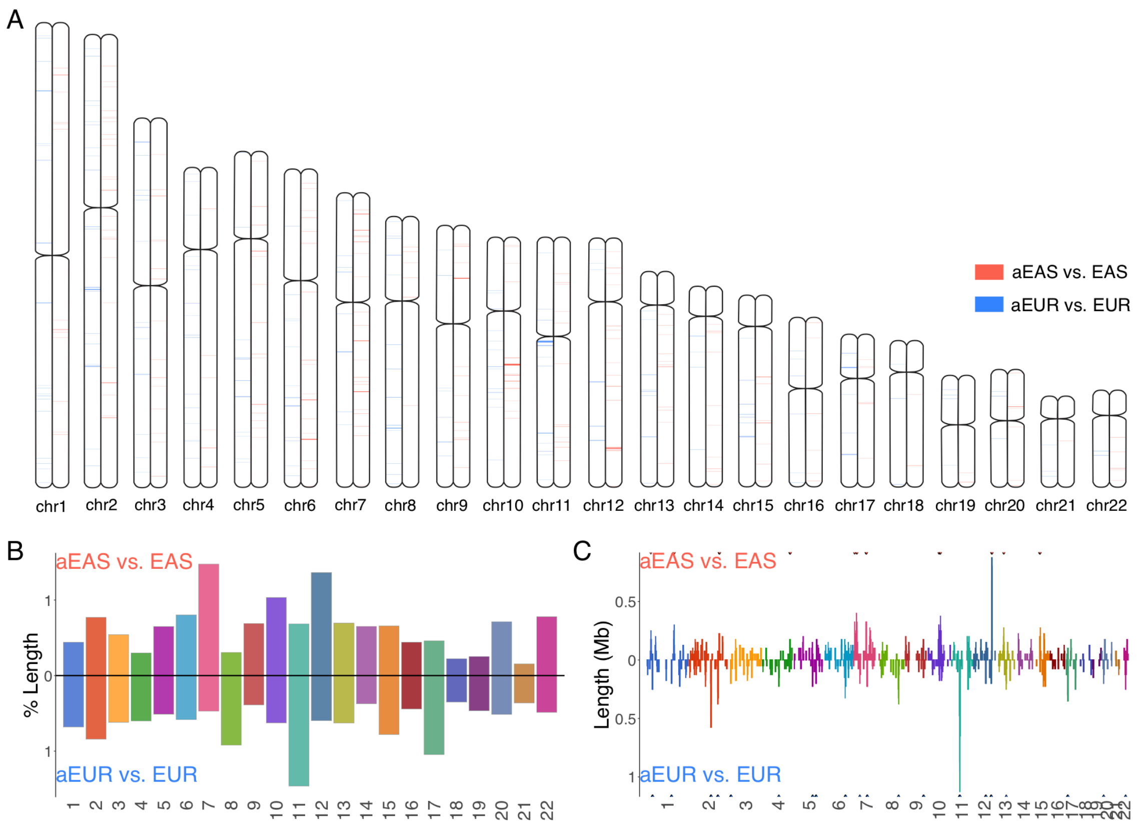

Figure S23.2 | Distribution of the AB_{aEAS} and AB_{aEUR} components.

A. Chromosome plot for AB_{aEAS} and AB_{aEUR} components identified in XJU; **B.** Proportions of the cumulative length of AB_{aEAS} and AB_{aEUR} components across chromosomes; **C.** Cumulative lengths of AB_{aEAS} and AB_{aEUR} components within sliding windows of 5 Mb in length shift by 1 Mb across the genome. Windows with *P*-values <0.001 (Poisson test on each chromosome, BH-corrected) were indicated by triangles.

Text S24. Ancestry-biased genetic components

There are some local components in XJU highly deviated away from the state of expected, due to post-admixture genetic drift, local adaption, and so on. We further investigated the

ancestry-biased (AB) local components by introducing the estimator AB index. The AB index was calculated for each local segment as the geometric mean of quantiles of both AFd_e and local ancestry deviation, which would be more robust than the product of empirical *P*-values. Totally 248 segments ranking the top 0.5% (AB index >0.95) were remained as the candidates of AB components, while 197 (79.4%) of them were in genic regions. And 119 out of the 248 segments (48.0%) had EAS-biased ancestry, while ancestry makeups of the other 129 (52.0%) were EUR-biased.

Analyses of the physical distributions of AB components revealed their over-/under-representation across different chromosomes. The proportion of cumulative length of AB component was 0.91% across the genome, and the highest proportions were on chromosome 6 (1.71%), 19 (1.56%), and 20 (1.98%), while the lowest were on chromosome 18 (0.16%), 21 (0.10%), and 22 (0%) (Fig. S24.1). Majority of the AB components identified on chromosome 5, 7, and 20 were EUR-biased, and most of those on chromosome 11, 13, and 15 were EAS-biased, while no EUR-biased component was found on chromosome 15.

We then investigated the regional enrichment of the AB components. The whole genome was partitioned into sliding windows of 5 Mb in length shift by 1 Mb. We counted the number of AB components within each sliding window, which was further fitted to the Poisson distribution for each chromosome. Regional enrichment of AB component was observed across the genome (Fig. S24.1), including the signature on chromosome 2 encompassing *LCT* (2q21.3), which was biased toward the EAS ancestry. There were 91 sliding windows with significant signals of enrichment for EAS-biased components (BH-corrected P <0.001). The most significant signals were found on chromosome 6, 13, and 15 (BH-corrected P =0). The EAS-biased components on chromosome 6 encompassed the MHC region, while those on chromosome 13 and 15 encompassed *LINC00381* (13q22.1) and *CCDC33* (15q24.1). For EUR-biased components, we identified 133 sliding windows with significant signals (BH-corrected P <0.001), and the most significant ones were found on chromosome 5, 6, 7, and 20 (BH-corrected P =0). The signal on chromosome 5 encompassed genes including *CTNNA1–SIL1* (5q31.2), while *SIL1* functions for protein translocation and folding in the endoplasmic reticulum and *CTNNA1* is related to actin filament binding. Genes including *GPRC6A* (6q22.1) and *NUS1* (6q22.1) contributed to

the signature on chromosome 6, which were respectively a member of the G protein-coupled receptor superfamily and a subunit of cis-prenyltransferase. For signatures on chromosome 7, there identified genes including *CALD1* (7q33), and *CALD1* plays an essential role in the regulation of muscle. The signal on chromosome 20 encompassed genes including *UQCC1* (20q11.22), which is involved in cytochrome b translation and stability.

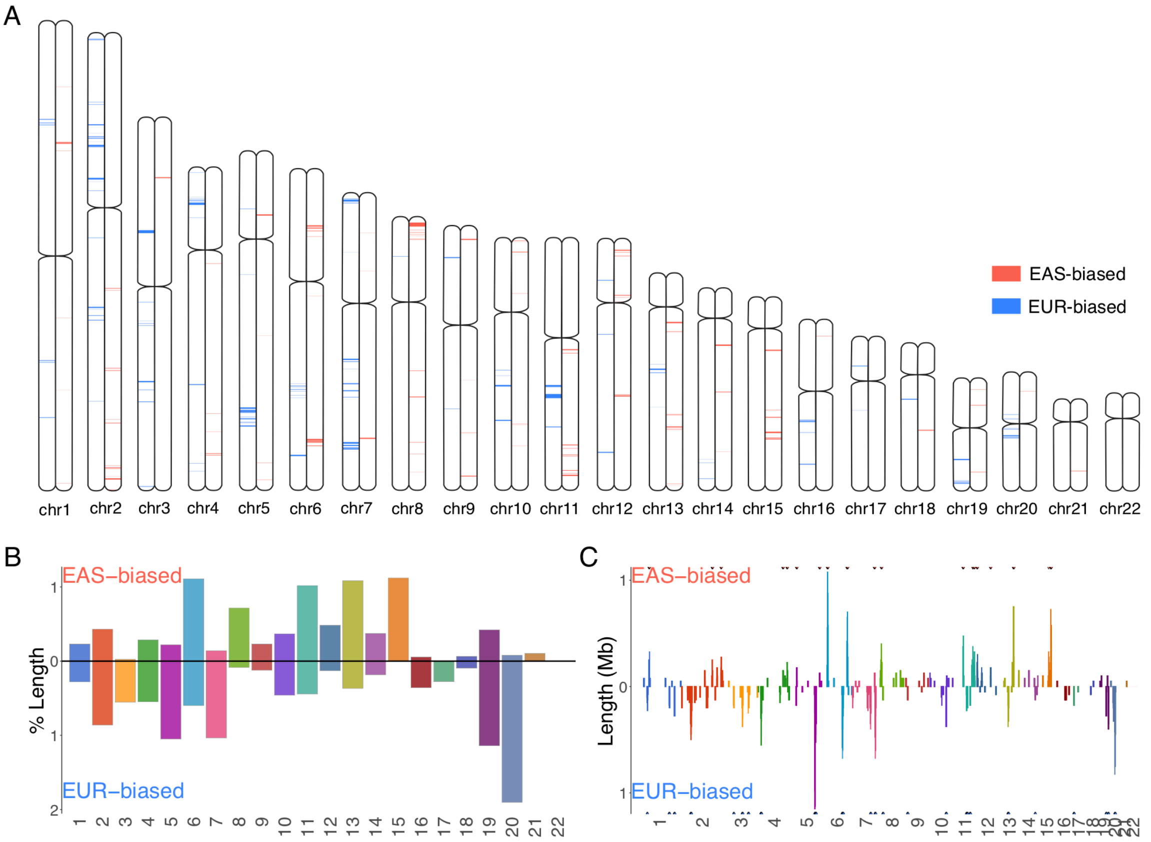

Figure S24.1 | Distribution of the AB components.
A. Chromosome plot for AB components identified in XJU; **B.** Proportions of the cumulative length of AB components across chromosomes; **C.** Cumulative lengths of AB components within sliding windows of 5 Mb in length shift by 1 Mb across the genome. Windows with *P*-values <0.001 (Poisson test on each chromosome, BH-corrected) were indicated by triangles.

LCT (2q21.3) is one of the genes ranking the top with high AFd_e (top 0.2%) and local ancestry deviation (~59% EAS ancestry). At the key variant rs4988235 (*LCT*-13910C>T) on *MCM6*, XJU had DAF equaling 0.05, and it was 0.0 in EAS, and 0.74 in EUR. *LCT* is well-known for its association with the lactose tolerance in European (80, 81). Significant

selection signals identified in EUR confirmed the divergent sweep after the emergence of agricultural civilization, while no signal of selection on *LCT* locus was identified in XJU using either the within- or between-population methods. The biased local ancestry and frequency profile of *LCT* could be resulted from the initial low frequency in the ancestral population (30). DAF of rs4988235 was <0.25 in the ancient EUR populations ~3,000 years ago (23) (Fig. S17). Network constructed using both modern and ancient human haplotypes revealed the high genetic diversity and low DAF of *LCT* in ancient EUR populations, with limited haplotypes remained in modern EUR populations due to selection (Fig. S21). Selection relaxation might be another factor influencing the genetic makeup of *LCT* in XJU. The demand for energy from milk may drive the evolution of EUR populations, while development of the agriculture civilization in East and Northeast Asia provided enough supply of lactose-free food for EAS and XJU in their daily life. *TMEM163* (2q21.3), locating ~1 Mb downstream to *LCT*, was reported to be associated with hair color (82) in EUR. Highly EAS-biased ancestry makeups were also identified on *TMEM163* (~58% EAS ancestry), consistent with its distribution of frequency profile. DAF of the key variant rs6739706 is 0.766 in XJU, 0.825 in EAS, and 0.364 in EUR.

Both Gene Ontology (GO) (83) and KEGG enrichment analyses were conducted for the AB components. The results revealed that EAS-biased components were enriched for genes related to immunity and sensory perception of smell, and the EUR-biased components were associated with the pathways related to cytotoxicity and antigen processing (BH-corrected P <0.05, Fisher-exact test) (Table S10). It suggests the admixture-gain of the functional components in XJU from ancestral populations, especially those related to stimuli perception. Both OR genes and immune-related genes have the highest genetic variability across the genome (84), which corresponds to their ability to perceive and process huge amounts of environmental stimuli, including the chemical compounds, bacteria, and virus in the environment. The sensory and immune systems have to evolve rapidly for their adaptation to the local environments. Though the high genetic variability for both sensory and immunity systems may influence the local ancestry inference, similar results were also achieved by the inferred local ancestry based on 4 references. For the 4-reference-based local ancestry inference, we grouped together the haplotypes of EAS and SIB ancestries, as well those of EUR and SAS ancestries. Sliding

windows were ranked based on either the EAS/SIB or EUR/SAS ancestry proportions, and those with more extreme ancestry proportions had higher ranks. The empirical *P*-value for each window was then calculated as the percentage of windows with higher ranks. AB index were re-estimated for each segment as the geometric mean of quantiles of AFd_e and local ancestry deviation from 4-reference analyses. Totally 245 segments ranking the top 0.5% were remained, with 138 (56.3%) having EAS-biased ancestry and the other 107 (43.7%) having EUR-biased ancestry. There also identified enrichment of EAS/SIB-biased components for pathway genes related to "Olfactory transduction" (hsa04740). And the EUR/SAS-biased components were enriched for genes related to immune-related pathways, including antigen processing (e.g., GO:0002476 and GO:0002428) (Table S10).

Text S25. Genetic components of XJU with Archaic ancestry

Archaic segments are one kind of the genetic markers along the genomes of XJU. Since the contacts between archaic and modern human were much earlier than the admixture between West- and East-Eurasian populations across the Eurasian continent, archaic segments in XJU were expected to be inherited from the ancestral source populations. We applied ArchaicSeeker 2.0 (5) to screen segments of archaic ancestry with all parameters set to default, while the analyzed dataset was merged from the whole-genome sequencing data of XJU, EAS, EUR, SAS, and SIB populations. The individual mean proportions of Denisovan ancestry were estimated as 0.089%, 0.114%, 0.051%, 0.101%, and 0.115% for XJU, EAS, EUR, SAS, and SIB, respectively (Fig. S25.1A). And the mean proportions of Neanderthal ancestry were 1.252%, 1.433%, 1.113%, 1.183%, and 1.407% for XJU, EAS, EUR, SAS, and SIB individuals, respectively (Fig. S25.1B). The deviation was ~0.06% between the observed Denisovan ancestry proportion and that of expected for XJU individuals, while the it was ~0.02% for the Neanderthal ancestry.

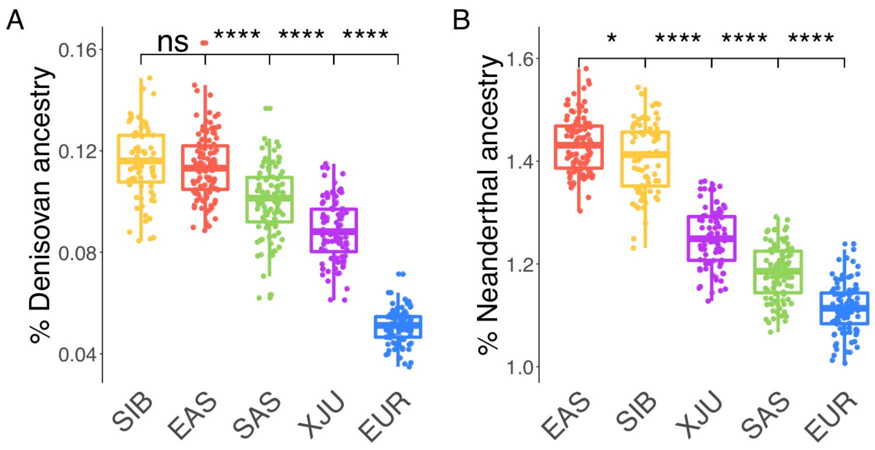

Figure S25.1 | Distributions of individual archaic ancestry proportions among XJU, EAS, EUR, SAS, and SIB.

A. Distribution of genome-wide Denisovan ancestry proportions among XJU, EAS, EUR, SAS, and SIB individuals; **B.** Distribution of genome-wide Neanderthal ancestry proportions among XJU, EAS, EUR, SAS, and SIB individuals.

Further, we calculated the frequencies of local archaic haplotypes, while haplotypes of <15Kb in length were dropped to minimize the potential uncertainty in archaic segment detection. There identified one region with Denisovan ancestry on chromosome 6 (chr6:81267698-81287854) with frequencies of 21.74%, 10.68%, 16.67%, 8.74%, and 4.79% in XJU, EAS, EUR, SAS, and SIB, respectively. XJU carries >5% more Denisovan ancestry haplotypes than all of the other reference populations. There harbored 2 variants totally linked on this region with significant AFd_e, rs11752731 and rs4639292, whose alternative alleles were homozygous in Denisova but not presented in YRI. The alternative allele frequencies were 21.74%, 11.17%, 16.67%, 9.71%, and 6.16% in XJU, EAS, EUR, SAS, and SIB, respectively, consistent with the frequencies of archaic haplotypes. A pseudogene (*RP11-486E2.1*) was found ~800bp downstream to the region.

For the Neanderthal ancestry components, we found 2 regions with archaic frequency significantly higher than that of expected in XJU. The first one was ~15Kb in length, located on chromosome 6 (chr6:74764733-74780250), with archaic frequency equaling 17.93%, 9.22%, 12.63%, 10.19%, and 4.11% in XJU, EAS, EUR, SAS, and SIB, respectively. Totally 19 Neanderthal-specific variants were identified, which were homozygous in Neanderthal but absent in YRI. This region was linked to *RP11-554D15.1*, which is a lincRNA. The second region was identified on chromosome 12

(chr12:40187069-40208037) with ~20Kb in length, harboring 2 Neanderthal-specific variants (rs7975209 and rs7969296). The archaic frequency was 8.15%, 2.43%, 2.53%, 1.46%, and 2.05% in XJU, EAS, EUR, SAS, and SIB, respectively. The region was related to *C12orf40* and *SLC2A13*, while both of them were protein-coding genes. Gene *SLC2A13* (Solute Carrier Family 2 Member 13) is associated with "myo-inositol:proton symporter activity" (GO:0005366) and "transmembrane transporter activity" (GO:0022857) (83), as well the inflammatory bowel disease and Crohn's disease (85). *C12orf40* was related to "protein binding" (GO:0005515) (83).

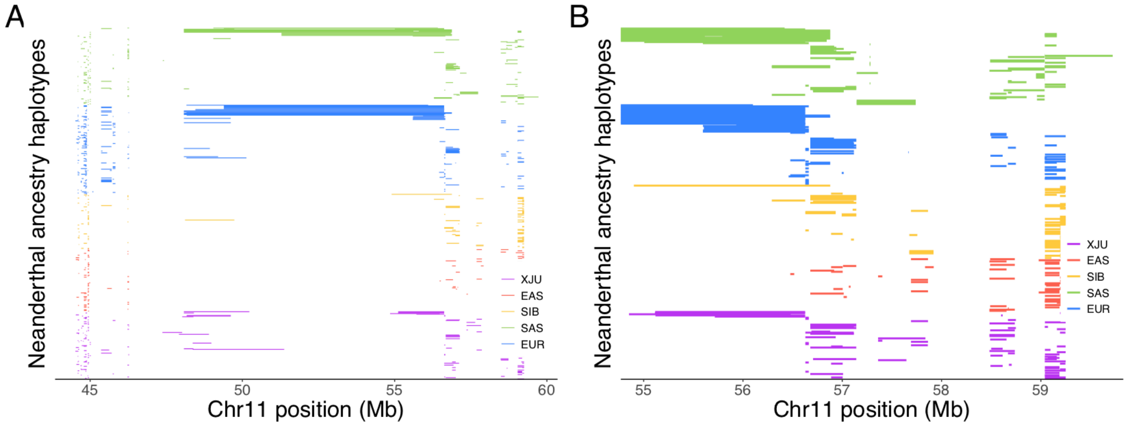

Figure S25.2 | Neanderthal ancestry haplotypes in the centromeric region on chromosome 11 among XJU, EAS, EUR, SAS, and SIB.
A. Neanderthal haplotypes within the region chr11:44600000-59600000; **B.** Neanderthal haplotypes within the region chr11:55000000-59600000.

Moreover, we identified a large Neanderthal haplotype spanning across the centromeric region on chromosome 11 (44.6–59.6 Mb), about 15 Mb in length. The Neanderthal haplotype segregates in XJU, EUR, SAS, and SIB populations, and its frequency is ~2.99% in XJU, ~12.12% in EUR, 6.80% in SAS, and 0.07% in SIB (Fig. S25.2). Population substructure was also observed due to the Neanderthal ancestry components (Fig. S25.3), as the outliers in the PCA plots corresponds to the individuals carrying the Neanderthal haplotype. Haplotype structures were revealed based on 3,565 Neanderthal segregating sites, which were defined as SNVs with large allele frequency differentiation between the outlier samples in the PCA plots and the others ($F_{\mathrm{ST[XJU.outlier\ vs.\ XJU.other]}} > 0.2$ and $F_{\mathrm{ST[EUR.outlier\ vs.\ EUR.other]}} > 0.2$) (Fig. S25.3G, H). More than half of the

Neanderthal segregating sites were identified with regulatory function according to GTEx (50), while some of them were LoF/missense variants (Fig. S25.4).

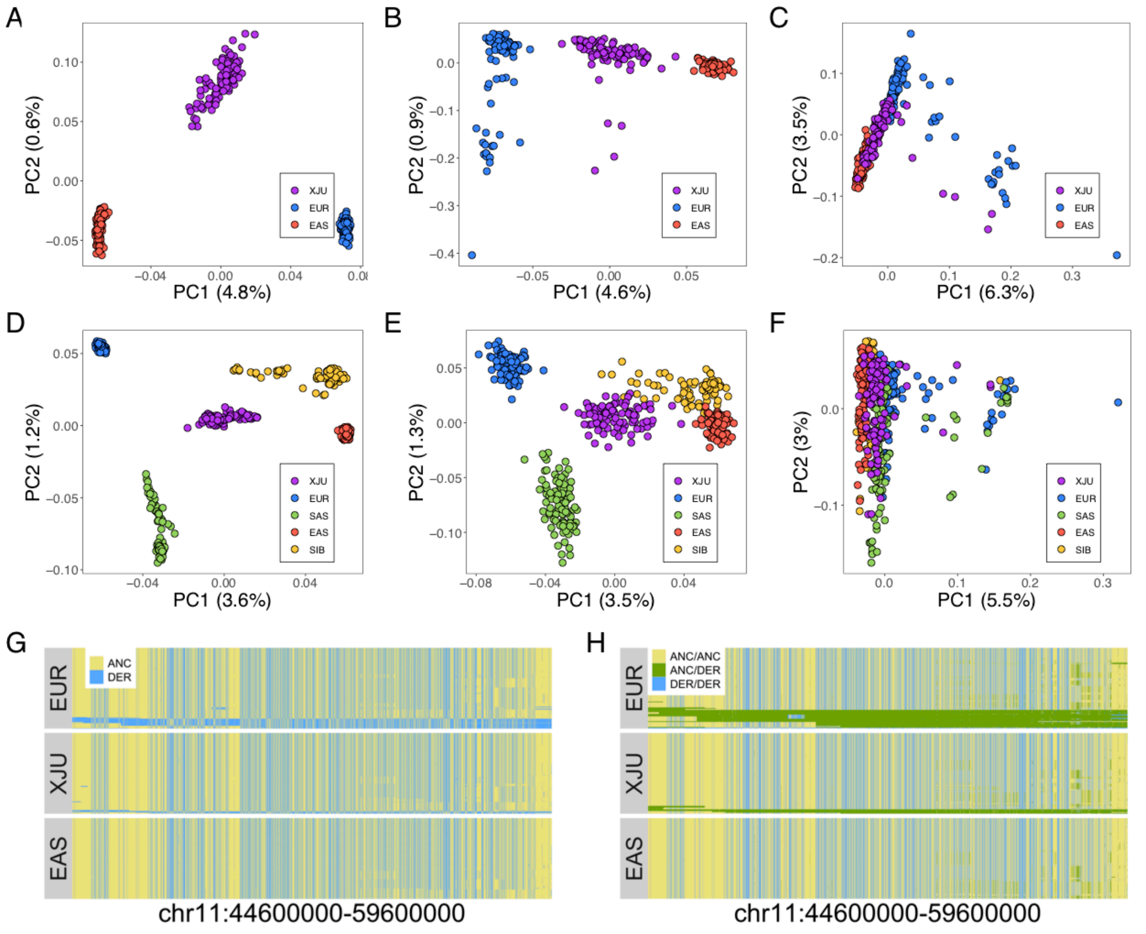

Figure S25.3 | Population substructures in XJU, EUR, SAS, and SIB due to the Neanderthal haplotypes.

A. PCA of XJU, EAS, and EUR using the genome-wide data; **B**. PCA of XJU, EAS, and EUR using only chromosome 11; **C.** PCA of XJU, EAS, and EUR based on the region chr11:44600000-59600000; **D.** PCA of XJU, EAS, EUR, SAS, and SIB using the genome-wide data; **E.** PCA of XJU, EAS, EUR, SAS, and SIB using only chromosome 11; **F.** PCA of XJU, EAS, EUR, SAS, and SIB based on the region chr11:44600000-59600000. We pruned the merged data set by excluding one variant from each pair closer than 5 Kb using PLINK1.9 (--bp-space 5000) to eliminate the influence of the linkage disequilibrium (LD); **G.** Haplotypes of XJU, EAS, and EUR samples. Each line indicates a haplotype and every column represents a variant; **H.** Genotypes of XJU, EUR, and EAS samples. Each line indicates an individual and every column represents a variant. Haplotypes were constructed using SNVs with $F_{ST[XJU.outlier\ vs.\ XJU.other]} > 0.2$ and $F_{ST[EUR.outlier\ vs.\ EUR.other]} > 0.2$, while XJU.outlier and EUR.outlier denotes the outliers in the PCA plots.

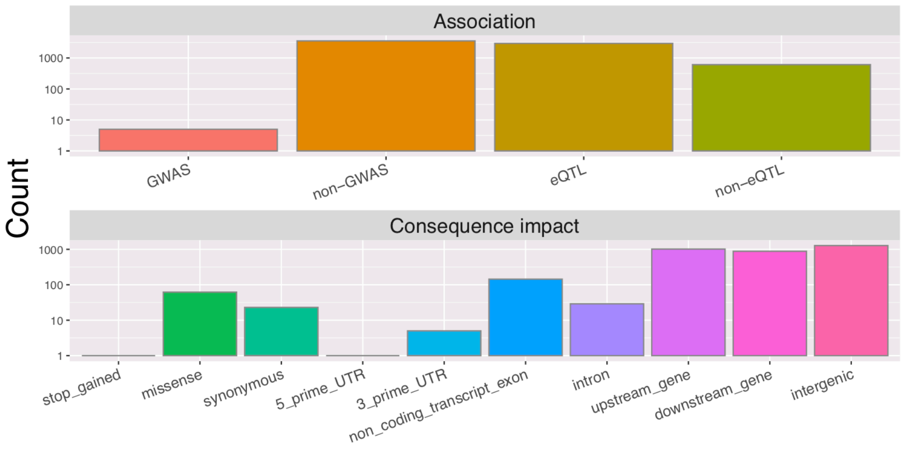

Figure S25.4 | Functional annotation of the Neanderthal segregating sites.

Notably, a large cluster of OR genes were also found within the region mentioned above and downstream to the centromere (chr11: 55350000–59600000), consistent with the findings in the previous studies (86, 87). This OR gene cluster encompassed genes including *OR5W2*, *OR4A16*, *OR5A2*, *OR4D6*, *OR4C15*, *OR10Q1*, and so on, while they have been reported to be associated with variation of human olfactory perception of particular volatile chemicals (88), such as Menthol. Signatures of EAS-biased ancestry were also observed among the OR genes, resulting in the enrichment of olfactory perception for the EAS-biased genetic components. No signal of natural selection was found in this region.

Supplementary Figures

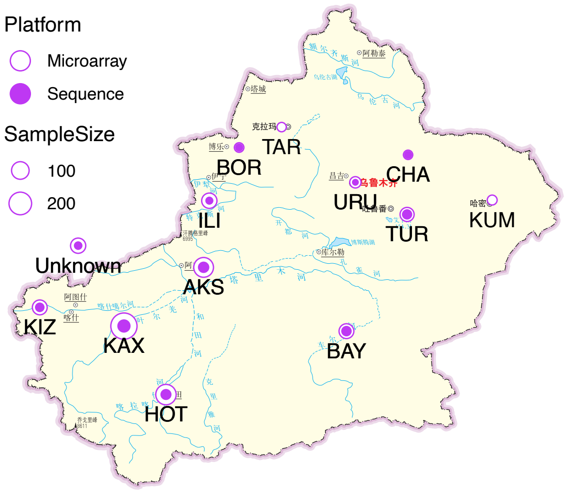

Figure S1 | Geographical distribution and sample size of XJU groups.

Rough locations of the XJU samples with sequenced genomes in this study are shown, as well as those with microarray data. The 92 XJU individuals with whole genome sequencing data were collected from 9 prefectures (Kaxgar, Hotan, Kizilsu, Aksu, Bayingolin, Turpan, Changji, Ili, and Bortala) and 1 prefecture-level city (Urumqi) in Xinjiang Uyghur Autonomous Region, China.

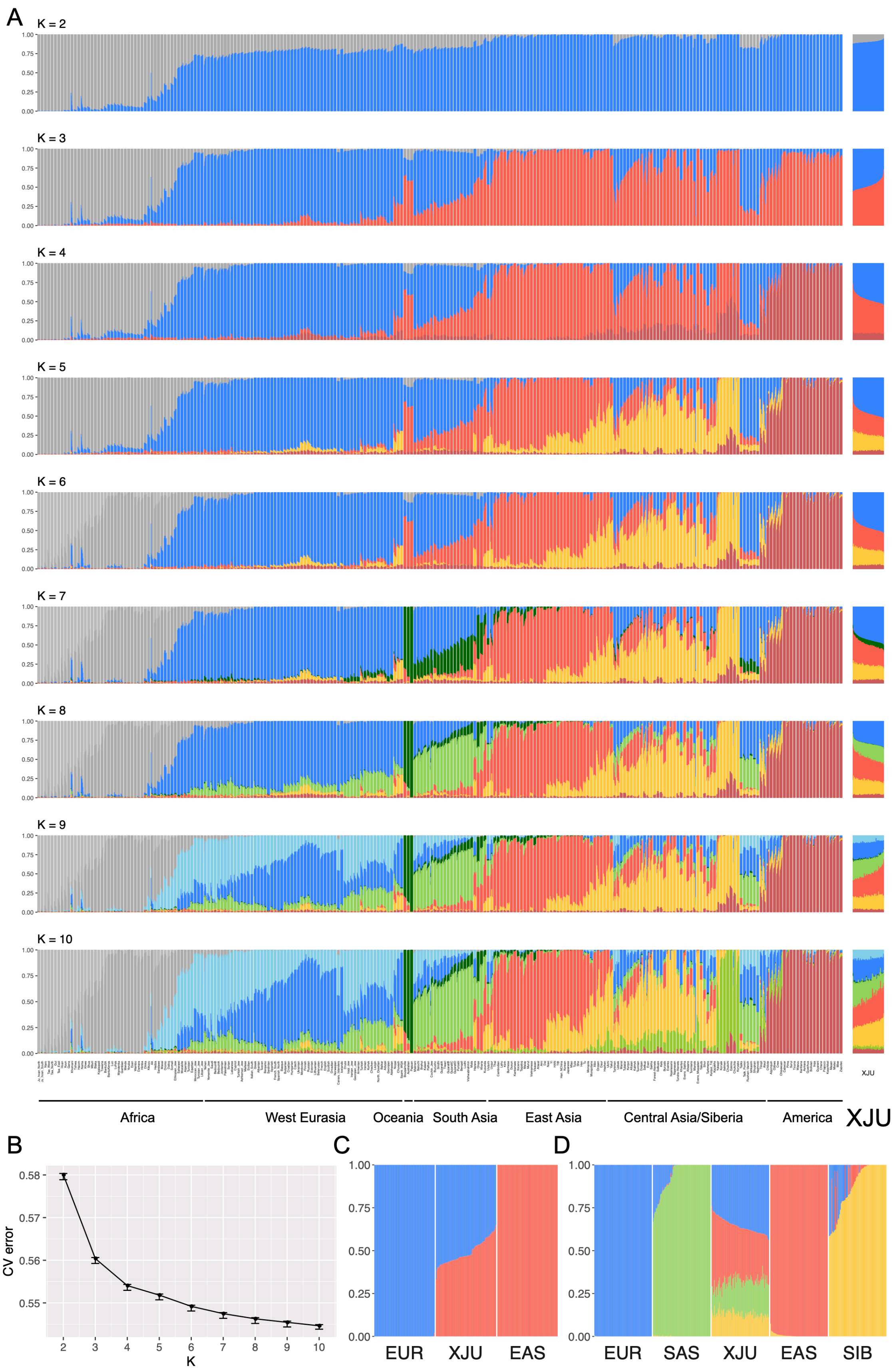

A
K = 2
K = 3
K = 4
K = 5
K = 6
K = 7
K = 8
K = 9
K = 10
1.00
0.75
0.50
0.25
0.00
XJU
Africa
West Eurasia
Oceania
South Asia
East Asia
Central Asia/Siberia
America
XJU
B
CV error
0.58
0.57
0.56
0.55
K
2
3
4
5
6
7
8
9
10
C
EUR
XJU
EAS
D
EUR
SAS
XJU
EAS
SIB

Figure S2 | ADMIXTURE analysis of XJU with the reference populations.

A. ADMIXTURE results of XJU with world-wide populations from K=2 to K=10; **B**. CV errors of ADMIXTURE analysis. Error bars indicate the range of CV errors across 10 replicates; **C**. ADMIXTURE results of XJU with EUR and EAS populations at K=2; **D**. ADMIXTURE reaults of XJU with EUR, SAS, EAS, and SIB populations at K=4. Admixture proportions of each individual were averaged across 10 replicates. Each individual was represented by a vertical line, which was partitioned into segments corresponding to the ancestral clusters indicated by color.

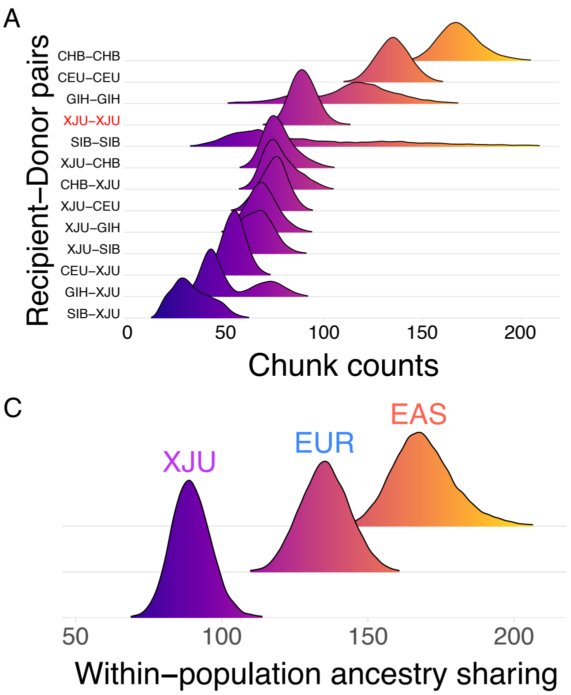

Figure S3 | Ancestry sharing among XJU and reference populations.

Cross-population ancestry sharing among XJU and the reference populations indicated by sharing **A.** chunk counts and **B.** chunk lengths, which were estimated by CHROMOPAINTER; **C**. Within-population ancestry sharing for XJU, EAS, and EUR individuals estimated from the co-ancestry matrix using Chromopainter; **D**. Within-population identical-by-state (IBS) for XJU, CHB, and CEU individuals.

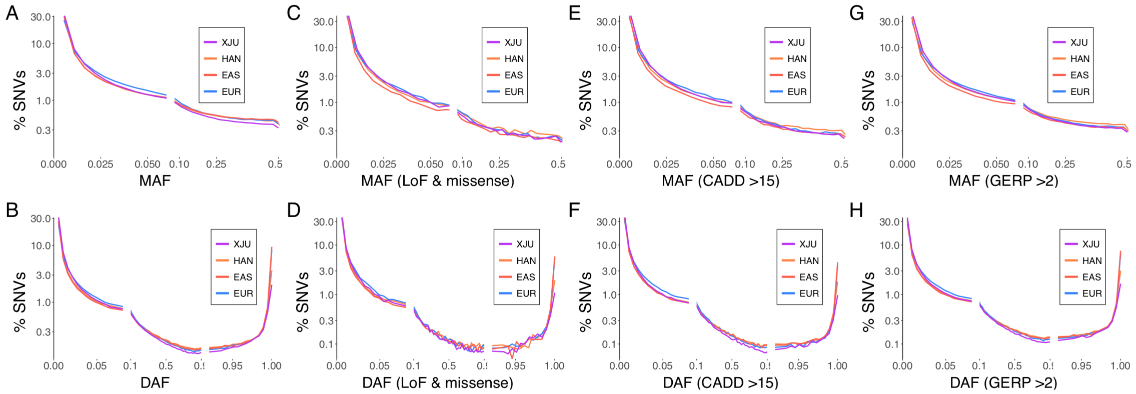

Figure S4 | Site frequency spectrum of XJU, HAN, EAS, and EUR.

Minor allele frequency (MAF) spectrum for **A.** genome-wide SNVs, **C.** SNVs with high and moderate consequence impact, **E.** SNVs with CADD score >15, and **G.** SNVs with GERP score >2. Proportions were estimated within MAF bins of size 0.01; Derived allele frequency (DAF) spectrum for **B.** genome-wide SNVs, **D.** SNVs with high and moderate consequence impact, **F.** SNVs with CADD score >15, and **H.** SNVs with GERP score >2. Proportions were estimated within DAF bins of size 0.01.

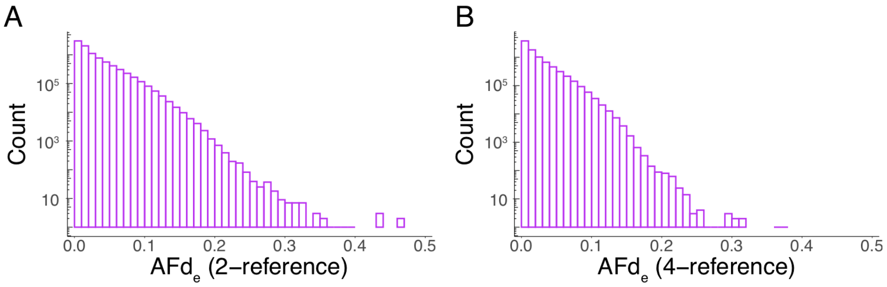

Figure S5 | Distribution of frequency deviation between observed allele frequencies and those of expected.

Distribution of AFd$_e$ estimated between observed allele frequency of XJU and that of expected. The expected allele frequency of XJU were estimated using **A.** 2 reference populations and **B.** 4 reference populations.

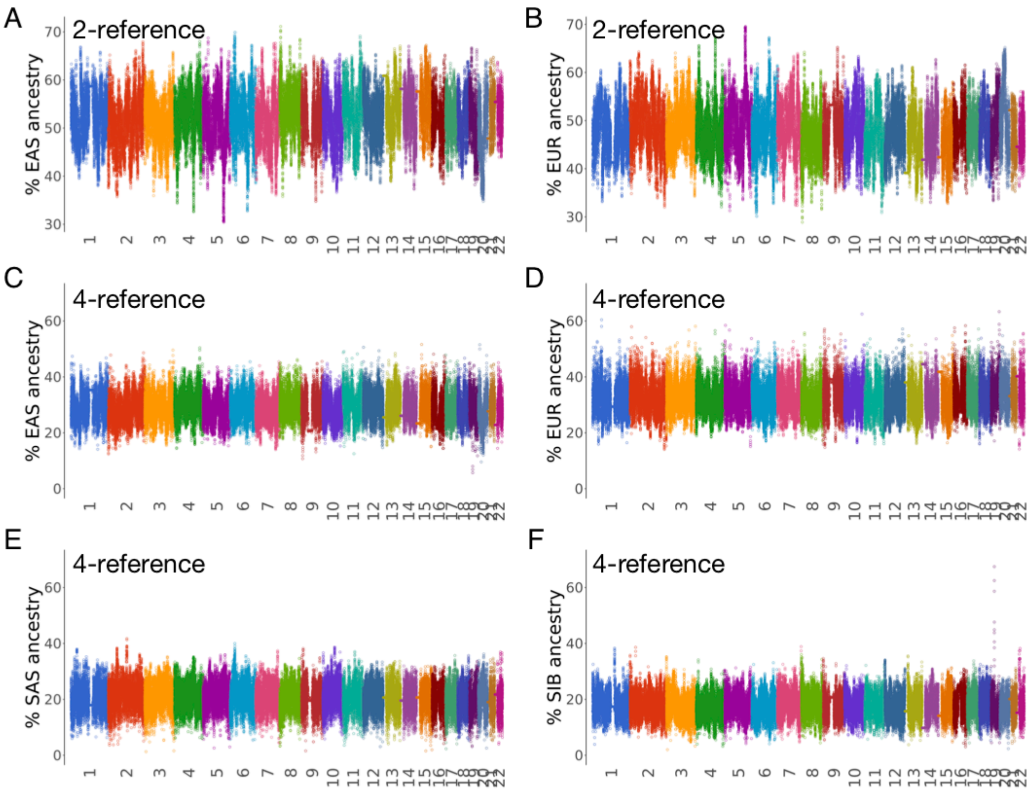

Figure S6 | Local ancestry of XJU.

Local ancestry of XJU using **A–B.** 2 reference populations and **C–F.** 4 reference populations. Local ancestry proportions were re-estimated as the average within sliding windows of 50 Kb in length shift by 25 Kb across the genome.

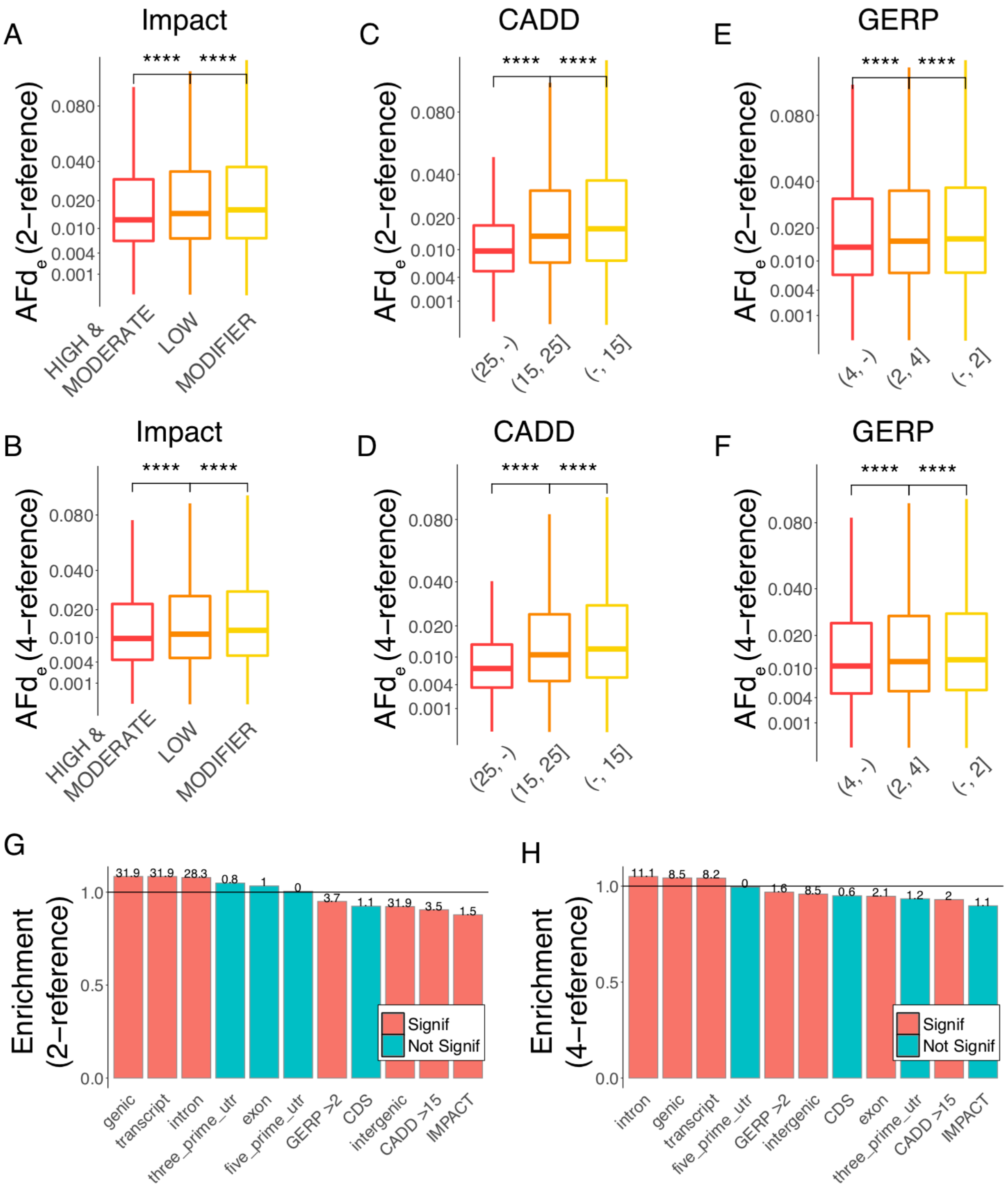

Figure S7 | Variant conservation and frequency deviation.

We estimated AFd_e using 2 reference populations, and comparisons of AFd_e were conducted among variants of different **A.** consequence impact, **B.** CADD score, and **C.** GERP score. Comparisons of 4-referene-based AFd_e were also conducted for variants of different **D.** consequence impact, **E.** CADD score, and **F.** GERP score. **G–H.** We analyzed the enrichment of SNVs with large AFd_e (empirical P <0.01) in regions of different biotypes. The genic, intergenic, transcript, exon, intron, 5'-UTR, and 3'-UTR components were annotated based on the GTF annotation from Ensembl (release 90). "IMPACT" denotes the SNVs of high and moderate consequence impacts. The y-axis indicates the odds ratio, and the numbers labeled on the bars indicate the $-\log_{10}$(BH-corrected P). Enrichment analyses passed the threshold (Fisher-exact test, BH-corrected P <0.05) were colored in red.

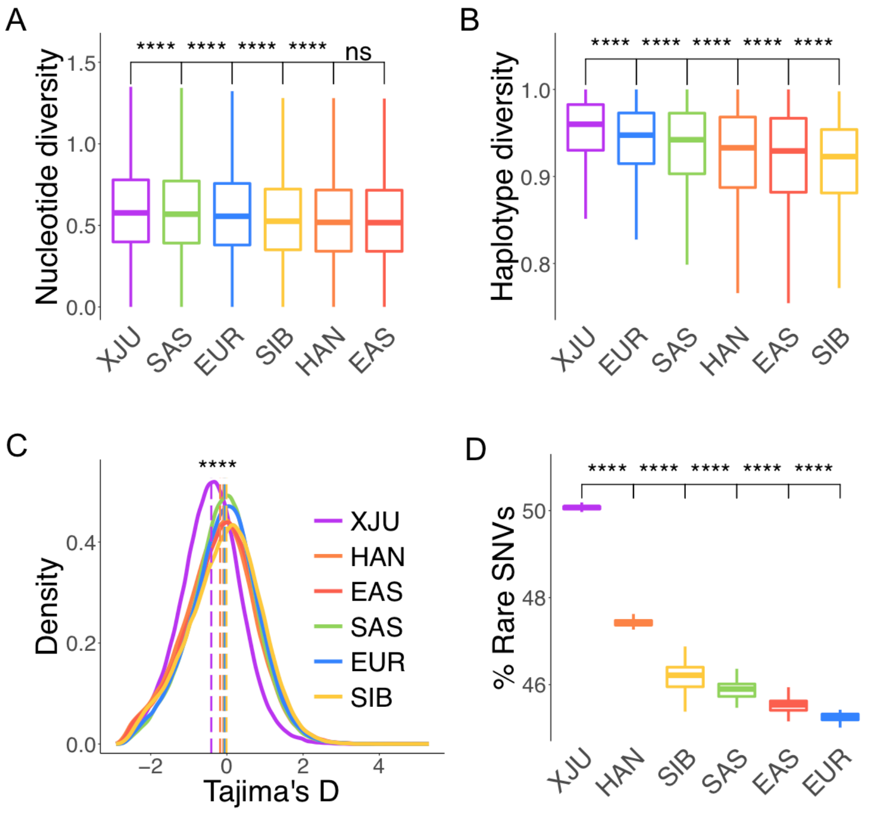

Figure S8 | Genetic diversity of XJU and the reference populations.

A. Nucleotide diversity (/Kb) of XJU and the reference populations; **B.** Haplotype diversity; **C.** Distribution of Tajima's D statistics. All of nucleotide diversity, haplotype diversity, and Tajima's D were estimated using 50 random sampled individuals within sliding windows of 50 Kb across the genome; **D.** Rare SNV proportion in XJU and the reference populations. It was calculated as the proportion of rare SNVs (AF <0.05) among 50 random sampled individuals. Totally 100 replicates were conducted.

Outliers were not show on figures. Statistical significance of Wilcoxon rank sum test: ns: p > 0.05; *: p <= 0.05; **: p <= 0.01; ***: p <= 0.001; ****: p <= 0.0001

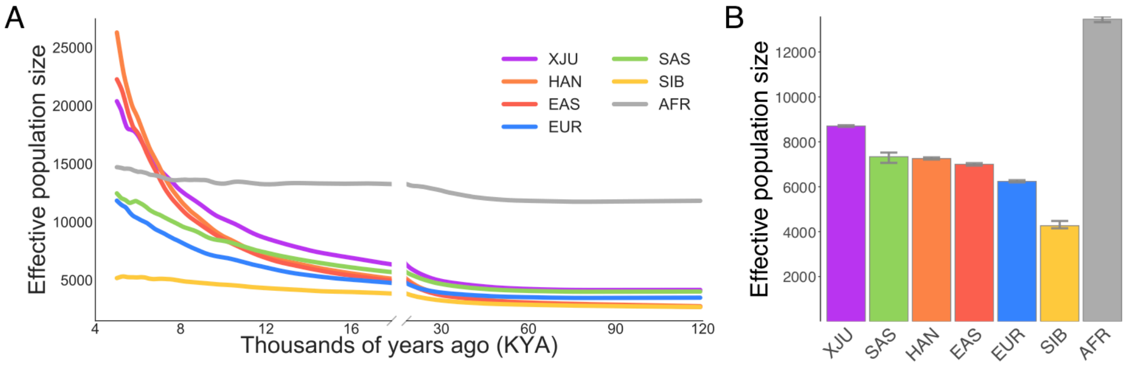

Figure S9 | Effective population size of XJU and the reference populations.

A. Effective population sizes (N_e) of XJU and the reference populations along the history; **B.** Long-term N_e of XJU and the reference populations. Totally 10 replicates were conducted, with 50 samples sampled at random without replacement for each. Median values were used for effective population sizes along the history in the plot. The error bar for each population indicated the range of maximum and minimum effective population sizes estimated among the 10 replicates.

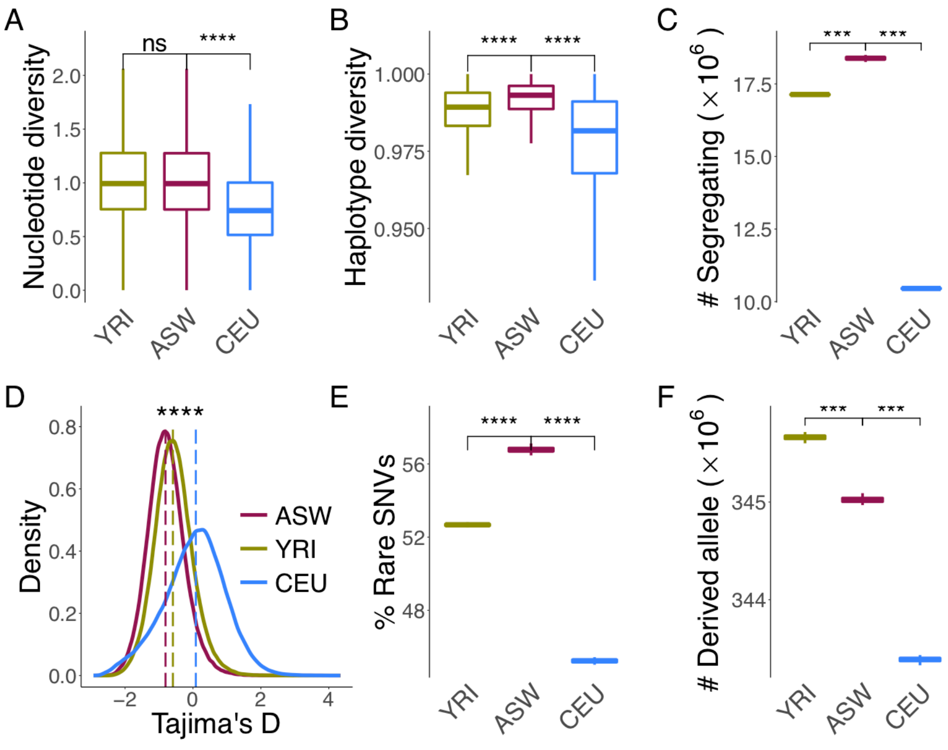

Figure S10 | Genetic diversity of ASW and the reference populations.

A. Nucleotide diversity (/Kb) of ASW and the reference populations; **B.** Haplotype diversity. Both of nucleotide diversity and haplotype diversity were estimated using 50 random sampled individuals within sliding windows of 50 Kb across the genome; **C.** Number of segregating sites (million) across the genomes. There conducted 100 replicates, while 50 individuals were random sampled for each; **D.** Distribution of Tajima's D statistics; **E.** Rare SNV proportion. It was calculated as the proportion of rare SNVs (AF <0.05) among 50 random sampled individuals, and 100 replicates were conducted; **F.** Numbers of derived alleles (million). Totally 100 replicates were conducted, with 50 individuals random sampled for each replicate.

Outliers were not show on figures. Statistical significance of Wilcoxon rank sum test: ns: p > 0.05; *: p <= 0.05; **: p <= 0.01; ***: p <= 0.001; ****: p <= 0.0001

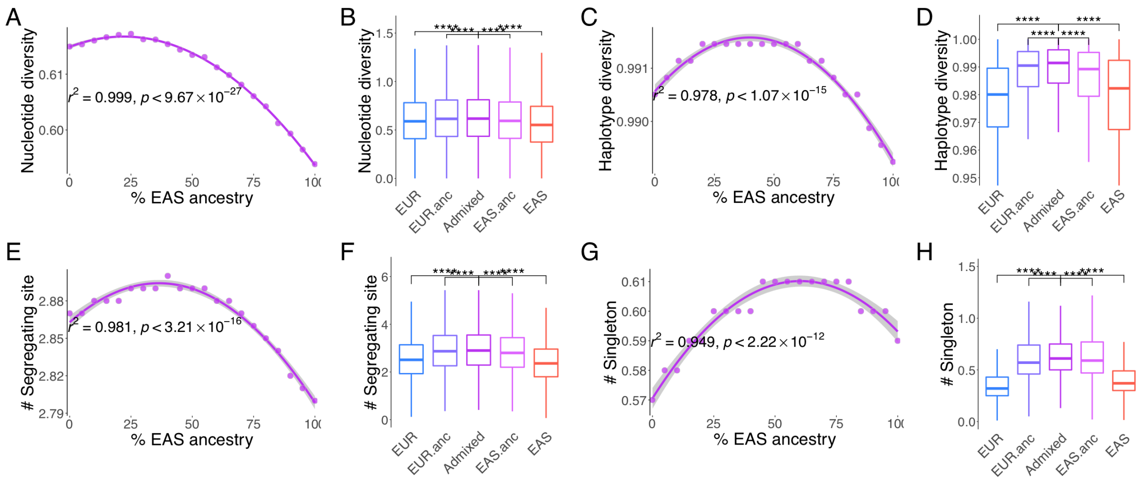

Figure S11 | Association between local ancestry and genetic diversity.

Association between EAS ancestry and **A.** nucleotide diversity, **C.** haplotype diversity, **E.** number of segregating sites, and **G.** number of singletons, which were estimated within sliding windows of 50 Kb using 80 haplotypes. Median values of genetic diversity were employed under the given ancestry proportions. The curves were fitted using the function "lm" in R; Genetic diversity estimated as **B.** nucleotide diversity, **D.** haplotype diversity, **F.** number of segregating site, and **H.** number of singletons for EUR, EAS, EAS-specific haplotypes in XJU, and EUR-specific haplotypes in XJU, as well as the ancestry-mixed haplotypes in XJU with the highest genetic diversity. The genetic diversity was also estimated within sliding windows of 50 Kb using 80 random sampled haplotypes.

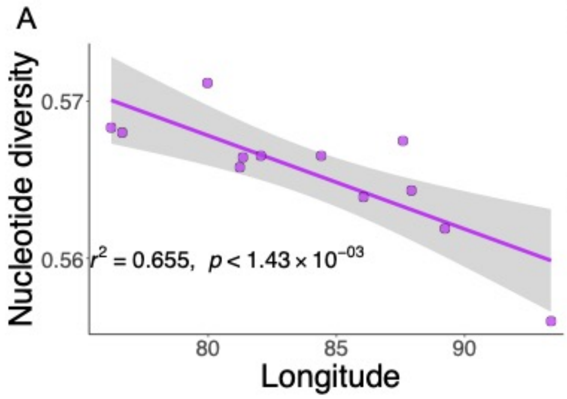

Figure S12 | Association between geographic coordinates and genetic diversity as well effective population size for regional XJU groups.

A. Association between geographic coordinates and genetic diversity for regional XJU groups. Genetic diversity was estimated as the nucleotide diversity; **B.** Association between geographic coordinates and effective population size for regional XJU groups. We estimated both genetic diversity and effective population size using the microarray data. And 10 individuals were random sampled from each group to balance the sample size.

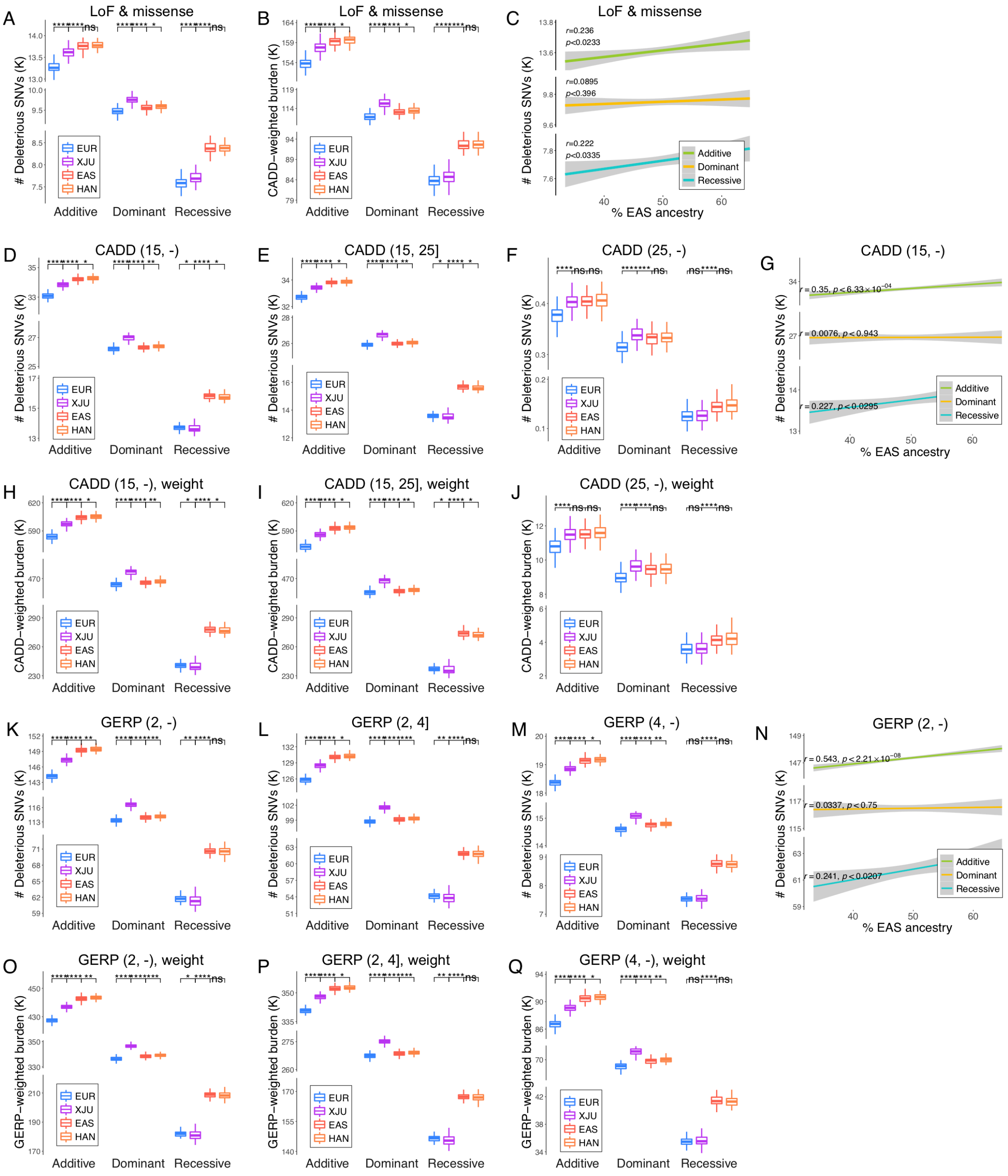

Figure S13 | Individual genetic burden of XJU and the reference populations.

A. Individual genetic burden estimated as the counts of LoF and missense variants for XJU, HAN, EAS, and EUR under the additive, dominant, and recessive models; **B.** Individual genetic burden estimated as the CADD-score-weighting counts of LoF and missense variants; **C.** Association between genetic burden and global ancestry proportion for XJU individuals. Individual genetic burden was estimated as the counts of LoF and missense variants; Individual genetic burden estimated as the counts of variants with CADD score **D.** >15, **E.** ranging (15, 25], and **F.** >25; **G.** Association between genetic burden and global ancestry proportion for XJU individuals. Individual genetic burden was estimated as the counts of variants with CADD score >15; Individual genetic burden estimated as the CADD-score-weighting counts of variants with CADD score **H.** >15, **I.** ranging (15, 25], and **J.** >25; Individual genetic burden estimated as the counts of variants with GEPR score **K.** >2, **L.** ranging (2, 4], and **M.** >4; **N.** Association

between genetic burden and global ancestry proportion for XJU individuals. Individual genetic burden was estimated as the counts of variants with GERP score >2. Individual genetic burden estimated as the GERP-score-weighting counts of variants with GERP score **O.** >2, **P.** ranging (2, 4], and **Q.** >4. Statistical significance of Wilcoxon rank sum test: ns: p > 0.05; *: p <= 0.05; **: p <= 0.01; ***: p <= 0.001; ****: p <= 0.0001

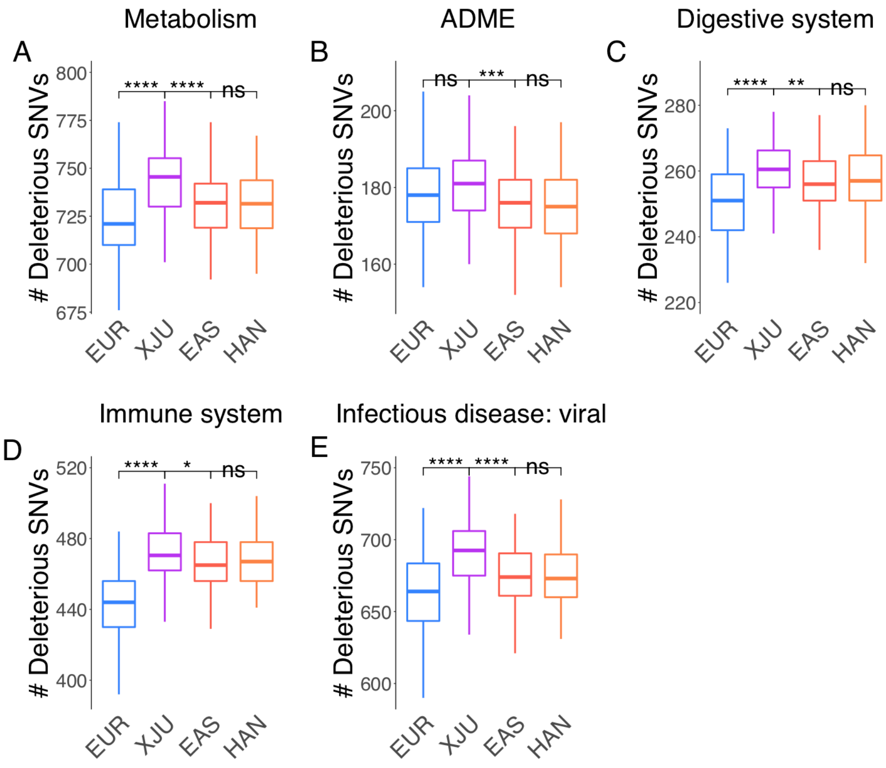

Figure S14 | Genetic burden of pathways.

Genetic burden was estimated as the counts of LoF and missense variants within the gene regions of the given pathway for each individual under the dominant model.

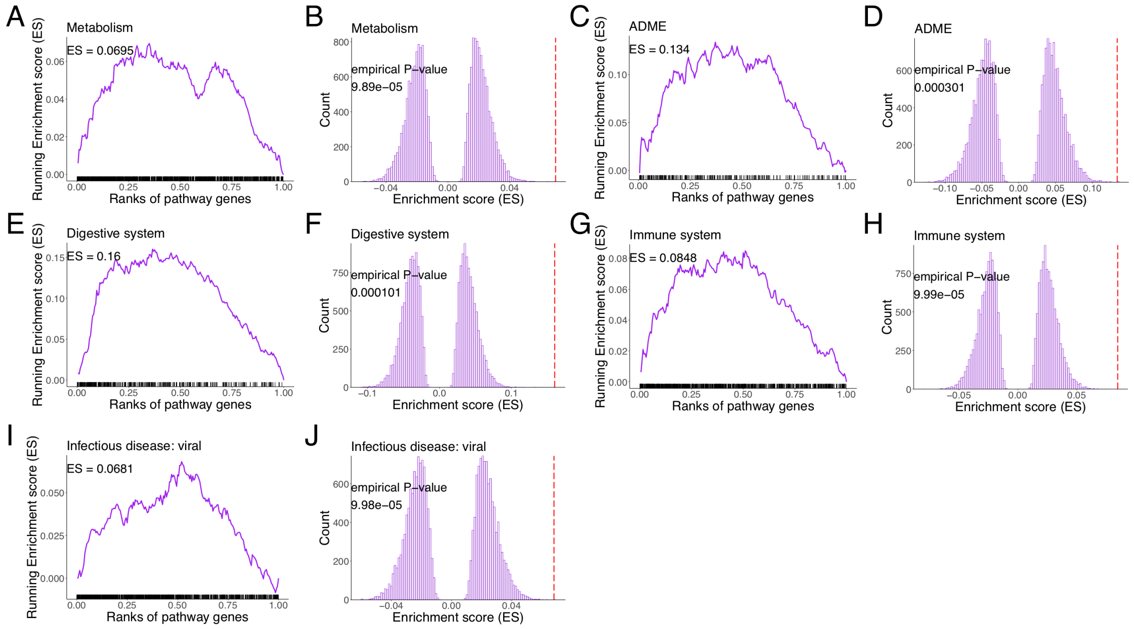

Figure S15 | Analysis of mGSEA for pathway genes.

Running enrichment score (ES) and positions of the pre-ranked gene list for pathways related to **A.** Metabolism, **C.** ADME, **E.** Digestive, **G.** Immune, and **I.** Infectious disease; Distributions of ES from permutation analyses for pathways related to **B.** Metabolism, **D.** ADME, **F.** Digestive, **H.** Immune, and **J.** Infectious disease. The vertical lines indicated the ES of real data.

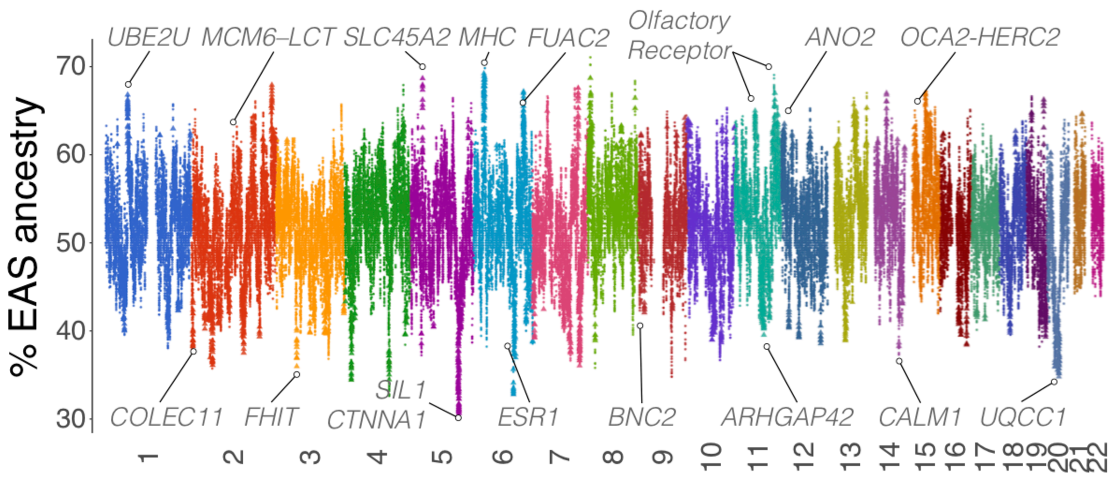

Figure S16 | Ancestry-biased (AB) genetic components.

Signatures of AB components were indicated by the AB index, which combined both allele frequency and local ancestry deviation of XJU's genome from that of expected. The y-axis indicated the local ancestry of XJU. Outliers identified by AB index were in triangle shape.

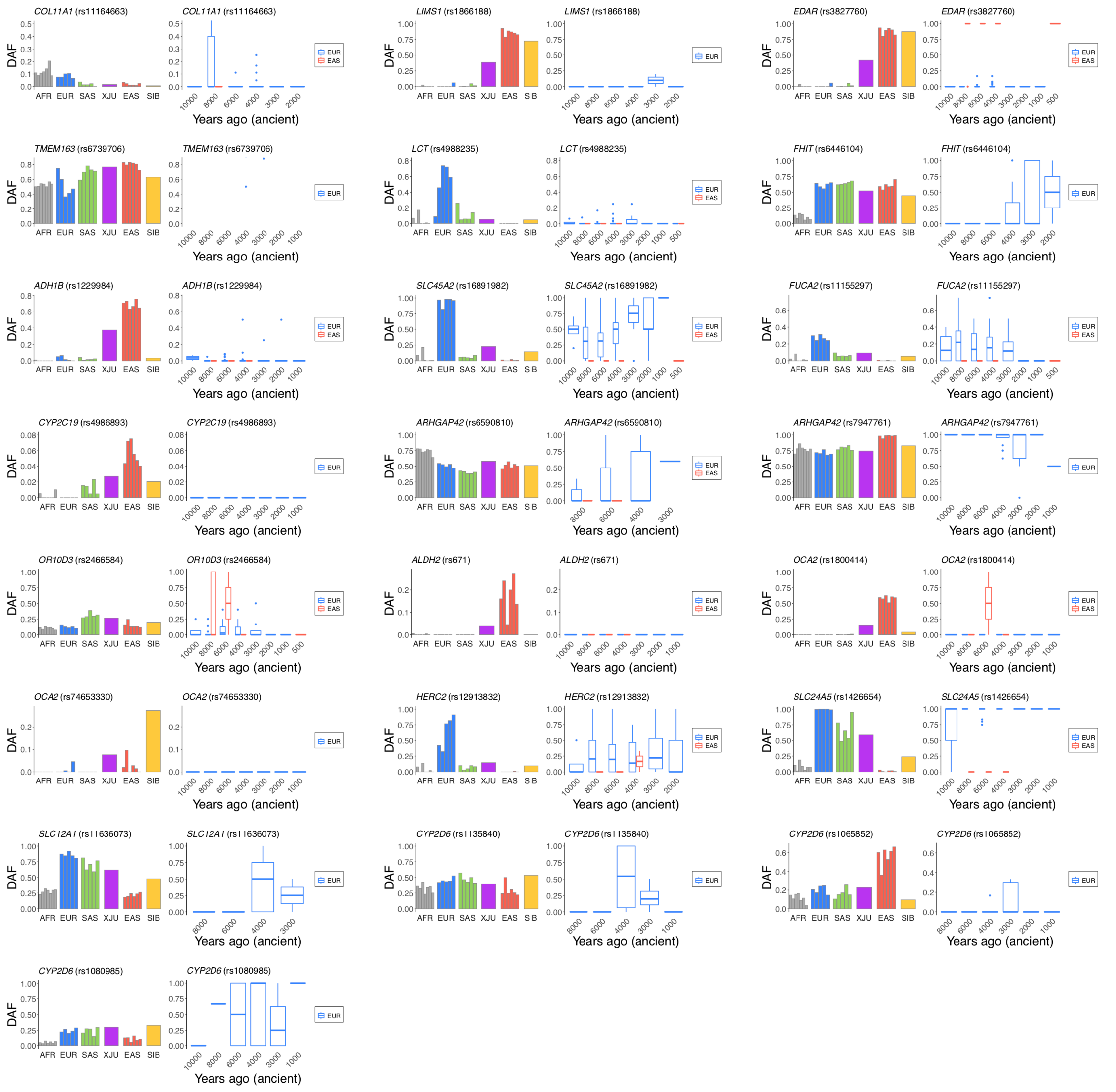

Figure S17 | DAF of some key variants among world-wide modern human populations and ancient human populations.

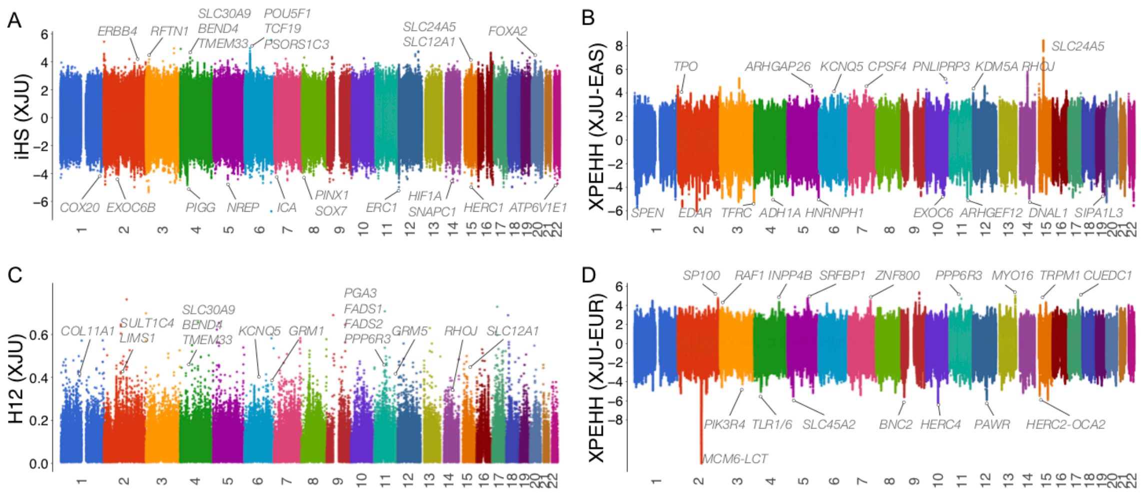

Figure S18 | Genomic signatures of selective sweeps in XJU indicated by haplotype-homozygosity-based methods.

A. Genome-wide normalized iHS values of XJU; **B.** Genome-wide H12 values of XJU; **C.** Genome-wide XP-EHH scores between XJU and EAS; **D.** Genome-wide XP-EHH scores between XJU and EUR.

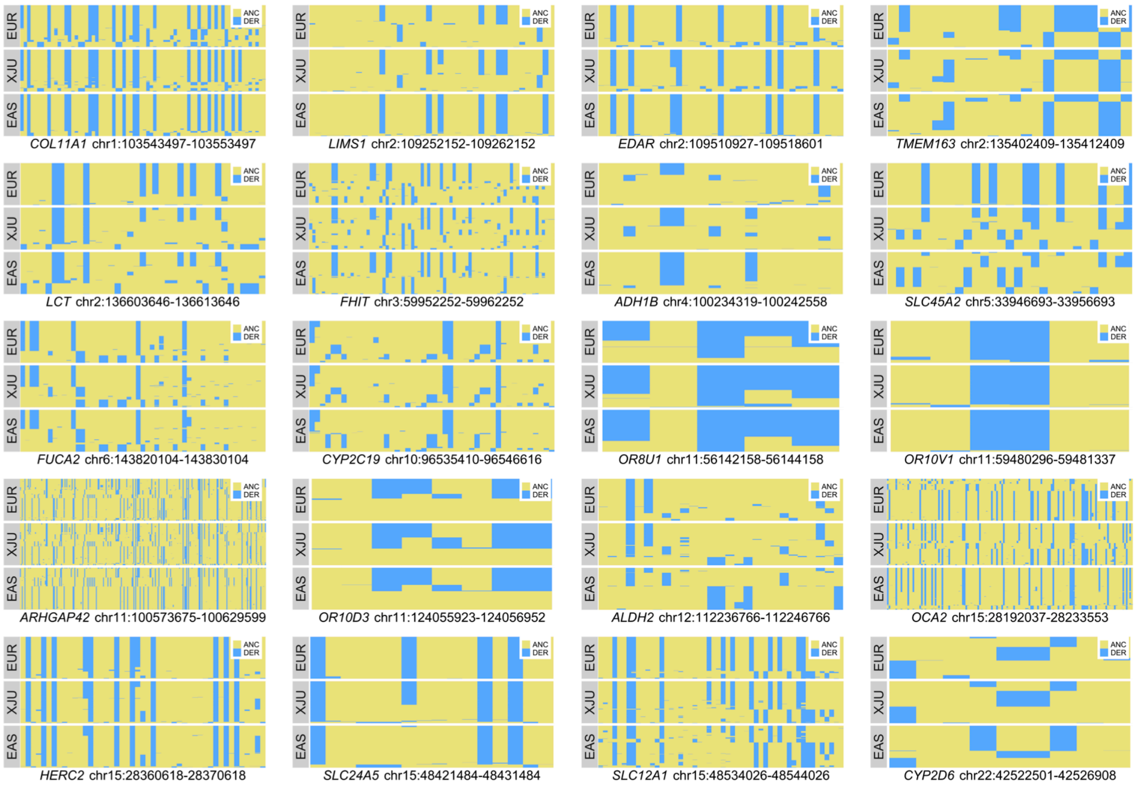

Figure S19 | Haplotype plot for local genes.

Each haplotype was represented by a horizontal line, which was partitioned into segments corresponding to the ancestral states of alleles indicated by color.

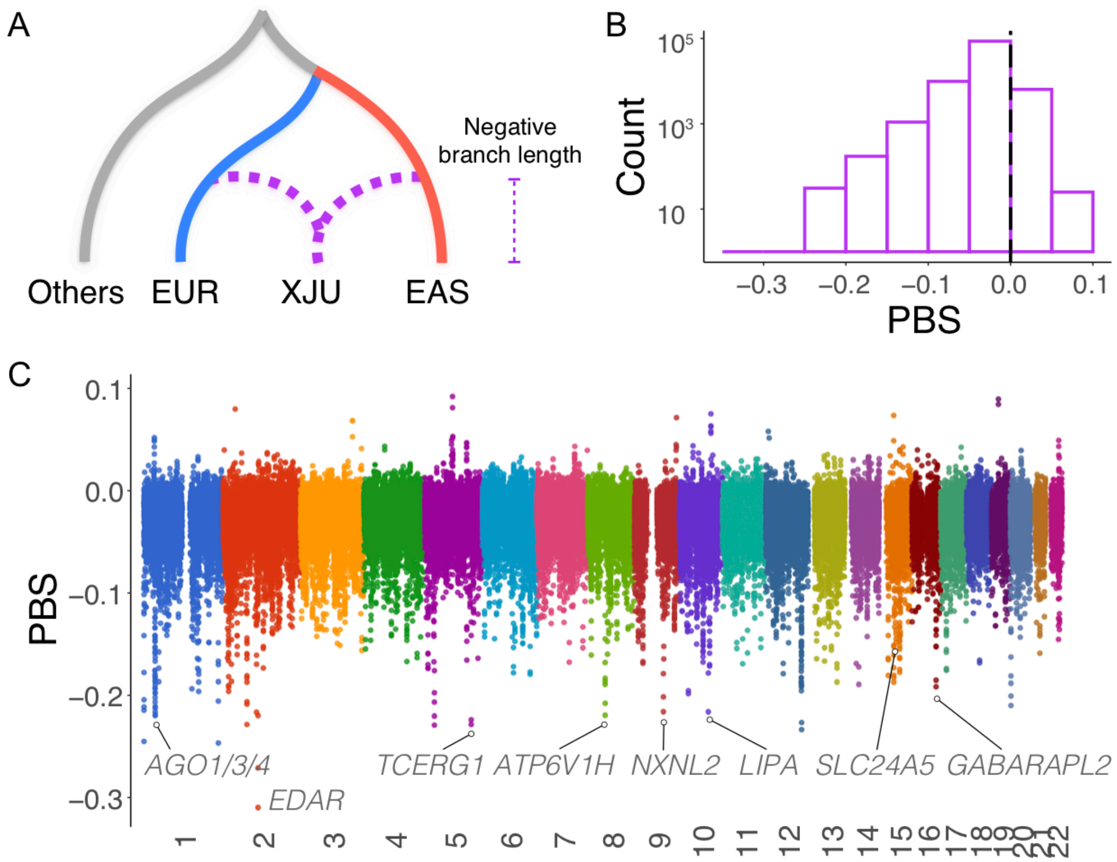

Figure S20 | Identification of admixture-representative (AR) components in XJU.

A. Simple admixture model of XJU. The population-specific branch length of XJU is negative due to admixture; **B.** Histogram for the genome-wide distribution of population-branch statistics (PBS) values. PBS values were estimated within sliding windows across the genome of 50 Kb in length shift by 25 Kb; **C.** Genome-wide PBS values of XJU. Top signals were labeled in the plot.

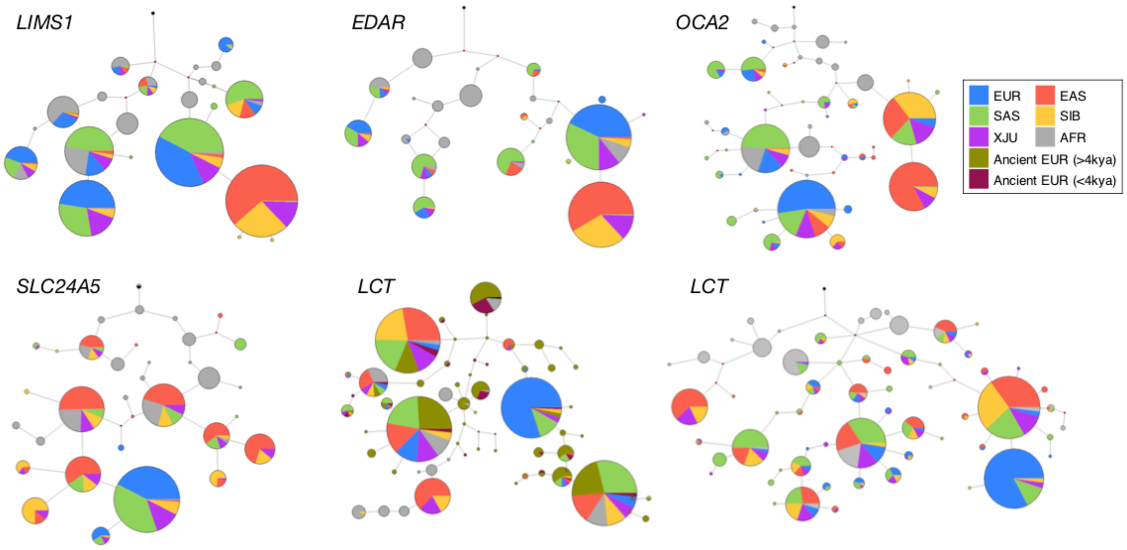

Figure S21 | Haplotype network.

Data set was merged from XJU, KGP, and EGDP data sets, including XJU, EUR (CEU and GBR), SAS (GIH and ITU), EAS (CHB and CDX), SIB, and AFR (YRI) populations. SNVs with AF <0.01 and >0.99 among all of the populations were removed. Haplotypes from ancient EUR populations were also included to investigate the phylogeny of *LCT*.

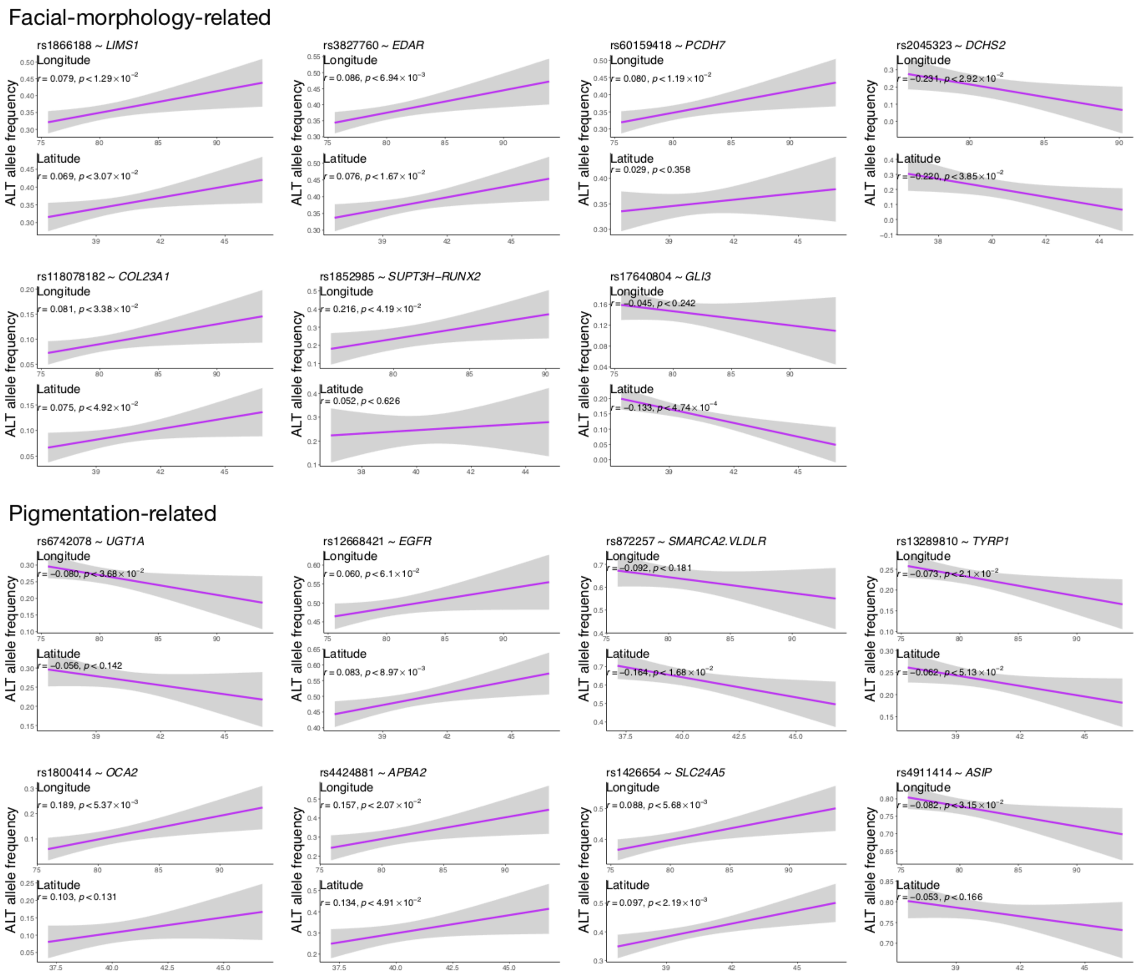

Figure S22 | Association between geographic coordinate and allele frequency of XJU individuals.

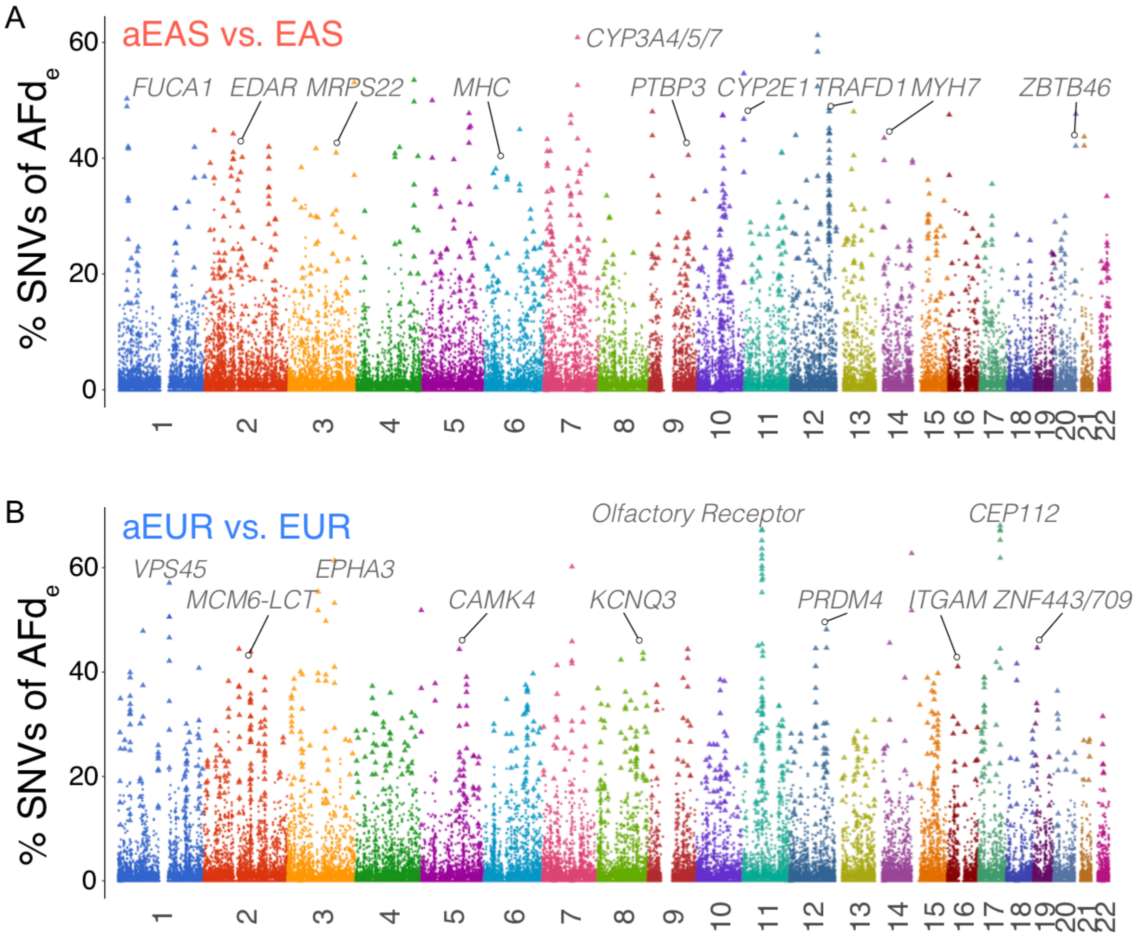

Figure S23 | Genomic signatures of allele frequency deviation between reconstructed ancestral populations and reference populations.

A. Allele frequency deviation from expectation (AFd_e) between reconstructed ancestral EAS population (aEAS) and EAS population. The y-axis indicated the proportions of SNV with high AFd_e within sliding windows of 50 Kb in length shift by 25 Kb across the genome. Outliers were in triangle shape; **B.** AFd_e between reconstructed ancestral EUR population (aEUR) and EUR population.

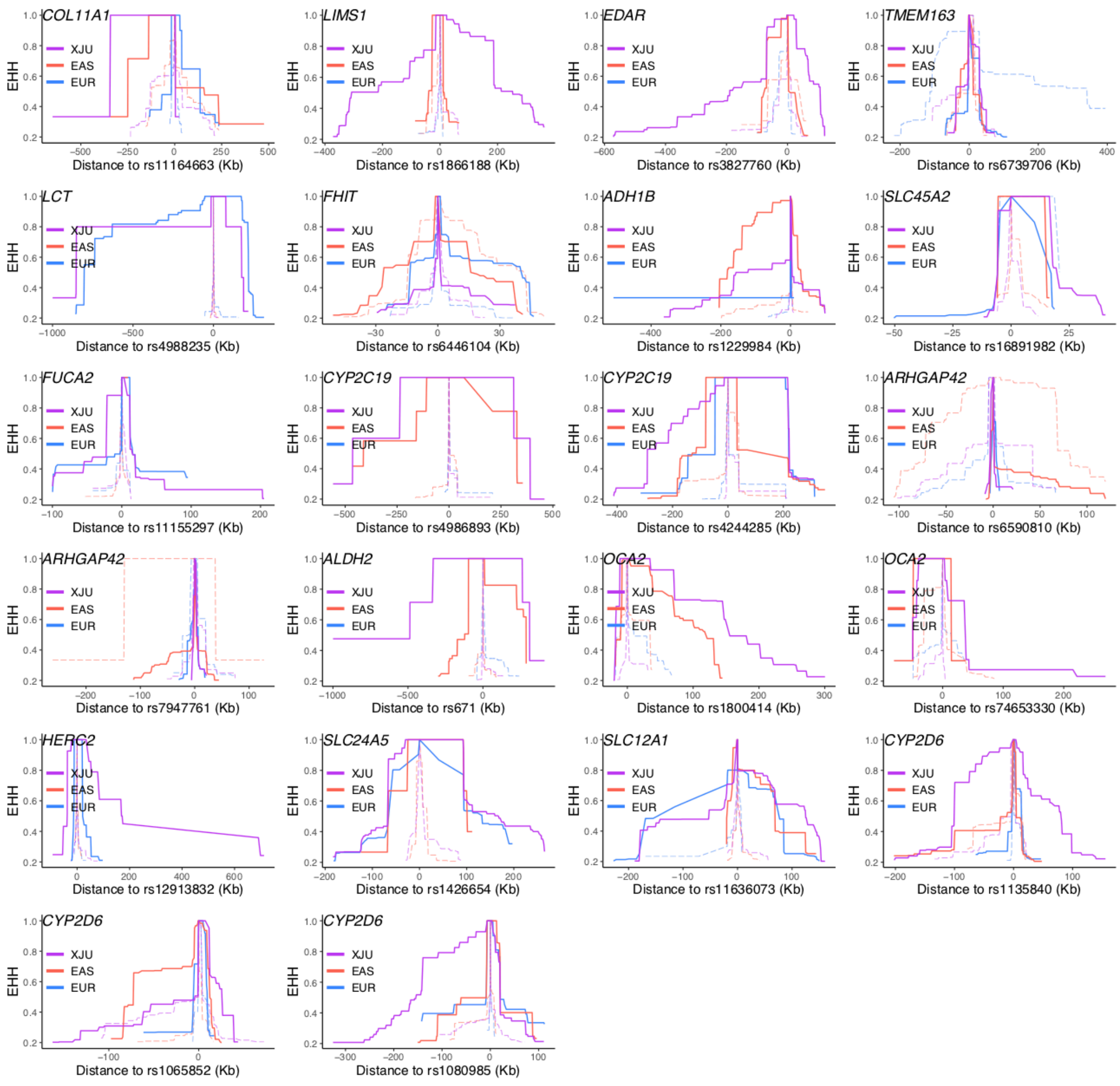

Figure S24 | Extended haplotype homozygosity of local genes.

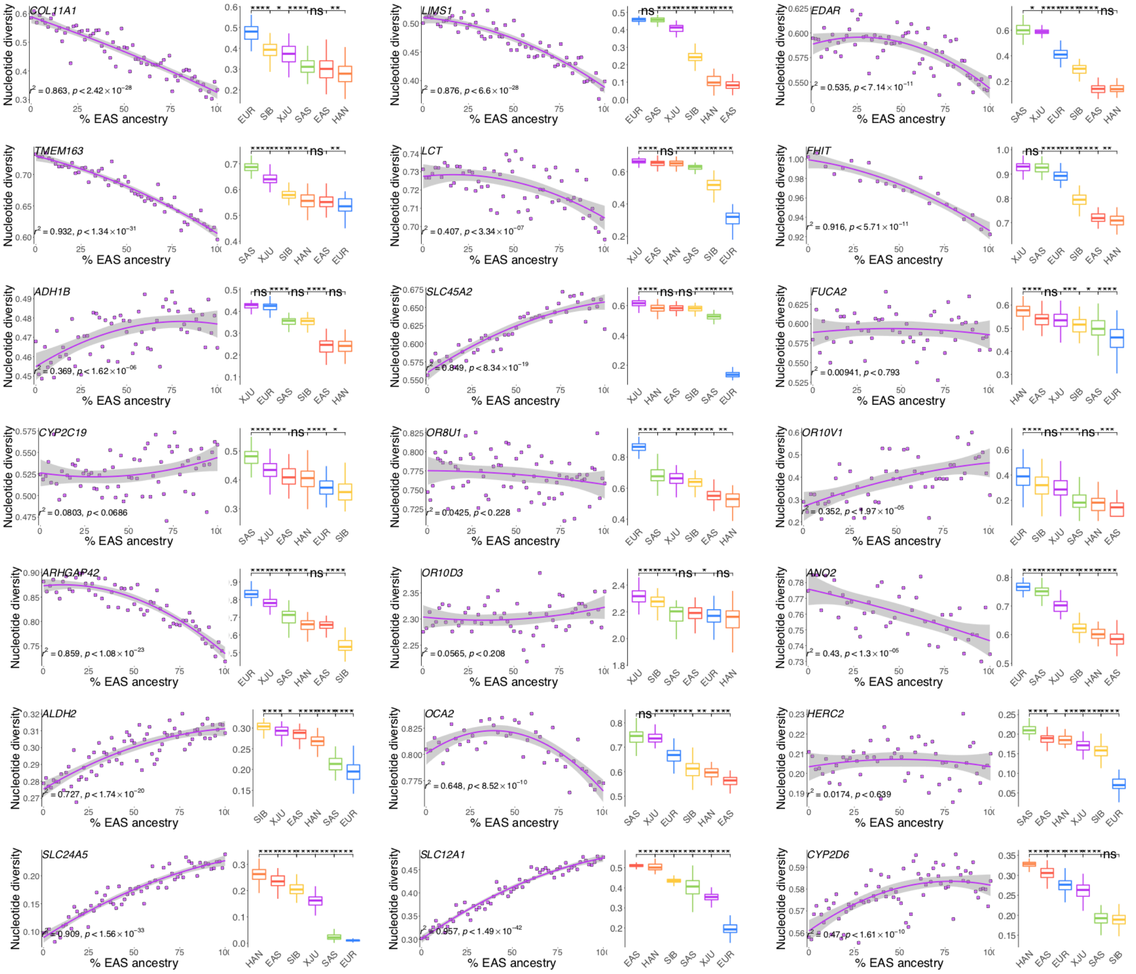

Figure S25 | Genetic diversity of local genes.

Genetic diversity was estimated within the gene region. Five replicates were conducted for XJU under each condition of admixture proportion by random sampling ancestry-specific haplotypes. The curves were fitted using the function "lm" in R. Five replicates were also conducted for the reference populations, with the same number of haplotypes random sampled to balance the sample size.

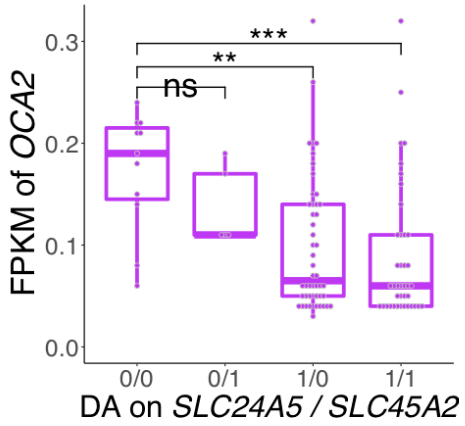

Figure S26 | Expression profiles of *OCA2* in XJU individuals.

XJU individuals were grouped into different classes based on whether carrying derived alleles on *SLC24A5* and *SLC45A2*. "0" indicated no derived allele, and "1" indicated derived allele on the corresponding gene. Statistical significance of Wilcoxon rank sum test: ns: p > 0.05, *: p <= 0.05, **: p <= 0.01, ***: p <= 0.001, ****: p <= 0.0001.

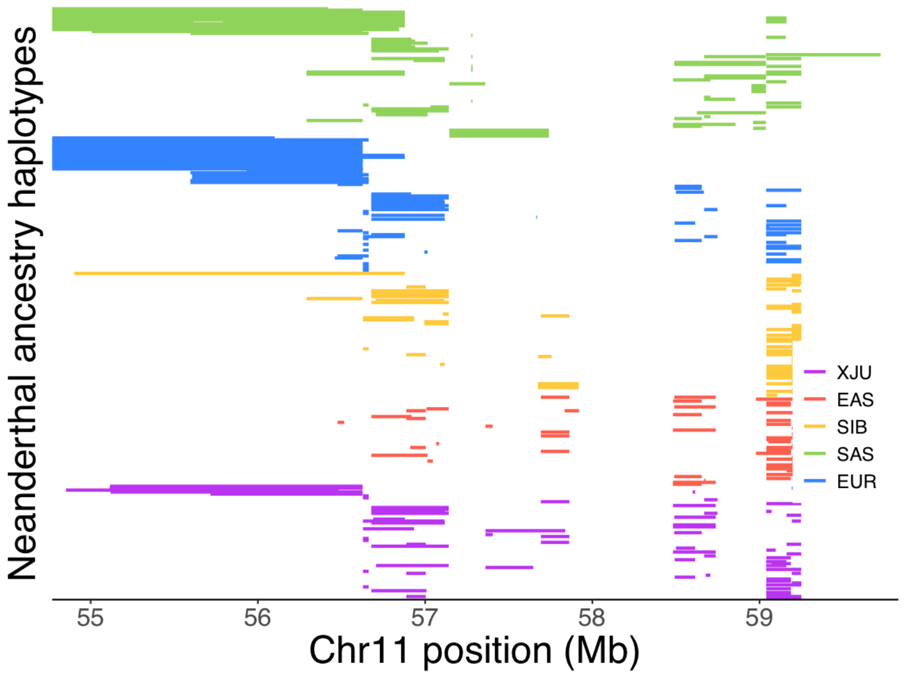

Figure S27 | Neanderthal haplotypes along the region of OR gene cluster across different populations.

Supplementary Tables

Table S1 | Information of the samples for sequencing in this study.

ID	Depth	Mapping Rate (%)	Sex	Long.	Lat.	Region	ID	Depth	Mapping Rate (%)	Sex	Long.	Lat.	Region
WGC021959D	32.97	99.70	M	76.72	39.49	Kaxgar	WGC022005D	35.74	99.29	M	79.18	40.40	Aksu
WGC021960D	32.79	99.46	F	86.38	42.32	Bayingolin	WGC022006D	40.08	99.99	F	77.25	38.41	Kaxgar
WGC021961D	38.39	99.28	F	75.99	39.47	Kaxgar	WGC022007D	34.34	99.70	F	76.18	38.93	Kaxgar
WGC021962D	37.48	99.44	M	75.99	39.47	Kaxgar	WGC022008D	33.53	99.63	F	88.87	43.15	Turpan
WGC021963D	33.07	99.42	M	76.72	39.49	Kaxgar	WGC022009D	33.39	99.51	F	75.99	39.47	Kaxgar
WGC021964D	32.40	99.23	M	78.28	37.62	Hotan	WGC022010D	35.29	99.53	F	79.82	37.12	Kizilsu
WGC021965D	34.66	99.09	M	81.68	36.86	Hotan	WGC022011D	29.45	99.69	F	-	-	-
WGC021966D	36.93	99.02	M	88.65	42.79	Turpan	WGC022012D	36.85	99.68	M	76.17	38.97	Kaxgar
WGC021967D	33.48	99.18	M	82.96	41.72	Aksu	WGC022013D	31.74	98.73	F	75.99	39.47	Kaxgar
WGC021968D	35.71	99.08	M	-	-	-	WGC022014D	41.50	99.17	F	75.99	39.47	Kaxgar
WGC021969D	34.66	99.03	M	76.77	39.22	Kaxgar	WGC022015D	36.29	99.31	F	76.05	39.40	Kaxgar
WGC021970D	34.18	99.17	M	80.37	40.64	Aksu	WGC022016D	29.28	99.80	F	86.15	41.76	Bayingolin
WGC021971D	31.17	99.33	M	77.25	38.41	Kaxgar	WGC022017D	31.57	99.76	F	81.87	41.80	Aksu
WGC021972D	33.23	99.48	F	80.31	41.14	Aksu	WGC022018D	38.48	99.71	F	87.31	44.01	Changji
WGC021973D	37.92	99.52	F	79.82	37.12	Hotan	WGC022019D	30.91	99.79	F	83.02	41.69	Aksu
WGC021974D	36.73	99.49	F	84.25	41.78	Bayingolin	WGC022020D	31.54	99.77	F	75.99	39.47	Kaxgar
WGC021975D	35.52	99.67	F	76.05	39.49	Kaxgar	WGC022021D	30.26	99.78	F	75.99	39.47	Kaxgar
WGC021976D	36.73	99.71	F	80.37	40.64	Aksu	WGC022022D	33.98	99.68	F	76.17	39.71	Kizilsu
WGC021977D	34.77	99.36	F	75.99	39.47	Kaxgar	WGC022023D	37.29	99.77	F	77.23	38.37	Kaxgar
WGC021978D	39.04	99.77	F	77.41	37.88	Kaxgar	WGC022024D	30.48	99.76	F	88.17	39.02	Bayingolin
WGC021979D	39.88	99.59	F	79.82	37.12	Hotan	WGC022025D	35.04	99.66	F	85.53	38.15	Bayingolin
WGC021980D	33.05	99.39	F	79.82	37.12	Hotan	WGC022026D	32.52	99.51	F	75.99	39.47	Kaxgar
WGC021981D	34.00	99.62	M	80.26	41.17	Aksu	WGC022027D	39.82	99.81	F	86.86	42.27	Bayingolin
WGC021982D	32.60	99.64	M	79.82	37.12	Hotan	WGC022028D	34.25	99.47	F	86.38	42.32	Bayingolin
WGC021983D	30.23	99.19	M	80.31	41.14	Aksu	WGC022029D	32.87	99.58	F	89.59	44.02	Changji
WGC021984D	38.18	99.57	F	79.82	37.12	Hotan	WGC022030D	30.73	99.57	M	75.99	39.47	Kaxgar
WGC021985D	39.54	99.56	F	82.93	41.71	Kaxgar	WGC022031D	32.29	99.53	M	76.72	39.49	Kaxgar
WGC021986D	34.49	99.67	F	77.23	38.37	Kaxgar	WGC022032D	32.01	99.50	M	76.17	39.72	Kizilsu
WGC021987D	36.08	99.68	F	89.19	42.95	Turpan	WGC022033D	36.87	99.57	M	75.99	39.47	Kaxgar
WGC021988D	39.25	99.10	F	79.82	37.12	Hotan	WGC022034D	33.94	99.54	M	90.21	42.87	Turpan
WGC021989D	39.64	99.61	F	82.96	41.72	Aksu	WGC022035D	33.76	99.60	M	81.28	43.91	Ili
WGC021990D	35.84	99.68	M	79.82	37.12	Hotan	WGC022036D	33.31	99.39	M	86.17	41.73	Bayingolin
WGC021991D	35.64	99.76	M	76.17	39.71	Kizilsu	WGC022037D	34.27	99.51	M	76.17	39.71	Kizilsu
WGC021992D	35.07	99.38	F	75.98	39.15	Kizilsu	WGC022038D	33.75	99.49	M	90.21	42.87	Turpan
WGC021993D	38.76	99.33	F	77.25	38.41	Kaxgar	WGC022039D	42.36	99.61	M	76.05	39.40	Kaxgar
WGC021994D	36.94	99.51	M	83.02	41.69	Aksu	WGC022040D	34.44	99.70	M	75.86	39.38	Kaxgar
WGC021995D	34.24	99.49	F	88.65	42.79	Turpan	WGC022041D	32.96	99.38	M	80.26	41.17	Aksu
WGC021996D	34.63	99.58	F	89.19	42.95	Turpan	WGC022042D	40.27	99.62	M	-	-	-
WGC021997D	37.80	99.20	F	89.19	42.95	Turpan	WGC022043D	37.40	99.44	M	86.26	41.34	Bayingolin
WGC021998D	41.07	99.26	F	79.82	37.12	Hotan	WGC022044D	32.16	99.77	F	80.26	41.17	Aksu
WGC021999D	34.41	99.28	F	79.82	37.12	Hotan	WGC022045D	34.25	99.73	F	82.05	44.85	Bortala
WGC022000D	34.46	99.38	F	83.02	41.69	Aksu	WGC022046D	33.31	99.76	F	75.86	39.38	Kaxgar
WGC022001D	34.74	99.13	F	90.21	42.87	Turpan	WGC022047D	34.17	99.78	F	82.05	44.85	Bortala
WGC022002D	34.96	99.38	F	79.82	37.12	Hotan	WGC022048D	35.31	99.78	F	82.23	43.48	Ili
WGC022003D	32.40	99.19	F	80.26	41.17	Aksu	WGC022049D	32.95	99.78	F	87.58	43.76	Urumqi
WGC022004D	32.25	99.64	M	79.73	37.28	Hotan	WGC022050D	36.97	99.77	M	86.20	41.75	Bayingolin

Note: The "Long." and "Lat." respectively denote the longitude and latitude of the geographic locations. "M" and "F" indicate "Male" and "Female" in the "Sex" column.

Table S2 | Summary of SNVs discovered from deep whole-genome sequencing.

	Number of SNVs	dbSNP138		dbSNP147		dbSNP153	
		Number of Novel SNVs	Novelty rates of SNVs	Number of Novel SNVs	Novelty rates of SNVs	Number of Novel SNVs	Novelty rates of SNVs
Total	15,171,182	4,665,610	30.8%	3,141,226	20.7%	1,231,219	8.12%
XJU (92)	12,031,412	2,573,707	21.4%	1,572,932	13.1%	668,670	5.56%
HAN (90)	10,204,296	2,264,422	22.2%	1,647,941	16.1%	566,505	5.55%
Mean number of SNVs per sample							
XJU	2,994,099						
HAN	2,943,657						

Note: Number of SNVs were counted for the dataset after the procedures of VQSR and universal mask. The “Total” row indicates the number of SNVs in the joint dataset including XJU and HAN.

Table S3 | SNV counts of different consequence impacts.

Variant type	Total	XJU (92)	HAN (90)
High impact (Loss of function)			
splice_acceptor_variant	907	667	583
splice_donor_variant	1,424	1,059	930
stop_gained	1,556	989	916
stop_lost	217	169	149
start_lost	290	213	179
Moderate impact			
missense_variant	85,582	60,845	51,934
Low impact			
splice_region_variant	17,841	13,769	11,623
incomplete_terminal_codon_variant	6	5	4
stop_retained_variant	94	80	57
synonymous_variant	60,668	46,773	39,484
Modifier			
coding_sequence_variant	7	6	3
mature_miRNA_variant	319	251	217
5_prime_UTR_variant	47,254	37,001	30,782
3_prime_UTR_variant	162,669	125,655	105,367
non_coding_transcript_exon_variant	237,380	187,089	158,432
intron_variant	7,631,152	6,021,182	5,099,683
non_coding_transcript_variant	1	1	1
upstream_gene_variant	690,272	552,105	468,951
downstream_gene_variant	571,334	456,834	387,687
intergenic_variant	5,662,209	4,526,719	3,847,314
Total	15,171,182	12,031,412	10,204,296

Note: The “Total” column indicates the number of SNVs in the joint dataset including XJU and HAN.

Table S4 | Pathways of higher genetic burden in XJU individuals.

Pathway	Super pathway (level 1)	Super pathway (level 2)	# Deleterious SNVs (median)			BH-corrected *P*-value (Wilcoxon rank sum test)		
			XJU	EAS	EUR	XJU vs. EAS	XJU vs. EUR	EAS vs. EUR
**Genetic burden estimated as counts of LoF and missense variants under the dominant model**								
Metabolism	Metabolism	Metabolism	745.5	732	721	2.62E-04	2.89E-08	1.64E-02
Global and overview maps	Metabolism	Global and overview maps	649	640	632	5.15E-03	3.95E-07	9.79E-03
Metabolic pathways	Metabolism	Global and overview maps	645	636	629	1.91E-03	1.33E-06	5.54E-02
Metabolism of other amino acids	Metabolism	Metabolism of other amino acids	63	58	61	4.25E-07	4.89E-02	7.94E-05
Folding, sorting and degradation	Genetic Information Processing	Folding, sorting and degradation	145.5	138	141	5.41E-07	8.88E-03	4.90E-03
Nucleotide excision repair	Genetic Information Processing	Replication and repair	14	13	12	8.46E-03	2.74E-03	6.99E-01
Environmental Information Processing	Environmental Information Processing	Environmental Information Processing	984	962	948	1.66E-06	5.84E-12	2.12E-04
Signal transduction	Environmental Information Processing	Signal transduction	684.5	666	666	6.76E-09	1.82E-08	8.78E-01
Calcium signaling pathway	Environmental Information Processing	Signal transduction	70.5	68	68	3.81E-03	1.36E-03	7.14E-01
FoxO signaling pathway	Environmental Information Processing	Signal transduction	38	36	36	2.07E-02	1.32E-02	9.47E-01
Signaling molecules and interaction	Environmental Information Processing	Signaling molecules and interaction	458.5	452	437	4.59E-02	8.97E-09	2.85E-06
ECM-receptor interaction	Environmental Information Processing	Signaling molecules and interaction	142	136	137	3.38E-04	1.51E-04	8.01E-01
Cell growth and death	Cellular Processes	Cell growth and death	201	196	196	6.08E-03	9.09E-04	6.03E-01
Cell motility	Cellular Processes	Cell motility	86.5	84	84	5.15E-03	4.97E-04	6.64E-01
Regulation of actin cytoskeleton	Cellular Processes	Cell motility	86.5	84	84	5.15E-03	4.97E-04	6.64E-01
Organismal Systems	Organismal Systems	Organismal Systems	1624	1599	1580	2.41E-06	3.95E-10	6.73E-03
Immune system	Organismal Systems	Immune system	470.5	465	444	4.35E-02	2.63E-15	4.86E-13
Chemokine signaling pathway	Organismal Systems	Immune system	39	37	37	1.09E-02	6.93E-03	9.52E-01
Complement and coagulation cascades	Organismal Systems	Immune system	63	61	59	4.35E-02	1.45E-06	7.60E-03
Adrenergic signaling in cardiomyocytes	Organismal Systems	Circulatory system	38.5	37	36	2.63E-02	4.10E-03	4.02E-01
Digestive system	Organismal Systems	Digestive system	260.5	256	251	1.64E-02	1.45E-06	5.32E-03
Synaptic vesicle cycle	Organismal Systems	Nervous system	30.5	29	29	3.95E-02	3.53E-02	9.15E-01
Human Diseases	Human Diseases	Human Diseases	1409.5	1376	1360	1.59E-06	1.96E-12	1.14E-04
Cancer: overview	Human Diseases	Cancer: overview	449	439	437	1.25E-05	2.25E-05	6.65E-01
Pathways in cancer	Human Diseases	Cancer: overview	216	211	210	1.97E-02	5.93E-03	6.29E-01
Viral carcinogenesis	Human Diseases	Cancer: overview	58	55	56	9.48E-05	1.42E-02	2.61E-01
Drug resistance: antineoplastic	Human Diseases	Drug resistance: antineoplastic	90	87	89	8.42E-05	2.42E-02	6.90E-02
Neurodegenerative disease	Human Diseases	Neurodegenerative disease	168	162	164	3.56E-04	4.19E-02	1.00E-01
Huntington disease	Human Diseases	Neurodegenerative disease	113	108	109	2.09E-03	5.10E-03	5.70E-01
Infectious disease: viral	Human Diseases	Infectious disease: viral	692.5	674	664	6.50E-05	8.97E-09	1.05E-02
Human papillomavirus infection	Human Diseases	Infectious disease: viral	191	186	183	2.65E-03	4.12E-06	6.42E-02
**Genetic burden estimated as counts of variants with CADD score >15 under the dominant model**								
Metabolism	Metabolism	Metabolism	949.5	922	912	2.16E-07	6.44E-12	6.25E-03
Global and overview maps	Metabolism	Global and overview maps	833.5	814	795	1.36E-04	1.74E-12	2.84E-07
Metabolic pathways	Metabolism	Global and overview maps	831	812	794	8.75E-05	3.27E-12	1.42E-06
Energy metabolism	Metabolism	Energy metabolism	37	34	35	1.91E-04	2.44E-03	3.77E-01
Oxidative phosphorylation	Metabolism	Energy metabolism	24	23	23	1.68E-02	1.86E-02	9.69E-01
Purine metabolism	Metabolism	Nucleotide metabolism	148	142	145	1.24E-07	2.18E-02	5.89E-04
Amino acid metabolism	Metabolism	Amino acid metabolism	137	132	128	3.65E-03	1.25E-06	1.07E-02
Transcription	Genetic Information Processing	Transcription	53	51	49	3.48E-03	1.72E-04	2.51E-01
Protein processing in endoplasmic reticulum	Genetic Information Processing	Folding, sorting and degradation	72	67	69	3.81E-04	1.51E-02	3.73E-01
Environmental Information Processing	Environmental Information Processing	Environmental Information Processing	2050	1989	1996	9.01E-16	8.55E-12	3.94E-01
Signal transduction	Environmental Information Processing	Signal transduction	1658	1602	1622	2.81E-16	5.09E-07	1.75E-04
cGMP-PKG signaling pathway	Environmental Information Processing	Signal transduction	208.5	203	204	5.26E-05	6.02E-04	8.19E-01
Sphingolipid signaling pathway	Environmental Information Processing	Signal transduction	92	89	89	1.02E-02	3.07E-02	8.12E-01
AMPK signaling pathway	Environmental Information	Signal transduction	110	106	106	1.14E-03	3.39E-04	5.42E-01

Pathway	Super pathway (level 1)	Super pathway (level 2)	# Deleterious SNVs (median)			BH-corrected *P*-value (Wilcoxon rank sum test)		
			XJU	EAS	EUR	XJU vs. EAS	XJU vs. EUR	EAS vs. EUR
	Processing							
Hippo signaling pathway	Environmental Information Processing	Signal transduction	188	182	183	1.49E-03	2.61E-02	4.76E-01
Signaling molecules and interaction	Environmental Information Processing	Signaling molecules and interaction	658.5	636	638	1.45E-07	1.21E-06	9.24E-01
Cytokine-cytokine receptor interaction	Environmental Information Processing	Signaling molecules and interaction	76.5	72	75	1.71E-05	4.34E-02	3.37E-02
Cellular Processes	Cellular Processes	Cellular Processes	1202	1176	1179	6.15E-10	1.57E-07	4.64E-01
Cellular community - eukaryotes	Cellular Processes	Cellular community - eukaryotes	661	647	639	1.26E-05	2.21E-08	1.26E-01
Adherens junction	Cellular Processes	Cellular community - eukaryotes	143	138	137	1.71E-03	1.39E-03	8.69E-01
Organismal Systems	Organismal Systems	Organismal Systems	2183	2100	2142	5.63E-18	9.90E-07	2.37E-09
Immune system	Organismal Systems	Immune system	586	562	571	2.29E-12	1.62E-06	2.19E-02
Platelet activation	Organismal Systems	Immune system	108.5	107	106	8.37E-03	5.77E-04	4.10E-01
NOD-like receptor signaling pathway	Organismal Systems	Immune system	82	79	81	6.37E-04	2.26E-02	5.71E-01
Th17 cell differentiation	Organismal Systems	Immune system	81.5	77	79	2.97E-04	4.68E-02	1.41E-01
Fc epsilon RI signaling pathway	Organismal Systems	Immune system	48	46	45	3.94E-02	5.71E-03	3.35E-01
Leukocyte transendothelial migration	Organismal Systems	Immune system	130	124	126	2.03E-03	3.68E-03	8.67E-01
Thermogenesis	Organismal Systems	Environmental adaptation	126.5	122	123	3.03E-05	1.48E-02	1.98E-01
Endocrine system	Organismal Systems	Endocrine system	787	760	769	6.97E-10	8.99E-06	3.92E-02
Insulin secretion	Organismal Systems	Endocrine system	141	136	135	1.82E-02	1.93E-03	2.66E-01
Estrogen signaling pathway	Organismal Systems	Endocrine system	156.5	152	152	3.32E-03	3.34E-02	5.95E-01
Oxytocin signaling pathway	Organismal Systems	Endocrine system	200.5	192	197	1.42E-06	3.89E-02	4.51E-04
Circulatory system	Organismal Systems	Circulatory system	306.5	298	292	2.11E-04	6.19E-10	1.35E-03
Adrenergic signaling in cardiomyocytes	Organismal Systems	Circulatory system	192	184	184	2.57E-04	9.12E-05	8.12E-01
Vascular smooth muscle contraction	Organismal Systems	Circulatory system	154	152	146	4.51E-02	1.14E-05	7.08E-03
Digestive system	Organismal Systems	Digestive system	311	307	302	1.57E-02	1.03E-03	1.90E-01
Pancreatic secretion	Organismal Systems	Digestive system	104.5	101	96	1.14E-02	4.46E-08	1.33E-04
Excretory system	Organismal Systems	Excretory system	141	136	135	4.29E-03	2.85E-03	6.32E-01
Synaptic vesicle cycle	Organismal Systems	Nervous system	51	48	48	2.79E-02	3.89E-02	8.49E-01
Axon guidance	Organismal Systems	Development and regeneration	369.5	360	361	2.64E-04	2.34E-02	3.70E-01
Aging	Organismal Systems	Aging	89	83	85	2.75E-04	1.57E-02	3.47E-01
Longevity regulating pathway	Organismal Systems	Aging	85	80	81	3.02E-04	3.12E-02	1.90E-01
Longevity regulating pathway - multiple species	Organismal Systems	Aging	42	39	39	2.11E-02	5.37E-03	5.06E-01
Human Diseases	Human Diseases	Human Diseases	2190	2132	2137	2.13E-16	6.06E-13	3.95E-01
Cancer: overview	Human Diseases	Cancer: overview	1090	1055	1056	9.67E-10	2.65E-09	9.11E-01
Pathways in cancer	Human Diseases	Cancer: overview	533.5	517	515	5.93E-07	8.99E-06	9.43E-01
Transcriptional misregulation in cancer	Human Diseases	Cancer: overview	218.5	210	215	1.23E-04	2.84E-02	7.34E-02
Viral carcinogenesis	Human Diseases	Cancer: overview	130	125	127	5.36E-03	4.95E-02	5.13E-01
Cancer: specific types	Human Diseases	Cancer: specific types	608	589	588	2.54E-07	3.17E-06	9.55E-01
Prostate cancer	Human Diseases	Cancer: specific types	104	100	100	6.71E-04	3.68E-03	8.43E-01
Cardiovascular disease	Human Diseases	Cardiovascular disease	348	335	340	1.68E-06	2.75E-03	6.54E-02
Arrhythmogenic right ventricular cardiomyopathy (ARVC)	Human Diseases	Cardiovascular disease	184.5	177	179	1.47E-04	3.31E-03	3.23E-01
Endocrine and metabolic disease	Human Diseases	Endocrine and metabolic disease	356	348	349	1.77E-03	6.02E-04	8.24E-01
Insulin resistance	Human Diseases	Endocrine and metabolic disease	99	94	96	1.39E-02	3.38E-02	6.98E-01
Infectious disease: viral	Human Diseases	Infectious disease: viral	654.5	641	636	1.23E-04	8.99E-06	3.07E-01
Human papillomavirus infection	Human Diseases	Infectious disease: viral	301	296	295	2.73E-02	4.40E-03	3.18E-01
Human T-cell leukemia virus 1 infection	Human Diseases	Infectious disease: viral	131	126	128	3.82E-03	4.34E-02	3.96E-01
**Genetic burden estimated as counts of variants with GERP score >2 under the dominant model**								
Metabolism	Metabolism	Metabolism	4583	4506	4416	1.45E-10	5.49E-22	4.53E-11
Global and overview maps	Metabolism	Global and overview maps	4079.5	4017	3919	1.85E-06	1.16E-21	2.65E-15
Metabolic pathways	Metabolism	Global and overview maps	4076	4012	3915	1.61E-06	1.16E-21	4.57E-15
Carbon metabolism	Metabolism	Global and overview maps	214	207	206	2.62E-02	2.52E-04	1.23E-01
Energy metabolism	Metabolism	Energy metabolism	156	152	150	3.66E-02	4.58E-03	4.50E-01
Lipid metabolism	Metabolism	Lipid metabolism	816.5	799	786	1.15E-03	1.56E-07	5.58E-03
Fatty acid elongation	Metabolism	Lipid metabolism	52	49	50	1.88E-03	2.98E-02	4.54E-01
Steroid hormone biosynthesis	Metabolism	Lipid metabolism	73.5	69	71	5.35E-04	2.21E-02	2.35E-01

Pathway	Super pathway (level 1)	Super pathway (level 2)	# Deleterious SNVs (median)			BH-corrected *P*-value (Wilcoxon rank sum test)		
			XJU	EAS	EUR	XJU vs. EAS	XJU vs. EUR	EAS vs. EUR
Arachidonic acid metabolism	Metabolism	Lipid metabolism	76	72	72	3.24E-04	1.86E-02	4.06E-01
Linoleic acid metabolism	Metabolism	Lipid metabolism	46	42	43	5.15E-04	4.07E-02	3.11E-01
alpha-Linolenic acid metabolism	Metabolism	Lipid metabolism	45	41	42	1.19E-03	4.37E-02	4.68E-01
Nucleotide metabolism	Metabolism	Nucleotide metabolism	885.5	852	856	5.97E-10	2.72E-08	4.11E-01
Purine metabolism	Metabolism	Nucleotide metabolism	789.5	753	766	7.07E-10	9.42E-08	1.26E-01
Amino acid metabolism	Metabolism	Amino acid metabolism	593	572	575	1.36E-07	5.65E-06	5.01E-01
Cysteine and methionine metabolism	Metabolism	Amino acid metabolism	77	74	73	4.64E-03	1.54E-03	5.24E-01
Selenocompound metabolism	Metabolism	Metabolism of other amino acids	36	34	34	2.27E-03	1.34E-02	5.85E-01
Glycan biosynthesis and metabolism	Metabolism	Glycan biosynthesis and metabolism	1065.5	1042	1038	1.80E-06	1.18E-06	6.81E-01
Glycosphingolipid biosynthesis - lacto and neolacto series	Metabolism	Glycan biosynthesis and metabolism	83.5	81	79	3.99E-02	5.67E-04	6.64E-02
Ubiquinone and other terpenoid-quinone biosynthesis	Metabolism	Metabolism of cofactors and vitamins	12	11	10	2.04E-02	5.24E-05	5.34E-02
One carbon pool by folate	Metabolism	Metabolism of cofactors and vitamins	49	45	46	1.39E-05	7.14E-03	7.28E-02
Thiamine metabolism	Metabolism	Metabolism of cofactors and vitamins	45.5	43	42	4.13E-02	3.77E-02	8.37E-01
Genetic Information Processing	Genetic Information Processing	Genetic Information Processing	2139	2046	2096	1.33E-18	5.10E-06	2.56E-09
Translation	Genetic Information Processing	Translation	713.5	693	696	1.47E-07	2.64E-04	3.88E-01
Ribosome	Genetic Information Processing	Translation	68	64	64	6.83E-04	1.15E-04	7.67E-01
RNA transport	Genetic Information Processing	Translation	217.5	212	211	3.49E-02	1.13E-02	6.08E-01
Folding, sorting and degradation	Genetic Information Processing	Folding, sorting and degradation	919	888	902	1.69E-09	7.59E-03	7.64E-04
Ubiquitin mediated proteolysis	Genetic Information Processing	Folding, sorting and degradation	292.5	282	283	2.25E-03	2.93E-02	4.38E-01
Environmental Information Processing	Environmental Information Processing	Environmental Information Processing	9916	9650	9663	1.15E-23	2.38E-21	6.02E-01
Signal transduction	Environmental Information Processing	Signal transduction	7932.5	7690	7728	1.04E-21	1.35E-19	2.01E-01
MAPK signaling pathway	Environmental Information Processing	Signal transduction	1436.5	1386	1408	1.54E-12	1.43E-06	5.33E-04
Calcium signaling pathway	Environmental Information Processing	Signal transduction	1292.5	1258	1284	8.46E-12	3.36E-02	6.06E-08
cGMP-PKG signaling pathway	Environmental Information Processing	Signal transduction	1039	1002	1007	1.47E-10	2.19E-08	5.85E-01
HIF-1 signaling pathway	Environmental Information Processing	Signal transduction	389	382	385	2.69E-03	8.68E-03	6.47E-01
FoxO signaling pathway	Environmental Information Processing	Signal transduction	332.5	326	324	9.50E-03	3.77E-04	2.28E-01
Phospholipase D signaling pathway	Environmental Information Processing	Signal transduction	875	856	828	9.85E-03	8.21E-13	6.30E-08
mTOR signaling pathway	Environmental Information Processing	Signal transduction	393	385	379	8.66E-03	1.60E-03	4.87E-01
PI3K-Akt signaling pathway	Environmental Information Processing	Signal transduction	1725	1651	1705	5.93E-17	3.78E-03	3.06E-13
AMPK signaling pathway	Environmental Information Processing	Signal transduction	453	439	427	3.78E-05	4.49E-08	2.99E-02
TGF-beta signaling pathway	Environmental Information Processing	Signal transduction	317	305	307	5.00E-03	1.05E-02	5.41E-01
Apelin signaling pathway	Environmental Information Processing	Signal transduction	757	735	712	2.33E-06	2.92E-16	2.33E-07
Signaling molecules and interaction	Environmental Information Processing	Signaling molecules and interaction	3293	3193	3242	1.04E-17	1.17E-07	2.81E-06
Cytokine-cytokine receptor interaction	Environmental Information Processing	Signaling molecules and interaction	371	356	355	8.89E-09	1.90E-08	9.24E-01
Viral protein interaction with cytokine and cytokine receptor	Environmental Information Processing	Signaling molecules and interaction	72	69	69	1.75E-02	2.82E-04	1.59E-01
ECM-receptor interaction	Environmental Information Processing	Signaling molecules and interaction	675	657	664	9.29E-06	1.09E-02	5.48E-02
Cell adhesion molecules (CAMs)	Environmental Information Processing	Signaling molecules and interaction	1138	1121	1096	1.35E-02	1.83E-07	9.56E-04
Cellular Processes	Cellular Processes	Cellular Processes	5398	5298	5230	1.46E-13	5.46E-21	1.33E-07
Transport and catabolism	Cellular Processes	Transport and catabolism	1835	1811	1796	1.24E-03	2.72E-08	2.90E-03
Cell growth and death	Cellular Processes	Cell growth and death	1113	1076	1088	1.65E-10	1.94E-07	1.42E-01

Pathway	Super pathway (level 1)	Super pathway (level 2)	# Deleterious SNVs (median)			BH-corrected *P*-value (Wilcoxon rank sum test)		
			XJU	EAS	EUR	XJU vs. EAS	XJU vs. EUR	EAS vs. EUR
Cell cycle	Cellular Processes	Cell growth and death	200.5	186	193	6.24E-07	4.61E-02	9.99E-04
Oocyte meiosis	Cellular Processes	Cell growth and death	352.5	347	331	4.47E-02	4.82E-07	7.70E-04
Cellular senescence	Cellular Processes	Cell growth and death	432.5	421	420	8.80E-04	9.13E-05	7.12E-01
Cellular community - eukaryotes	Cellular Processes	Cellular community - eukaryotes	2994.5	2953	2875	1.47E-05	6.79E-21	1.93E-13
Focal adhesion	Cellular Processes	Cellular community - eukaryotes	1078.5	1062	1052	1.16E-04	4.19E-07	7.96E-02
Adherens junction	Cellular Processes	Cellular community - eukaryotes	597	584	559	3.64E-02	5.00E-12	7.57E-07
Tight junction	Cellular Processes	Cellular community - eukaryotes	799	779	778	4.39E-05	7.27E-07	2.74E-01
Gap junction	Cellular Processes	Cellular community - eukaryotes	596.5	584	577	4.64E-03	2.77E-06	8.39E-02
Organismal Systems	Organismal Systems	Organismal Systems	10165.5	9878	9904	1.45E-24	1.66E-21	3.91E-01
Immune system	Organismal Systems	Immune system	2815.5	2747	2704	6.22E-12	2.34E-21	1.06E-06
Chemokine signaling pathway	Organismal Systems	Immune system	587	574	568	1.42E-03	4.01E-04	6.43E-01
Platelet activation	Organismal Systems	Immune system	616	608	596	2.65E-03	3.62E-06	1.10E-01
NOD-like receptor signaling pathway	Organismal Systems	Immune system	386	377	365	2.38E-02	1.11E-08	2.04E-05
C-type lectin receptor signaling pathway	Organismal Systems	Immune system	290.5	286	282	1.03E-02	1.49E-03	6.18E-01
Hematopoietic cell lineage	Organismal Systems	Immune system	176	171	170	3.42E-02	9.53E-03	6.29E-01
Fc gamma R-mediated phagocytosis	Organismal Systems	Immune system	398.5	385	389	6.79E-06	2.30E-03	2.03E-01
Leukocyte transendothelial migration	Organismal Systems	Immune system	574	556	543	6.26E-03	2.52E-08	7.37E-03
Environmental adaptation	Organismal Systems	Environmental adaptation	1420	1366	1391	2.06E-14	9.46E-06	2.03E-04
Circadian rhythm	Organismal Systems	Environmental adaptation	187.5	180	184	1.28E-05	2.22E-03	1.87E-01
Circadian entrainment	Organismal Systems	Environmental adaptation	825.5	805	821	2.07E-07	1.91E-02	7.27E-04
Thermogenesis	Organismal Systems	Environmental adaptation	596.5	570	579	5.19E-08	5.08E-04	4.46E-02
Endocrine system	Organismal Systems	Endocrine system	3682	3532	3605	1.45E-24	6.41E-10	6.48E-13
Insulin signaling pathway	Organismal Systems	Endocrine system	335.5	330	321	2.16E-02	4.09E-09	9.88E-05
Insulin secretion	Organismal Systems	Endocrine system	689.5	663	677	1.93E-09	7.90E-03	8.90E-04
GnRH signaling pathway	Organismal Systems	Endocrine system	533.5	520	519	9.66E-04	3.26E-03	8.76E-01
Estrogen signaling pathway	Organismal Systems	Endocrine system	646	634	633	1.83E-04	1.36E-05	5.18E-01
Thyroid hormone synthesis	Organismal Systems	Endocrine system	452	434	439	2.43E-06	8.58E-03	2.81E-02
Thyroid hormone signaling pathway	Organismal Systems	Endocrine system	499.5	487	490	4.28E-03	1.88E-03	8.94E-01
Oxytocin signaling pathway	Organismal Systems	Endocrine system	979.5	962	934	1.57E-03	2.23E-15	2.53E-07
Glucagon signaling pathway	Organismal Systems	Endocrine system	430	419	410	5.15E-04	1.65E-07	8.11E-02
Renin secretion	Organismal Systems	Endocrine system	477.5	462	455	3.88E-05	3.63E-10	8.80E-02
Aldosterone synthesis and secretion	Organismal Systems	Endocrine system	675	653	649	6.65E-09	2.44E-09	6.01E-01
Relaxin signaling pathway	Organismal Systems	Endocrine system	625	613	609	1.55E-02	1.72E-04	1.54E-01
Cortisol synthesis and secretion	Organismal Systems	Endocrine system	453	433	442	1.56E-07	6.74E-04	1.58E-02
Circulatory system	Organismal Systems	Circulatory system	1517	1481	1449	2.85E-07	5.98E-18	4.29E-07
Vascular smooth muscle contraction	Organismal Systems	Circulatory system	829.5	802	791	1.51E-08	1.17E-11	1.37E-01
Digestive system	Organismal Systems	Digestive system	1730	1707	1675	2.30E-04	1.56E-14	5.53E-07
Salivary secretion	Organismal Systems	Digestive system	632	620	609	3.60E-03	1.31E-10	1.27E-04
Gastric acid secretion	Organismal Systems	Digestive system	462.5	456	452	2.22E-03	5.74E-05	3.27E-01
Pancreatic secretion	Organismal Systems	Digestive system	570.5	563	537	3.05E-02	7.38E-17	3.06E-13
Bile secretion	Organismal Systems	Digestive system	218.5	214	201	4.82E-02	8.96E-11	3.75E-07
Proximal tubule bicarbonate reclamation	Organismal Systems	Excretory system	45.5	43	39	4.46E-02	4.75E-10	1.84E-06
Nervous system	Organismal Systems	Nervous system	2673	2602	2614	1.65E-10	1.24E-08	2.42E-01
Long-term potentiation	Organismal Systems	Nervous system	521.5	499	510	1.17E-06	8.58E-03	9.75E-03
Retrograde endocannabinoid signaling	Organismal Systems	Nervous system	764.5	742	746	2.45E-06	1.32E-04	3.19E-01
Glutamatergic synapse	Organismal Systems	Nervous system	1031.5	995	1004	1.00E-06	5.70E-06	5.48E-01
Cholinergic synapse	Organismal Systems	Nervous system	741	722	727	4.34E-06	1.33E-04	5.24E-01
Serotonergic synapse	Organismal Systems	Nervous system	591	584	563	2.22E-02	8.01E-10	1.45E-05
GABAergic synapse	Organismal Systems	Nervous system	513	506	495	2.88E-02	2.77E-06	4.80E-03
Dopaminergic synapse	Organismal Systems	Nervous system	837	810	814	3.22E-07	6.71E-05	3.55E-01
Sensory system	Organismal Systems	Sensory system	1406.5	1370	1372	3.22E-05	3.03E-06	7.27E-01
Taste transduction	Organismal Systems	Sensory system	423	412	412	2.10E-04	1.78E-03	6.18E-01
Inflammatory mediator regulation of TRP channels	Organismal Systems	Sensory system	683	668	652	1.20E-03	7.41E-12	2.82E-05
Development and regeneration	Organismal Systems	Development and regeneration	1928.5	1884	1903	1.32E-07	1.08E-03	4.95E-02
Axon guidance	Organismal Systems	Development and regeneration	1684	1643	1660	1.22E-07	5.14E-03	5.64E-03
Human Diseases	Human Diseases	Human Diseases	9412.5	9117	9149	2.64E-25	2.76E-22	1.70E-01
Cancer: overview	Human Diseases	Cancer: overview	4513	4368	4378	8.89E-19	1.75E-16	4.20E-01
Pathways in cancer	Human Diseases	Cancer: overview	2317	2274	2238	1.95E-07	3.20E-16	2.43E-06

Pathway	Super pathway (level 1)	Super pathway (level 2)	# Deleterious SNVs (median)			BH-corrected *P*-value (Wilcoxon rank sum test)		
			XJU	EAS	EUR	XJU vs. EAS	XJU vs. EUR	EAS vs. EUR
Transcriptional misregulation in cancer	Human Diseases	Cancer: overview	739	709	716	2.06E-05	1.41E-03	2.60E-01
Amoebiasis	Human Diseases	Infectious disease: parasitic	448.5	438	437	3.34E-05	5.09E-04	7.33E-01
Drug resistance: antineoplastic	Human Diseases	Drug resistance: antineoplastic	775.5	762	759	1.86E-03	5.59E-04	7.56E-01
Cancer: specific types	Human Diseases	Cancer: specific types	2461.5	2401	2382	1.54E-12	6.28E-15	8.39E-02
Prostate cancer	Human Diseases	Cancer: specific types	398	382	392	6.07E-07	1.51E-02	1.25E-02
Small cell lung cancer	Human Diseases	Cancer: specific types	564.5	533	544	9.81E-12	3.86E-05	3.26E-03
Non-small cell lung cancer	Human Diseases	Cancer: specific types	543	506	525	6.09E-13	1.28E-02	1.06E-09
Neurodegenerative disease	Human Diseases	Neurodegenerative disease	1236.5	1195	1200	1.80E-09	1.91E-06	1.75E-01
Parkinson disease	Human Diseases	Neurodegenerative disease	133	130	127	2.20E-02	1.62E-03	3.24E-01
Huntington disease	Human Diseases	Neurodegenerative disease	617.5	592	604	1.65E-10	3.49E-03	2.76E-04
Substance dependence	Human Diseases	Substance dependence	1294	1232	1276	1.04E-17	3.81E-03	1.97E-11
Morphine addiction	Human Diseases	Substance dependence	715.5	682	698	1.23E-08	8.94E-05	2.90E-02
Cardiovascular disease	Human Diseases	Cardiovascular disease	1476	1461	1408	8.66E-03	2.04E-16	1.27E-12
Hypertrophic cardiomyopathy (HCM)	Human Diseases	Cardiovascular disease	639	623	606	3.14E-02	6.21E-10	4.39E-07
Arrhythmogenic right ventricular cardiomyopathy (ARVC)	Human Diseases	Cardiovascular disease	874	857	822	5.38E-03	2.42E-14	5.96E-09
Endocrine and metabolic disease	Human Diseases	Endocrine and metabolic disease	1626.5	1581	1584	4.79E-12	2.02E-07	2.42E-01
Type II diabetes mellitus	Human Diseases	Endocrine and metabolic disease	224	217	217	5.89E-03	2.11E-04	3.11E-01
Cushing syndrome	Human Diseases	Endocrine and metabolic disease	734	707	718	2.07E-06	1.63E-03	5.24E-02
Infectious disease: viral	Human Diseases	Infectious disease: viral	2779.5	2702	2699	4.79E-12	1.48E-12	6.60E-01
Human cytomegalovirus infection	Human Diseases	Infectious disease: viral	771	754	748	4.20E-05	1.66E-06	5.25E-01
Human papillomavirus infection	Human Diseases	Infectious disease: viral	1338.5	1304	1305	5.58E-08	3.08E-07	9.48E-01
Human T-cell leukemia virus 1 infection	Human Diseases	Infectious disease: viral	568.5	557	559	2.27E-03	4.51E-03	8.37E-01
Kaposi sarcoma-associated herpesvirus infection	Human Diseases	Infectious disease: viral	400.5	393	392	4.39E-04	8.90E-05	8.73E-01
Herpes simplex virus 1 infection	Human Diseases	Infectious disease: viral	322	312	309	5.05E-04	2.50E-05	3.75E-01

Table S5 | Genes of higher genetic burden in XJU individuals.

Chr	Start	End	# Deleterious SNVs (median)			BH-corrected *P*-value (Wilcoxon rank sum test)			Symbol	Type
			XJU	EAS	EUR	XJU vs. EAS	XJU vs. EUR	EAS vs. EUR		
**Genetic burden estimated as counts of LoF and missense variants under the dominant model**										
14	105917899	105967898	2	2	2	2.62E-02	1.31E-02	9.20E-01	CRIP2	protein_coding
14	105928969	105978968	2	2	2	2.62E-02	1.31E-02	9.20E-01	CRIP1	protein_coding
14	106988208	107038207	3	1	1	1.83E-09	1.93E-11	1.49E-01	IGHV3-49	protein_coding
14	106995475	107045474	3	1	1	1.83E-09	1.93E-11	1.49E-01	IGHVII-49-1	pseudogene
14	106997324	107047323	3	1	1	1.83E-09	1.93E-11	1.49E-01	IGHV3-50	pseudogene
14	107009975	107059974	3	1	1	1.83E-09	1.93E-11	1.49E-01	IGHV5-51	protein_coding
14	107030809	107080808	3	2	2	1.68E-06	5.53E-08	6.08E-01	IGHVII-53-1	pseudogene
14	107032577	107082576	3	2	2	1.68E-06	5.53E-08	6.08E-01	IGHV3-54	pseudogene
22	22682535	22732534	2	1	2	1.40E-07	2.20E-03	9.03E-02	IGLV5-48	protein_coding
22	22687348	22737347	2	1	2	1.40E-07	2.20E-03	9.03E-02	IGLV1-47	protein_coding
22	22696358	22746357	2	1	2	1.40E-07	2.20E-03	9.03E-02	LL22NC03-22A12.9	pseudogene
22	22699218	22749217	2	1	2	8.89E-07	8.18E-03	6.96E-02	IGLV7-46	protein_coding
22	22700343	22750342	2	1	2	8.89E-07	1.10E-02	6.38E-02	LL22NC03-22A12.12	pseudogene
22	22705615	22755614	2	1	2	8.89E-07	1.10E-02	6.38E-02	IGLV5-45	protein_coding
**Genetic burden estimated as counts of variants with CADD score >15 under the dominant model**										
6	31659357	31709356	2	1	1	3.15E-02	4.30E-02	1.00E+00	LY6G6D	protein_coding
6	31663024	31713023	2	1	1	3.69E-02	3.28E-02	1.00E+00	LY6G6C	protein_coding
6	31665431	31715430	2	1	1	3.69E-02	3.28E-02	1.00E+00	C6orf25	protein_coding
**Genetic burden estimated as counts of variants with GERP score >2 under the dominant model**										
1	14425779	14475778	6	6	6	6.64E-03	1.48E-02	3.41E-01	RNU6-1265P	noncoding_RNA
1	36157275	36207274	2	1	1	1.69E-03	3.24E-02	6.42E-01	C1orf216	protein_coding
1	41972036	42501596	38	35	36	2.14E-04	4.47E-02	2.01E-01	HIVEP3	protein_coding
1	180915178	180965177	3	3	3	4.67E-02	1.09E-02	9.38E-01	RP11-46A10.8	pseudogene
1	198776622	198906558	7	6	6	1.47E-02	1.21E-02	6.64E-01	MIR181A1HG	noncoding_RNA
1	199996730	200146552	15	13	13	1.01E-02	2.06E-03	4.22E-01	NR5A2	protein_coding
2	28194275	28244274	3	1	3	1.22E-02	4.33E-02	4.37E-02	MIR4263	noncoding_RNA
2	37947833	37997832	6	4	5	4.17E-09	5.73E-04	4.26E-03	AC006369.3	noncoding_RNA
2	45878484	46415129	45.5	42	41	2.64E-02	2.52E-03	4.26E-01	PRKCE	protein_coding
2	46280561	46330560	6	5	5	1.06E-02	2.03E-02	9.59E-01	AC017006.2	noncoding_RNA
2	46520806	46613836	7	6	6	8.12E-03	1.39E-02	7.40E-01	EPAS1	protein_coding
2	66660584	66801001	15	14	13	1.69E-02	4.54E-02	6.84E-01	MEIS1	protein_coding
2	109510927	109605828	7	6	7	5.82E-03	2.16E-03	2.63E-01	EDAR	protein_coding
2	138244724	138294723	5	3	3	6.40E-03	1.30E-02	9.35E-01	RNA5SP105	noncoding_RNA
2	173940163	174132738	15.5	13	13	6.18E-03	3.15E-03	8.69E-01	MLTK	protein_coding
2	179966419	180129517	2	1	2	1.45E-03	8.06E-03	6.03E-01	SESTD1	protein_coding
3	57076965	57126964	4	4	4	1.52E-02	2.11E-02	9.15E-01	SPATA12	protein_coding
3	59735036	61237133	191.5	174	180	3.64E-09	6.62E-03	2.18E-03	FHIT	protein_coding
3	117368590	117418589	2	1	1	5.42E-03	2.21E-02	8.43E-01	RP11-768G7.1	pseudogene
4	23756664	23905712	17	15	14	1.74E-02	3.72E-02	1.00E+00	PPARGC1A	protein_coding
4	101316498	101801283	27	25	25	1.90E-03	2.05E-02	1.00E+00	EMCN	protein_coding
4	105151537	105332192	4	3	3	3.22E-02	1.07E-02	9.83E-01	RP11-729M20.1	noncoding_RNA
4	122578689	122628688	4.5	4	4	4.72E-02	2.32E-02	7.65E-01	ANXA5	protein_coding
5	40998119	41071444	9	8	7	1.29E-02	4.85E-02	9.83E-01	MROH2B	protein_coding
5	131676237	131726236	3	2	2	6.71E-05	1.26E-02	4.86E-01	MIR3936	noncoding_RNA
5	131693375	131743374	5	4	3	9.50E-04	5.51E-03	6.42E-01	SLC22A5	protein_coding
6	31771772	31821771	1	1	1	1.41E-02	3.22E-02	9.94E-01	HSPA1B	protein_coding
6	32008931	32083111	5	4	4	1.41E-03	8.77E-03	5.86E-01	TNXB	protein_coding
6	32021345	32071344	4	3	3	6.27E-03	3.00E-02	7.89E-01	RNA5SP206	noncoding_RNA
6	72911472	72961471	6	6	5	4.31E-02	1.54E-02	3.36E-01	AL445256.1	noncoding_RNA
6	112557294	112671296	6	5	5	4.11E-02	3.46E-02	9.44E-01	RP11-506B6.6	noncoding_RNA
6	114077593	114127592	3	2	2	1.10E-02	4.81E-02	2.17E-01	U3	noncoding_RNA
6	161768452	163148803	82	78	77	3.68E-02	1.08E-02	5.19E-01	PARK2	protein_coding
8	120177273	120257913	5	4	4	1.09E-03	2.25E-03	9.64E-01	MAL2	protein_coding
8	145664842	145714841	4	4	4	1.24E-02	5.53E-03	8.85E-01	CTD-2517M22.16	noncoding_RNA

Chr	Start	End	# Deleterious SNVs (median)			BH-corrected *P*-value (Wilcoxon rank sum test)			Symbol	Type
			XJU	EAS	EUR	XJU vs. EAS	XJU vs. EUR	EAS vs. EUR		
8	145670506	145720505	3	3	3	1.71E-02	1.21E-02	8.05E-01	KIFC2	protein_coding
8	145675257	145725256	3	3	3	1.78E-02	1.72E-02	7.54E-01	FOXH1	protein_coding
9	33927808	33977807	3	2	3	3.22E-02	4.85E-02	5.33E-01	SNORD121A	noncoding_RNA
9	33943293	33993292	3	1	2	9.39E-03	3.65E-02	9.35E-02	OSTCP8	pseudogene
10	20345224	20395223	5	4	4	9.01E-03	1.82E-02	5.75E-01	RP11-575A19.2	noncoding_RNA
10	94942999	94992998	4	4	4	3.57E-02	1.17E-02	4.20E-01	XRCC6P1	pseudogene
11	8034129	8084128	3	3	3	1.97E-03	4.69E-02	3.49E-01	RP11-236J17.6	noncoding_RNA
11	34172535	34379555	21	19	19	2.73E-02	4.79E-02	8.48E-01	ABTB2	protein_coding
12	861759	1020618	10	9	9	7.66E-05	3.76E-02	9.38E-02	WNK1	protein_coding
12	2874779	2924778	2	1	2	3.50E-02	3.32E-02	8.47E-01	CBX3P4	pseudogene
12	53392968	53442967	6	6	5	3.80E-02	1.78E-03	1.14E-01	EIF4B	protein_coding
12	53403301	53453300	6	5	5	4.32E-04	3.79E-02	3.22E-02	RP11-983P16.4	noncoding_RNA
14	29211960	29261959	7	6	5	3.62E-02	1.28E-02	6.47E-01	FOXG1	protein_coding
14	51955818	52197445	25	22	23	2.27E-02	1.17E-02	9.79E-01	FRMD6	protein_coding
14	72399156	73030654	47.5	44	43	1.26E-03	1.19E-03	7.59E-01	RGS6	protein_coding
17	18012020	18083116	4	3	3	4.13E-03	1.15E-03	5.06E-01	MYO15A	protein_coding
18	21269407	21535030	10	9	9	4.67E-02	1.73E-02	9.25E-01	LAMA3	protein_coding
22	23521891	23660224	2	1	1	2.63E-02	1.58E-02	7.29E-01	BCR	protein_coding
22	30614638	30664637	2	1	1	3.03E-02	3.74E-02	1.00E+00	LIF	protein_coding
22	30617486	30667485	2	1	1	3.03E-02	3.74E-02	1.00E+00	RP1-102K2.8	noncoding_RNA
22	50613248	50663247	2	1	1.5	1.32E-02	7.53E-03	1.00E+00	RP3-402G11.26	noncoding_RNA

Table S6 | Pathways enriched for genes of high AFd_e in analysis of mGSEA.

Pathway	Super pathway	# Genes	# Leading-edge genes	Enrichment Score	BH-corrected P-value
**2-references-based AFd_e**					
Global and overview maps	Global and overview maps	1309	526	0.068	9.79E-04
Metabolic pathways	Global and overview maps	1304	521	0.066	1.50E-03
Fatty acid metabolism	Global and overview maps	51	31	0.224	2.78E-02
Propanoate metabolism	Carbohydrate metabolism	32	25	0.327	7.19E-03
Pentose and glucuronate interconversions	Carbohydrate metabolism	34	11	0.286	1.98E-02
Ascorbate and aldarate metabolism	Carbohydrate metabolism	26	9	0.315	2.00E-02
Pyruvate metabolism	Carbohydrate metabolism	38	31	0.249	3.35E-02
Fatty acid degradation	Lipid metabolism	43	29	0.291	4.92E-03
Lipid metabolism	Lipid metabolism	365	67	0.088	1.91E-02
Steroid hormone biosynthesis	Lipid metabolism	58	19	0.207	3.28E-02
Nucleotide metabolism	Nucleotide metabolism	143	66	0.158	7.42E-03
Purine metabolism	Nucleotide metabolism	120	56	0.163	1.14E-02
Amino acid metabolism	Amino acid metabolism	264	141	0.140	9.79E-04
Valine, leucine and isoleucine degradation	Amino acid metabolism	47	29	0.293	4.11E-03
beta-Alanine metabolism	Metabolism of other amino acids	30	18	0.276	3.85E-02
Glycosphingolipid biosynthesis - ganglio series	Glycan biosynthesis and metabolism	15	11	0.468	7.14E-03
Mucin type O-glycan biosynthesis	Glycan biosynthesis and metabolism	29	17	0.288	3.32E-02
Glycan biosynthesis and metabolism	Glycan biosynthesis and metabolism	235	127	0.097	4.15E-02
Thiamine metabolism	Metabolism of cofactors and vitamins	15	13	0.495	3.28E-03
Metabolism of cofactors and vitamins	Metabolism of cofactors and vitamins	207	95	0.130	8.49E-03
Retinol metabolism	Metabolism of cofactors and vitamins	62	18	0.194	3.72E-02
Metabolism	Metabolism	1494	699	0.070	9.79E-04
RNA transport	Translation	137	103	0.129	4.15E-02
ABC transporters	Membrane transport	43	38	0.231	3.76E-02
Membrane transport	Membrane transport	43	38	0.231	3.99E-02
MAPK signaling pathway	Signal transduction	281	229	0.158	9.79E-04
Calcium signaling pathway	Signal transduction	183	147	0.190	9.79E-04
Rap1 signaling pathway	Signal transduction	200	168	0.178	9.79E-04
Ras signaling pathway	Signal transduction	220	185	0.184	9.79E-04
Signal transduction	Signal transduction	1670	1096	0.123	9.79E-04
cAMP signaling pathway	Signal transduction	207	161	0.164	1.50E-03
PI3K-Akt signaling pathway	Signal transduction	342	251	0.116	1.97E-03
Wnt signaling pathway	Signal transduction	153	108	0.165	1.97E-03
cGMP-PKG signaling pathway	Signal transduction	157	122	0.173	1.97E-03
Sphingolipid signaling pathway	Signal transduction	116	84	0.188	2.87E-03
ErbB signaling pathway	Signal transduction	80	40	0.218	2.87E-03
Apelin signaling pathway	Signal transduction	132	66	0.171	4.55E-03
TNF signaling pathway	Signal transduction	107	50	0.186	5.40E-03
Phospholipase D signaling pathway	Signal transduction	139	113	0.160	5.74E-03
mTOR signaling pathway	Signal transduction	146	76	0.149	1.16E-02
AMPK signaling pathway	Signal transduction	116	43	0.150	2.43E-02
Phosphatidylinositol signaling system	Signal transduction	92	79	0.165	3.14E-02
Hippo signaling pathway	Signal transduction	150	108	0.124	3.76E-02
HIF-1 signaling pathway	Signal transduction	94	43	0.154	4.44E-02
Signaling molecules and interaction	Signaling molecules and interaction	788	449	0.101	9.79E-04
ECM-receptor interaction	Signaling molecules and interaction	84	74	0.281	9.79E-04
Neuroactive ligand-receptor interaction	Signaling molecules and interaction	318	184	0.099	9.77E-03
EnvironmentalInformationProcessing	EnvironmentalInformationProcessing	2131	1513	0.119	9.79E-04
Transport and catabolism	Transport and catabolism	673	423	0.073	6.87E-03
Endocytosis	Transport and catabolism	226	122	0.102	3.29E-02
Autophagy - animal	Transport and catabolism	117	58	0.135	4.97E-02
Cell growth and death	Cell growth and death	552	221	0.077	8.14E-03
Cellular senescence	Cell growth and death	152	72	0.144	1.08E-02
Oocyte meiosis	Cell growth and death	110	88	0.152	2.85E-02
Cellular community - eukaryotes	Cellular community - eukaryotes	533	366	0.151	9.79E-04
Focal adhesion	Cellular community - eukaryotes	189	139	0.199	9.79E-04
Adherens junction	Cellular community - eukaryotes	66	53	0.262	1.50E-03
Tight junction	Cellular community - eukaryotes	162	138	0.134	1.52E-02

Pathway	Super pathway	# Genes	# Leading-edge genes	Enrichment Score	BH-corrected P-value
Regulation of actin cytoskeleton	Cell motility	200	141	0.164	9.79E-04
Cell motility	Cell motility	200	141	0.164	9.79E-04
CellularProcesses	CellularProcesses	1577	1030	0.100	9.79E-04
Immune system	Immune system	963	626	0.085	9.79E-04
Platelet activation	Immune system	120	93	0.189	2.46E-03
C-type lectin receptor signaling pathway	Immune system	102	76	0.204	3.28E-03
Chemokine signaling pathway	Immune system	178	121	0.134	1.01E-02
Th17 cell differentiation	Immune system	101	73	0.166	1.80E-02
T cell receptor signaling pathway	Immune system	95	48	0.165	2.11E-02
Leukocyte transendothelial migration	Immune system	105	70	0.155	2.44E-02
Th1 and Th2 cell differentiation	Immune system	87	64	0.169	2.80E-02
Fc epsilon RI signaling pathway	Immune system	64	27	0.190	3.71E-02
IL-17 signaling pathway	Immune system	87	62	0.162	3.85E-02
Fc gamma R-mediated phagocytosis	Immune system	83	61	0.168	3.99E-02
Environmental adaptation	Environmental adaptation	291	236	0.132	9.79E-04
Circadian entrainment	Environmental adaptation	94	79	0.254	9.79E-04
Cortisol synthesis and secretion	Endocrine system	63	38	0.279	9.79E-04
Endocrine system	Endocrine system	876	383	0.115	9.79E-04
Oxytocin signaling pathway	Endocrine system	149	111	0.173	1.50E-03
Aldosterone synthesis and secretion	Endocrine system	95	81	0.248	1.50E-03
Parathyroid hormone synthesis, secretion and action	Endocrine system	104	83	0.198	2.46E-03
Insulin secretion	Endocrine system	83	76	0.213	2.87E-03
Estrogen signaling pathway	Endocrine system	136	104	0.173	3.70E-03
Relaxin signaling pathway	Endocrine system	127	62	0.164	6.87E-03
Melanogenesis	Endocrine system	101	79	0.182	7.90E-03
Thyroid hormone synthesis	Endocrine system	72	64	0.204	1.16E-02
Progesterone-mediated oocyte maturation	Endocrine system	85	44	0.188	1.53E-02
Glucagon signaling pathway	Endocrine system	95	54	0.174	1.54E-02
GnRH signaling pathway	Endocrine system	90	36	0.174	1.91E-02
PPAR signaling pathway	Endocrine system	73	39	0.194	1.91E-02
Renin secretion	Endocrine system	66	31	0.205	2.36E-02
Circulatory system	Circulatory system	258	220	0.160	9.79E-04
Adrenergic signaling in cardiomyocytes	Circulatory system	142	124	0.184	1.50E-03
Vascular smooth muscle contraction	Circulatory system	126	54	0.180	2.46E-03
Digestive system	Digestive system	398	229	0.160	9.79E-04
Gastric acid secretion	Digestive system	74	66	0.244	1.50E-03
Salivary secretion	Digestive system	83	61	0.224	3.70E-03
Pancreatic secretion	Digestive system	92	65	0.195	8.14E-03
Protein digestion and absorption	Digestive system	80	42	0.201	9.09E-03
Vitamin digestion and absorption	Digestive system	23	17	0.350	1.41E-02
Bile secretion	Digestive system	71	64	0.204	1.72E-02
Endocrine and other factor-regulated calcium reabsorption	Excretory system	47	43	0.230	2.85E-02
Glutamatergic synapse	Nervous system	111	90	0.224	9.79E-04
Cholinergic synapse	Nervous system	111	64	0.247	9.79E-04
Nervous system	Nervous system	502	369	0.145	9.79E-04
Long-term potentiation	Nervous system	64	50	0.270	1.97E-03
Dopaminergic synapse	Nervous system	127	89	0.190	1.97E-03
GABAergic synapse	Nervous system	84	51	0.246	1.97E-03
Retrograde endocannabinoid signaling	Nervous system	134	114	0.171	4.11E-03
Synaptic vesicle cycle	Nervous system	78	61	0.210	6.02E-03
Neurotrophin signaling pathway	Nervous system	113	75	0.142	4.15E-02
Inflammatory mediator regulation of TRP channels	Sensory system	98	76	0.239	9.79E-04
Development and regeneration	Development and regeneration	277	191	0.179	9.79E-04
Axon guidance	Development and regeneration	173	128	0.229	9.79E-04
Longevity regulating pathway - multiple species	Aging	60	32	0.230	1.04E-02
OrganismalSystems	OrganismalSystems	2910	1275	0.070	9.79E-04
Pathways in cancer	Cancer: overview	510	385	0.113	9.79E-04
Cancer: overview	Cancer: overview	1170	643	0.098	9.79E-04
Proteoglycans in cancer	Cancer: overview	195	132	0.136	5.74E-03
PD-L1 expression and PD-1 checkpoint pathway in cancer	Cancer: overview	87	36	0.182	1.79E-02
Transcriptional misregulation in cancer	Cancer: overview	170	96	0.127	2.02E-02

Pathway	Super pathway	# Genes	# Leading-edge genes	Enrichment Score	BH-corrected P-value
MicroRNAs in cancer	Cancer: overview	268	89	0.101	2.12E-02
Choline metabolism in cancer	Cancer: overview	92	75	0.162	3.20E-02
Infectious disease: parasitic	Infectious disease: parasitic	255	181	0.129	2.87E-03
Amoebiasis	Infectious disease: parasitic	90	67	0.178	1.91E-02
Chagas disease (American trypanosomiasis)	Infectious disease: parasitic	98	65	0.147	4.43E-02
Toxoplasmosis	Infectious disease: parasitic	103	74	0.147	4.64E-02
Endocrine resistance	Drug resistance: antineoplastic	94	52	0.229	9.79E-04
Drug resistance: antineoplastic	Drug resistance: antineoplastic	211	100	0.155	1.50E-03
EGFR tyrosine kinase inhibitor resistance	Drug resistance: antineoplastic	78	27	0.177	3.31E-02
Cancer: specific types	Cancer: specific types	467	361	0.126	9.79E-04
Breast cancer	Cancer: specific types	145	108	0.163	3.28E-03
Gastric cancer	Cancer: specific types	147	100	0.159	4.11E-03
Glioma	Cancer: specific types	74	53	0.195	1.73E-02
Non-small cell lung cancer	Cancer: specific types	64	26	0.186	4.47E-02
Melanoma	Cancer: specific types	70	48	0.174	4.95E-02
Systemic lupus erythematosus	Immune disease	113	62	0.143	3.82E-02
Immune disease	Immune disease	288	134	0.088	4.29E-02
Neurodegenerative disease	Neurodegenerative disease	316	250	0.094	1.95E-02
Huntington disease	Neurodegenerative disease	167	137	0.118	4.18E-02
Substance dependence	Substance dependence	258	151	0.186	9.79E-04
Morphine addiction	Substance dependence	86	57	0.263	1.50E-03
Nicotine addiction	Substance dependence	36	27	0.373	1.50E-03
Amphetamine addiction	Substance dependence	65	52	0.200	2.44E-02
Arrhythmogenic right ventricular cardiomyopathy (ARVC)	Cardiovascular disease	71	56	0.262	9.79E-04
Cardiovascular disease	Cardiovascular disease	278	188	0.120	2.46E-03
Dilated cardiomyopathy (DCM)	Cardiovascular disease	89	63	0.181	1.72E-02
Hypertrophic cardiomyopathy (HCM)	Cardiovascular disease	84	59	0.176	2.44E-02
Cushing syndrome	Endocrine and metabolic disease	154	98	0.183	9.79E-04
Endocrine and metabolic disease	Endocrine and metabolic disease	472	158	0.097	1.50E-03
AGE-RAGE signaling pathway in diabetic complications	Endocrine and metabolic disease	94	38	0.172	1.99E-02
Type II diabetes mellitus	Endocrine and metabolic disease	45	21	0.235	3.14E-02
Infectious disease: bacterial	Infectious disease: bacterial	451	142	0.084	1.27E-02
Epithelial cell signaling in Helicobacter pylori infection	Infectious disease: bacterial	67	47	0.190	3.21E-02
Bacterial invasion of epithelial cells	Infectious disease: bacterial	72	50	0.183	3.35E-02
Infectious disease: viral	Infectious disease: viral	1158	745	0.068	9.79E-04
Human papillomavirus infection	Infectious disease: viral	322	204	0.113	1.50E-03
Human cytomegalovirus infection	Infectious disease: viral	216	75	0.127	6.02E-03
Human T-cell leukemia virus 1 infection	Infectious disease: viral	210	74	0.115	1.73E-02
Human immunodeficiency virus 1 infection	Infectious disease: viral	198	66	0.113	2.85E-02
Kaposi sarcoma-associated herpesvirus infection	Infectious disease: viral	182	60	0.109	4.56E-02
HumanDiseases	HumanDiseases	2796	1812	0.075	9.79E-04
ADME	ADME	283	157	0.134	1.97E-03
**4-references-based AFd$_e$**					
Global and overview maps	Global and overview maps	1305	685	0.058	1.89E-03
Metabolic pathways	Global and overview maps	1300	681	0.057	2.56E-03
Ascorbate and aldarate metabolism	Carbohydrate metabolism	24	9	0.344	8.88E-03
Pentose and glucuronate interconversions	Carbohydrate metabolism	32	11	0.258	4.93E-02
Nucleotide metabolism	Nucleotide metabolism	143	79	0.152	7.45E-03
Purine metabolism	Nucleotide metabolism	120	63	0.149	1.75E-02
Lysine degradation	Amino acid metabolism	52	46	0.223	1.99E-02
Glycan biosynthesis and metabolism	Glycan biosynthesis and metabolism	235	123	0.128	2.56E-03
Glycosaminoglycan biosynthesis - heparan sulfate / heparin	Glycan biosynthesis and metabolism	23	15	0.392	4.44E-03
Glycosphingolipid biosynthesis - ganglio series	Glycan biosynthesis and metabolism	15	11	0.372	4.81E-02
Retinol metabolism	Metabolism of cofactors and vitamins	60	36	0.215	1.58E-02
Porphyrin and chlorophyll metabolism	Metabolism of cofactors and vitamins	37	11	0.242	4.79E-02
Metabolism	Metabolism	1490	792	0.055	2.21E-03
Ubiquitin mediated proteolysis	Folding, sorting and degradation	124	67	0.150	1.69E-02
Fanconi anemia pathway	Replication and repair	45	31	0.241	1.92E-02
ErbB signaling pathway	Signal transduction	80	69	0.267	7.33E-04
Signal transduction	Signal transduction	1671	982	0.103	7.33E-04
cAMP signaling pathway	Signal transduction	207	111	0.166	7.33E-04

Pathway	Super pathway	# Genes	# Leading-edge genes	Enrichment Score	BH-corrected P-value
Rap1 signaling pathway	Signal transduction	200	92	0.195	7.33E-04
Calcium signaling pathway	Signal transduction	183	58	0.155	1.54E-03
Ras signaling pathway	Signal transduction	220	149	0.142	1.54E-03
MAPK signaling pathway	Signal transduction	281	207	0.122	1.54E-03
PI3K-Akt signaling pathway	Signal transduction	342	268	0.108	1.89E-03
cGMP-PKG signaling pathway	Signal transduction	158	55	0.168	1.89E-03
Sphingolipid signaling pathway	Signal transduction	117	88	0.171	4.44E-03
TNF signaling pathway	Signal transduction	107	76	0.190	4.44E-03
Phospholipase D signaling pathway	Signal transduction	140	93	0.155	5.06E-03
TGF-beta signaling pathway	Signal transduction	93	44	0.197	5.23E-03
mTOR signaling pathway	Signal transduction	146	123	0.136	1.74E-02
AMPK signaling pathway	Signal transduction	116	87	0.154	1.82E-02
Wnt signaling pathway	Signal transduction	153	84	0.133	1.92E-02
Apelin signaling pathway	Signal transduction	134	45	0.126	4.72E-02
ECM-receptor interaction	Signaling molecules and interaction	84	72	0.252	7.33E-04
Cell adhesion molecules (CAMs)	Signaling molecules and interaction	130	57	0.195	7.33E-04
Signaling molecules and interaction	Signaling molecules and interaction	788	275	0.107	7.33E-04
Neuroactive ligand-receptor interaction	Signaling molecules and interaction	318	90	0.097	1.48E-02
EnvironmentalInformationProcessing	EnvironmentalInformationProcessing	2132	1247	0.101	7.33E-04
Transport and catabolism	Transport and catabolism	668	449	0.092	7.33E-04
Endocytosis	Transport and catabolism	226	161	0.132	1.54E-03
Cell growth and death	Cell growth and death	548	319	0.095	1.19E-03
Cellular senescence	Cell growth and death	149	113	0.163	2.85E-03
Oocyte meiosis	Cell growth and death	109	40	0.157	2.06E-02
Gap junction	Cellular community - eukaryotes	83	35	0.223	7.33E-04
Focal adhesion	Cellular community - eukaryotes	189	149	0.173	7.33E-04
Adherens junction	Cellular community - eukaryotes	66	38	0.305	7.33E-04
Cellular community - eukaryotes	Cellular community - eukaryotes	531	215	0.146	7.33E-04
Tight junction	Cellular community - eukaryotes	161	60	0.129	1.95E-02
Regulation of actin cytoskeleton	Cell motility	201	90	0.139	2.21E-03
Cell motility	Cell motility	201	90	0.139	2.21E-03
CellularProcesses	CellularProcesses	1569	1057	0.096	7.33E-04
Immune system	Immune system	957	346	0.092	7.33E-04
Platelet activation	Immune system	121	54	0.242	7.33E-04
T cell receptor signaling pathway	Immune system	95	77	0.215	1.89E-03
Antigen processing and presentation	Immune system	62	34	0.267	2.21E-03
Hematopoietic cell lineage	Immune system	88	61	0.194	5.49E-03
Fc gamma R-mediated phagocytosis	Immune system	81	50	0.201	5.73E-03
Chemokine signaling pathway	Immune system	178	69	0.133	7.64E-03
Th1 and Th2 cell differentiation	Immune system	87	68	0.191	8.40E-03
Th17 cell differentiation	Immune system	101	83	0.174	8.60E-03
Intestinal immune network for IgA production	Immune system	43	22	0.262	1.16E-02
Leukocyte transendothelial migration	Immune system	105	56	0.162	1.45E-02
C-type lectin receptor signaling pathway	Immune system	103	59	0.151	3.39E-02
Circadian entrainment	Environmental adaptation	94	43	0.248	7.33E-04
Environmental adaptation	Environmental adaptation	291	193	0.128	1.54E-03
Thermogenesis	Environmental adaptation	195	144	0.105	4.89E-02
Oxytocin signaling pathway	Endocrine system	149	57	0.209	7.33E-04
Cortisol synthesis and secretion	Endocrine system	63	29	0.304	7.33E-04
Aldosterone synthesis and secretion	Endocrine system	95	40	0.265	7.33E-04
Endocrine system	Endocrine system	875	287	0.097	7.33E-04
Insulin secretion	Endocrine system	83	33	0.258	7.33E-04
Parathyroid hormone synthesis, secretion and action	Endocrine system	105	49	0.218	1.19E-03
Estrogen signaling pathway	Endocrine system	136	86	0.211	1.19E-03
GnRH signaling pathway	Endocrine system	90	35	0.221	1.54E-03
Thyroid hormone synthesis	Endocrine system	72	29	0.229	2.85E-03
Renin secretion	Endocrine system	66	26	0.238	2.85E-03
Prolactin signaling pathway	Endocrine system	70	60	0.232	3.14E-03
Relaxin signaling pathway	Endocrine system	127	52	0.166	5.23E-03
Progesterone-mediated oocyte maturation	Endocrine system	84	56	0.193	7.35E-03
Melanogenesis	Endocrine system	101	35	0.173	1.02E-02

Pathway	Super pathway	# Genes	# Leading-edge genes	Enrichment Score	BH-corrected P-value
Glucagon signaling pathway	Endocrine system	95	30	0.165	1.75E-02
Circulatory system	Circulatory system	259	103	0.171	7.33E-04
Adrenergic signaling in cardiomyocytes	Circulatory system	142	78	0.225	7.33E-04
Vascular smooth muscle contraction	Circulatory system	127	55	0.212	7.33E-04
Digestive system	Digestive system	398	183	0.109	1.54E-03
Salivary secretion	Digestive system	83	31	0.217	1.89E-03
Gastric acid secretion	Digestive system	74	29	0.236	2.21E-03
Protein digestion and absorption	Digestive system	80	65	0.197	7.45E-03
Pancreatic secretion	Digestive system	92	32	0.186	8.40E-03
Dopaminergic synapse	Nervous system	127	52	0.200	7.33E-04
Long-term potentiation	Nervous system	64	28	0.287	7.33E-04
Nervous system	Nervous system	503	363	0.153	7.33E-04
Glutamatergic synapse	Nervous system	111	49	0.232	7.33E-04
Cholinergic synapse	Nervous system	111	46	0.270	7.33E-04
Neurotrophin signaling pathway	Nervous system	113	91	0.210	1.54E-03
GABAergic synapse	Nervous system	84	31	0.213	4.44E-03
Retrograde endocannabinoid signaling	Nervous system	134	57	0.165	5.23E-03
Long-term depression	Nervous system	56	23	0.212	2.71E-02
Inflammatory mediator regulation of TRP channels	Sensory system	98	47	0.225	1.19E-03
Development and regeneration	Development and regeneration	274	167	0.157	7.33E-04
Axon guidance	Development and regeneration	173	101	0.203	7.33E-04
Aging	Aging	98	33	0.180	5.23E-03
Longevity regulating pathway - multiple species	Aging	59	23	0.233	7.64E-03
Longevity regulating pathway	Aging	87	29	0.177	1.69E-02
OrganismalSystems	OrganismalSystems	2897	967	0.072	7.33E-04
Viral carcinogenesis	Cancer: overview	188	128	0.172	7.33E-04
Cancer: overview	Cancer: overview	1167	744	0.104	7.33E-04
Proteoglycans in cancer	Cancer: overview	195	129	0.163	1.19E-03
Transcriptional misregulation in cancer	Cancer: overview	170	121	0.177	1.19E-03
Pathways in cancer	Cancer: overview	508	301	0.094	1.89E-03
MicroRNAs in cancer	Cancer: overview	267	183	0.105	1.43E-02
Choline metabolism in cancer	Cancer: overview	92	69	0.154	4.79E-02
Leishmaniasis	Infectious disease: parasitic	61	33	0.281	7.33E-04
Infectious disease: parasitic	Infectious disease: parasitic	253	120	0.145	1.19E-03
Toxoplasmosis	Infectious disease: parasitic	103	45	0.171	9.58E-03
Amoebiasis	Infectious disease: parasitic	90	31	0.176	1.35E-02
Endocrine resistance	Drug resistance: antineoplastic	94	74	0.207	1.19E-03
Drug resistance: antineoplastic	Drug resistance: antineoplastic	209	139	0.167	1.19E-03
EGFR tyrosine kinase inhibitor resistance	Drug resistance: antineoplastic	78	68	0.210	6.39E-03
Glioma	Cancer: specific types	74	62	0.242	7.33E-04
Cancer: specific types	Cancer: specific types	464	294	0.125	7.33E-04
Chronic myeloid leukemia	Cancer: specific types	73	61	0.240	1.19E-03
Prostate cancer	Cancer: specific types	93	68	0.222	1.54E-03
Non-small cell lung cancer	Cancer: specific types	64	53	0.232	5.06E-03
Pancreatic cancer	Cancer: specific types	72	52	0.213	8.18E-03
Small cell lung cancer	Cancer: specific types	89	62	0.188	9.58E-03
Endometrial cancer	Cancer: specific types	55	45	0.222	1.75E-02
Hepatocellular carcinoma	Cancer: specific types	163	102	0.127	2.06E-02
Gastric cancer	Cancer: specific types	147	114	0.123	3.83E-02
Systemic lupus erythematosus	Immune disease	111	75	0.228	7.33E-04
Asthma	Immune disease	26	18	0.438	1.19E-03
Immune disease	Immune disease	283	183	0.116	2.85E-03
Allograft rejection	Immune disease	33	19	0.343	3.84E-03
Inflammatory bowel disease (IBD)	Immune disease	60	44	0.208	1.99E-02
Neurodegenerative disease	Neurodegenerative disease	315	232	0.156	7.33E-04
Huntington disease	Neurodegenerative disease	166	117	0.154	3.85E-03
Amyotrophic lateral sclerosis (ALS)	Neurodegenerative disease	50	41	0.239	1.23E-02
Prion diseases	Neurodegenerative disease	34	28	0.268	2.86E-02
Alzheimer disease	Neurodegenerative disease	147	55	0.125	3.37E-02
Alcoholism	Substance dependence	161	110	0.180	7.33E-04
Substance dependence	Substance dependence	257	165	0.200	7.33E-04

Pathway	Super pathway	# Genes	# Leading-edge genes	Enrichment Score	BH-corrected P-value
Morphine addiction	Substance dependence	86	33	0.227	7.33E-04
Amphetamine addiction	Substance dependence	64	32	0.251	3.54E-03
Nicotine addiction	Substance dependence	36	21	0.323	4.44E-03
Cocaine addiction	Substance dependence	47	29	0.241	1.69E-02
Cardiovascular disease	Cardiovascular disease	276	114	0.148	7.33E-04
Arrhythmogenic right ventricular cardiomyopathy (ARVC)	Cardiovascular disease	71	41	0.274	7.33E-04
Dilated cardiomyopathy (DCM)	Cardiovascular disease	89	41	0.234	7.33E-04
Hypertrophic cardiomyopathy (HCM)	Cardiovascular disease	84	41	0.179	1.88E-02
Endocrine and metabolic disease	Endocrine and metabolic disease	472	294	0.115	7.33E-04
Cushing syndrome	Endocrine and metabolic disease	154	92	0.166	1.54E-03
Type I diabetes mellitus	Endocrine and metabolic disease	40	31	0.302	2.85E-03
Insulin resistance	Endocrine and metabolic disease	103	80	0.181	7.03E-03
AGE-RAGE signaling pathway in diabetic complications	Endocrine and metabolic disease	94	75	0.173	1.50E-02
Type II diabetes mellitus	Endocrine and metabolic disease	45	22	0.234	2.86E-02
Infectious disease: bacterial	Infectious disease: bacterial	446	211	0.123	7.33E-04
Bacterial invasion of epithelial cells	Infectious disease: bacterial	72	40	0.274	7.33E-04
Staphylococcus aureus infection	Infectious disease: bacterial	54	29	0.293	7.33E-04
Shigellosis	Infectious disease: bacterial	61	32	0.259	1.89E-03
Epithelial cell signaling in Helicobacter pylori infection	Infectious disease: bacterial	67	39	0.211	1.11E-02
Human T-cell leukemia virus 1 infection	Infectious disease: viral	209	146	0.200	7.33E-04
Human cytomegalovirus infection	Infectious disease: viral	217	142	0.146	1.19E-03
Human papillomavirus infection	Infectious disease: viral	321	252	0.110	2.56E-03
Infectious disease: viral	Infectious disease: viral	1156	916	0.054	7.45E-03
HumanDiseases	HumanDiseases	2783	1640	0.085	7.33E-04
ADME	ADME	279	167	0.090	4.93E-02

Table S7 | Variants with PBS values <-0.5.

Chr	rsID	Allele	Biotype	Symbol	Mutation		PBS	F_{ST}			Allele frequency		
					Codon	Amino acid		XJU-EAS	XJU-EUR	EAS-EUR	XJU	EAS	EUR
2	rs4149433	T	intron	SULT1C4	-	-	-0.795	0.515	0.397	0.940	0.391	0.942	0.000
2	rs11123695	T	intron	GCC2	-	-	-0.683	0.488	0.392	0.921	0.386	0.922	0.000
2	rs59407435	T	intron	LIMS1	-	-	-0.683	0.488	0.392	0.921	0.386	0.922	0.000
2	rs1866188	A	intron	LIMS1	-	-	-0.708	0.496	0.392	0.925	0.386	0.927	0.000
2	rs12614691	A	intron	RANBP2	-	-	-0.687	0.469	0.408	0.921	0.402	0.922	0.000
2	rs72627476	G	intron	CCDC138	-	-	-0.714	0.460	0.425	0.925	0.418	0.927	0.000
2	rs4676213	T	intron	CCDC138	-	-	-0.714	0.460	0.425	0.925	0.418	0.927	0.000
2	rs3827760	G	missense	EDAR	c.1109T>C	p.Val370Ala	-0.770	0.477	0.425	0.935	0.418	0.937	0.000
2	rs260643	G	intron	EDAR	-	-	-0.549	0.454	0.341	0.880	0.511	0.034	0.919
2	rs922452	T	intron	EDAR	-	-	-0.965	0.538	0.408	0.960	0.402	0.961	0.000
2	rs260712	G	intron	EDAR	-	-	-0.521	0.519	0.268	0.876	0.446	0.976	0.096
2	rs17034666	A	intron	EDAR	-	-	-0.668	0.535	0.323	0.917	0.413	0.966	0.045
2	rs260707	T	intron	EDAR	-	-	-0.610	0.509	0.320	0.902	0.446	0.971	0.066
2	rs388139	A	intron	EDAR	-	-	-0.610	0.509	0.320	0.902	0.446	0.971	0.066
2	rs260708	T	intron	EDAR	-	-	-0.610	0.509	0.320	0.902	0.446	0.971	0.066
2	rs365060	G	intron	EDAR	-	-	-0.609	0.519	0.311	0.902	0.446	0.976	0.071
2	rs260685	A	intron	EDAR	-	-	-0.612	0.504	0.326	0.902	0.451	0.971	0.066
2	rs154998	T	intron	EDAR	-	-	-0.613	0.498	0.332	0.902	0.457	0.971	0.066
2	rs260686	C	intron	EDAR	-	-	-0.613	0.498	0.332	0.902	0.457	0.971	0.066
2	rs260687	T	intron	EDAR	-	-	-0.613	0.498	0.332	0.902	0.543	0.029	0.934
2	rs260688	G	intron	EDAR	-	-	-0.615	0.486	0.344	0.902	0.533	0.029	0.934
2	rs260689	A	intron	EDAR	-	-	-0.616	0.490	0.341	0.902	0.527	0.024	0.929
2	rs260690	A	intron	EDAR	-	-	-0.613	0.498	0.332	0.902	0.543	0.029	0.934
2	rs260691	T	intron	EDAR	-	-	-0.613	0.498	0.332	0.902	0.543	0.029	0.934
15	rs1426654	G	missense	SLC24A5	c.331A>G	p.Thr111Ala	-1.092	0.544	0.419	0.970	0.413	0.971	0.000
15	rs2413887	C	intron	CTXN2	-	-	-1.092	0.544	0.419	0.970	0.587	0.029	1.000
15	rs2413887	C	intron	SLC12A1	-	-	-1.092	0.544	0.419	0.970	0.587	0.029	1.000
15	rs2413887	C	intron	RP11-605F22.1	-	-	-1.092	0.544	0.419	0.970	0.587	0.029	1.000
15	rs2413887	C	upstream gene	RP11-605F22.2	-	-	-1.092	0.544	0.419	0.970	0.587	0.029	1.000
15	rs2413887	C	intron	SLC12A1	-	-	-1.092	0.544	0.419	0.970	0.587	0.029	1.000

Table S8 | Association between geographic coordinate and allele frequency of facial-morphology as well pigmentation related key variants among XJU individuals.

Chr	rsID	Allele	Biotype	Symbol (reported)	Allele frequency			PBS	Association (location ~ genotype)				Local ancestry	
					XJU	EAS	EUR		Cor. (lat.)	P-value (lat.)	Cor. (long.)	P-value (long.)	EAS	EUR
1	rs4648379	T	intron	PRDM16	0.457	0.549	0.318	-0.028	-	-	-	-	0.533	0.467
1	rs642961	G	intergenic	IRF6	0.783	0.806	0.788	0.000	-	-	-	-	0.429	0.571
2	rs1866188	A	intron	LIMS1	0.386	0.927	0.000	-0.708	0.069	0.031	0.079	0.013	0.424	0.576
2	rs3827760	G	missense	EDAR	0.418	0.937	0.000	-0.770	0.076	0.017	0.086	0.007	0.440	0.560
2	rs7559271	A	intron	PAX3	0.522	0.383	0.611	-0.027	-	-	-	-	0.533	0.467
3	rs61672954	A	intron	-	0.049	0.121	0.000	-0.024	-	-	-	-	0.533	0.467
3	rs17447439	G	intron	TP63	0.043	0.034	0.066	-0.003	-	-	-	-	0.543	0.457
4	rs60159418	A	intron	PCDH7	0.342	0.631	0.040	-0.177	-	-	0.080	0.012	0.505	0.495
4	rs2045323	A	intergenic	DCHS2	0.196	0.184	0.086	0.004	-0.220	0.039	-0.231	0.029	0.609	0.391
5	rs6184	A	missense	GHR	0.092	0.126	0.000	-0.016	-	-	-	-	0.576	0.424
5	rs118078182	A	intron	COL23A1	0.087	0.209	0.005	-0.042	0.075	0.049	0.081	0.034	0.489	0.511
6	rs1852985	T	intron	SUPT3H-RUNX2	0.261	0.238	0.131	0.008	-	-	0.216	0.042	0.522	0.478
6	rs7773292	C	downstream gene	ENPP1	0.495	0.597	0.409	-0.020	-	-	-	-	0.505	0.495
7	rs17640804	C	intron	GLI3	0.098	0.044	0.283	-0.043	-0.133	0.0005	-	-	0.609	0.391
10	rs805722	C	missense	COL17A1	0.821	0.835	0.773	-0.003	-	-	-	-	0.538	0.462
11	rs1868752	G	intergenic	-	0.016	0.039	0.015	-0.001	-	-	-	-	0.543	0.457
20	rs3920540	G	intron	BMP2	0.136	0.117	0.141	0.000	-	-	-	-	0.500	0.500
20	rs927833	T	intron	PAX1	0.098	0.049	0.076	0.006	-	-	-	-	0.370	0.630
2	rs6742078	T	intron	UGT1A	0.293	0.112	0.318	-0.012	-	-	-0.080	0.037	0.516	0.484
5	rs35395	C	intron	SLC45A2	0.342	0.087	0.985	-0.513	-	-	-	-	0.685	0.315
5	rs16891982	G	missense	SLC45A2	0.228	0.015	0.980	-0.874	-	-	-	-	0.685	0.315
6	rs12203592	T	intron	IRF4	0.027	0.000	0.162	-0.026	-	-	-	-	0.516	0.484
6	rs12202284	A	intergenic	IRF4	0.092	0.005	0.212	-0.045	-	-	-	-	0.484	0.516
6	rs6917661	T	3_prime_UTR	OPRM1	0.304	0.388	0.232	-0.017	-	-	-	-	0.500	0.500
7	rs2110015	T	intergenic	SNX13	0.658	0.816	0.520	-0.050	-	-	-	-	0.473	0.527
7	rs12668421	T	intron	EGFR	0.500	0.859	0.278	-0.159	0.077	0.021	-	-	0.457	0.543
9	rs872257	A	intron	SMARCA2/VLDLR	0.614	0.553	0.717	-0.016	-0.164	0.017	-	-	0.554	0.446
9	rs13289810	G	intergenic	TYRP1	0.217	0.039	0.374	-0.075	-	-	-0.069	0.039	0.533	0.467
9	rs10756819	A	intron	BNC2	0.614	0.617	0.652	0.000	-	-	-	-	0.505	0.495
9	rs12350739	A	intergenic	BNC2	0.212	0.000	0.581	-0.176	-	-	-	-	0.500	0.500
10	rs376397	G	intron	GATA3	0.701	0.709	0.687	0.000	-	-	-	-	0.489	0.511
10	rs6602666	G	intergenic	BEND7/PRPF18	0.011	0.010	0.000	0.001	-	-	-	-	0.527	0.473
11	rs11230664	C	intron	DDB1/TMEM138	0.043	0.029	0.005	0.007	-	-	-	-	0.609	0.391
11	rs2513329	C	synonymous	DDB1/TMEM138	0.957	0.971	0.995	0.007	-	-	-	-	0.609	0.391
11	rs7948623	A	downstream gene	DDB1/TMEM138	0.984	1.000	1.000	-	-	-	-	-	0.614	0.386
11	rs10831496	G	intron	GMR5	0.598	0.752	0.338	-0.082	-	-	-	-	0.565	0.435
11	rs1042602	A	missense	TYR	0.152	0.000	0.399	-0.099	-	-	-	-	0.582	0.418
12	rs642742	T	intergenic	KITLG	0.212	0.301	0.182	-0.009	-	-	-	-	0.543	0.457
15	rs1800414	C	missense	OCA2	0.147	0.592	0.000	-0.150	-	-	0.189	0.005	0.571	0.429
15	rs74653330	T	missense	OCA2	0.076	0.019	0.005	0.045	-	-	-	-	0.571	0.429
15	rs1800404	T	synonymous	OCA2	0.592	0.393	0.823	-0.094	-	-	-	-	0.576	0.424
15	rs7495174	G	intron	OCA2	0.353	0.646	0.061	-0.174	-	-	-	-	0.630	0.370
15	rs6497271	G	intron	HERC2	0.967	0.971	0.985	0.001	-	-	-	-	0.636	0.364
15	rs12913832	G	intron	HERC2	0.147	0.000	0.768	-0.248	-	-	-	-	0.636	0.364
15	rs4932620	C	intron	HERC2	0.973	0.981	0.990	0.001	-	-	-	-	0.614	0.386
15	rs4424881	T	intron	APBA2	0.408	0.612	0.136	-0.112	0.134	0.049	0.157	0.021	0.587	0.413
15	rs1426654	G	missense	SLC24A5	0.413	0.971	0.000	-1.092	0.100	0.003	0.091	0.006	0.440	0.560
16	rs1805007	T	missense	MC1R	0.005	0.005	0.126	-0.004	-	-	-	-	0.522	0.478

Chr	rsID	Allele	Biotype	Symbol (reported)	Allele frequency			PBS	Association (location ~ genotype)				Local ancestry	
					XJU	EAS	EUR		Cor. (lat.)	P-value (lat.)	Cor. (long.)	P-value (long.)	EAS	EUR
16	rs4268748	C	intron	MC1R	0.196	0.228	0.313	0.009	-	-	-	-	0.522	0.478
19	rs56203814	T	synonymous	MFSD12	0.005	0.000	0.005	0.000	-	-	-	-	0.473	0.527
19	rs10424065	T	intron	MFSD12	0.011	0.000	0.005	0.003	-	-	-	-	0.473	0.527
19	rs2240751	G	missense	MFSD12	0.190	0.398	0.005	-0.092	-	-	-	-	0.478	0.522
19	rs6510760	A	intron	MFSD12	0.168	0.078	0.051	0.050	-	-	-	-	0.484	0.516
19	rs112332856	C	intron	MFSD12	0.087	0.000	0.020	0.057	-	-	-	-	0.484	0.516
20	rs6059655	G	intron	ASIP	0.989	1.000	0.914	-0.013	-	-	-	-	0.424	0.576
20	rs4911414	G	intergenic	ASIP	0.821	0.869	0.672	-0.025	-	-	-0.082	0.032	0.424	0.576
20	rs6058017	G	3_prime_UTR	ASIP	0.168	0.243	0.121	-0.014	-	-	-	-	0.413	0.587

Note: Variants were reported to be associated with the facial morphology of XJU (89, 90) and skin pigmentation across the world-wide populations (52, 74, 91-112). The correlation between the individual genotypes and geographic coordinates were estimated based on the imputed data, and only correlations passed the threshold (*P* <0.05) were provided.

Table S9 | Enrichment of the ancestry-biased components between reconstructed ancestral population and reference population.

ID	Description	GeneRatio	BgRatio	p.adjust
**aEAS vs. EAS, GO enrichment**				
GO:0016339	calcium-dependent cell-cell adhesion via plasma membrane cell adhesion molecules	7/224	48/18493	0.0054
**aEUR vs. EUR, GO enrichment**				
GO:0050911	detection of chemical stimulus involved in sensory perception of smell	41/214	427/18493	1.58E-22
GO:0007608	sensory perception of smell	41/214	453/18493	7.84E-22
GO:0050907	detection of chemical stimulus involved in sensory perception	41/214	477/18493	3.82E-21
**aEUR vs. EUR, KEGG enrichment**				
hsa04740	Olfactory transduction	41/117	448/8033	1.44E-20

Note: Enrichment analysis was conducted using "clusterProfiler" (113). Only pathways and GO categories passed the threshold (BH-corrected P <0.05) were presented. The column "GeneRatio" denotes (# differentiated genes in pathway / # differentiated genes), and the column "BgRatio" denotes (# genes in pathway / # all the background genes).

Table S10 | Enrichment of the ancestry-biased components.

ID	Description	GeneRatio	BgRatio	p.adjust
**EAS-biased, GO enrichment**				
GO:0002476	antigen processing and presentation of endogenous peptide antigen via MHC class Ib	6/172	8/18493	2.21E-08
GO:0002483	antigen processing and presentation of endogenous peptide antigen	7/172	14/18493	2.21E-08
GO:0002428	antigen processing and presentation of peptide antigen via MHC class Ib	6/172	9/18493	4.15E-08
GO:0019883	antigen processing and presentation of endogenous antigen	7/172	21/18493	3.55E-07
GO:0002479	antigen processing and presentation of exogenous peptide antigen via MHC class I, TAP-dependent	7/172	26/18493	1.54E-06
GO:0002475	antigen processing and presentation via MHC class Ib	6/172	16/18493	1.87E-06
GO:0002480	antigen processing and presentation of exogenous peptide antigen via MHC class I, TAP-independent	5/172	9/18493	2.89E-06
GO:0042590	antigen processing and presentation of exogenous peptide antigen via MHC class I	7/172	30/18493	2.89E-06
GO:0002478	antigen processing and presentation of exogenous peptide antigen	11/172	126/18493	7.49E-06
GO:0001912	positive regulation of leukocyte mediated cytotoxicity	8/172	55/18493	1.02E-05
GO:0019884	antigen processing and presentation of exogenous antigen	11/172	134/18493	1.16E-05
GO:0048002	antigen processing and presentation of peptide antigen	11/172	139/18493	1.56E-05
GO:0019882	antigen processing and presentation	12/172	175/18493	1.77E-05
GO:0031343	positive regulation of cell killing	8/172	64/18493	2.45E-05
GO:0002449	lymphocyte mediated immunity	16/172	344/18493	2.45E-05
GO:0060333	interferon-gamma-mediated signaling pathway	9/172	90/18493	2.50E-05
GO:0002474	antigen processing and presentation of peptide antigen via MHC class I	7/172	46/18493	3.01E-05
GO:0002460	adaptive immune response based on somatic recombination of immune receptors built from immunoglobulin superfamily domains	16/172	354/18493	3.01E-05
GO:0042742	defense response to bacterium	15/172	319/18493	4.20E-05
GO:0001913	T cell mediated cytotoxicity	7/172	50/18493	4.86E-05
GO:0001910	regulation of leukocyte mediated cytotoxicity	8/172	76/18493	6.38E-05
GO:0031341	regulation of cell killing	8/172	88/18493	1.89E-04
GO:0001906	cell killing	10/172	158/18493	2.53E-04
GO:0001916	positive regulation of T cell mediated cytotoxicity	5/172	26/18493	3.93E-04
GO:0002456	T cell mediated immunity	8/172	102/18493	5.08E-04
GO:0001909	leukocyte mediated cytotoxicity	8/172	105/18493	5.84E-04
GO:0002708	positive regulation of lymphocyte mediated immunity	8/172	105/18493	5.84E-04
GO:0071346	cellular response to interferon-gamma	10/172	178/18493	6.04E-04
GO:0002706	regulation of lymphocyte mediated immunity	9/172	146/18493	8.09E-04
GO:0001914	regulation of T cell mediated cytotoxicity	5/172	33/18493	1.08E-03
GO:0034341	response to interferon-gamma	10/172	198/18493	1.39E-03
GO:0007608	sensory perception of smell	15/172	453/18493	1.81E-03
GO:0002705	positive regulation of leukocyte mediated immunity	8/172	131/18493	2.39E-03
GO:0050851	antigen receptor-mediated signaling pathway	11/172	259/18493	2.39E-03
GO:0002709	regulation of T cell mediated immunity	6/172	66/18493	2.48E-03
GO:0050911	detection of chemical stimulus involved in sensory perception of smell	14/172	427/18493	3.38E-03
GO:0002711	positive regulation of T cell mediated immunity	5/172	46/18493	4.57E-03
GO:0046967	cytosol to ER transport	2/172	2/18493	5.79E-03
GO:0002703	regulation of leukocyte mediated immunity	9/172	196/18493	6.12E-03
GO:0006910	phagocytosis, recognition	6/172	84/18493	8.46E-03
GO:0050907	detection of chemical stimulus involved in sensory perception	14/172	477/18493	9.58E-03
GO:0060337	type I interferon signaling pathway	6/172	89/18493	1.08E-02
GO:0071357	cellular response to type I interferon	6/172	89/18493	1.08E-02
GO:0034340	response to type I interferon	6/172	94/18493	1.43E-02
GO:0019748	secondary metabolic process	5/172	62/18493	1.54E-02
GO:0002768	immune response-regulating cell surface receptor signaling pathway	13/172	445/18493	1.54E-02
GO:0002824	positive regulation of adaptive immune response based on somatic recombination of immune receptors built from immunoglobulin superfamily domains	6/172	100/18493	1.87E-02
GO:0032094	response to food	4/172	37/18493	2.00E-02
GO:0002822	regulation of adaptive immune response based on somatic recombination of immune receptors built from immunoglobulin superfamily domains	7/172	144/18493	2.10E-02
GO:0002821	positive regulation of adaptive immune response	6/172	105/18493	2.28E-02
GO:0051249	regulation of lymphocyte activation	13/172	472/18493	2.37E-02
GO:0002455	humoral immune response mediated by circulating immunoglobulin	7/172	149/18493	2.37E-02
GO:0009298	GDP-mannose biosynthetic process	2/172	4/18493	2.37E-02
GO:0018894	dibenzo-p-dioxin metabolic process	2/172	4/18493	2.37E-02
GO:0002429	immune response-activating cell surface receptor signaling pathway	12/172	414/18493	2.37E-02
GO:0006911	phagocytosis, engulfment	6/172	112/18493	2.87E-02
GO:0002819	regulation of adaptive immune response	7/172	157/18493	3.03E-02
GO:0002699	positive regulation of immune effector process	8/172	209/18493	3.40E-02
GO:0099024	plasma membrane invagination	6/172	121/18493	4.09E-02
GO:0050853	B cell receptor signaling pathway	6/172	122/18493	4.20E-02
GO:0022408	negative regulation of cell-cell adhesion	7/172	170/18493	4.51E-02
GO:0051251	positive regulation of lymphocyte activation	10/172	333/18493	4.72E-02
GO:0031629	synaptic vesicle fusion to presynaptic active zone membrane	3/172	23/18493	4.96E-02
**EAS-biased, KEGG enrichment**				
hsa04612	Antigen processing and presentation	10/82	78/8041	6.67E-07
hsa05330	Allograft rejection	7/82	38/8041	5.80E-06

ID	Description	GeneRatio	BgRatio	p.adjust
hsa05332	Graft-versus-host disease	7/82	41/8041	6.72E-06
hsa04940	Type I diabetes mellitus	7/82	43/8041	7.10E-06
hsa05320	Autoimmune thyroid disease	7/82	53/8041	2.50E-05
hsa05416	Viral myocarditis	7/82	60/8041	4.94E-05
hsa04514	Cell adhesion molecules (CAMs)	9/82	148/8041	3.40E-04
hsa04145	Phagosome	9/82	152/8041	3.68E-04
hsa05169	Epstein-Barr virus infection	10/82	201/8041	5.06E-04
hsa05166	Human T-cell leukemia virus 1 infection	10/82	219/8041	9.44E-04
hsa05170	Human immunodeficiency virus 1 infection	9/82	212/8041	3.50E-03
hsa04740	Olfactory transduction	13/82	448/8041	6.12E-03
hsa04218	Cellular senescence	7/82	160/8041	1.21E-02
hsa04650	Natural killer cell mediated cytotoxicity	6/82	131/8041	2.03E-02
hsa05150	Staphylococcus aureus infection	5/82	96/8041	2.55E-02
hsa05167	Kaposi sarcoma-associated herpesvirus infection	7/82	189/8041	2.55E-02
hsa05310	Asthma	3/82	31/8041	2.95E-02
hsa05203	Viral carcinogenesis	7/82	204/8041	3.46E-02
hsa05340	Primary immunodeficiency	3/82	38/8041	4.70E-02
**EUR-biased, KEGG enrichment**				
hsa04650	Natural killer cell mediated cytotoxicity	7/68	129/8027	0.0130
hsa04612	Antigen processing and presentation	5/68	73/8027	0.0162
hsa05332	Graft-versus-host disease	4/68	41/8027	0.0162
**EAS/SIB-biased, KEGG enrichment**				
hsa04740	Olfactory transduction	14/81	448/8048	0.0208
**EUR/SAS-biased, GO enrichment**				
GO:0002476	antigen processing and presentation of endogenous peptide antigen via MHC class Ib	3/143	8/18493	0.0225
GO:0002428	antigen processing and presentation of peptide antigen via MHC class Ib	3/143	9/18493	0.0225
GO:0002480	antigen processing and presentation of exogenous peptide antigen via MHC class I, TAP-independent	3/143	9/18493	0.0225
GO:0001913	T cell mediated cytotoxicity	5/143	50/18493	0.0225
GO:0006438	valyl-tRNA aminoacylation	2/143	2/18493	0.0238
GO:0001912	positive regulation of leukocyte mediated cytotoxicity	5/143	55/18493	0.0238
GO:0031343	positive regulation of cell killing	5/143	64/18493	0.0400
GO:0002483	antigen processing and presentation of endogenous peptide antigen	3/143	14/18493	0.0400
GO:0002708	positive regulation of lymphocyte mediated immunity	6/143	105/18493	0.0400

Note: Enrichment analysis was conducted using "clusterProfiler" (113). Only pathways and GO categories passed the threshold (BH-corrected P <0.05) were presented. The column "GeneRatio" denotes (# ancestry-biased genes in the given pathway / # ancestry-biased genes), and the column "BgRatio" denotes (# genes in the given pathway / # all the background genes).

References

1. Li, H, Durbin, R. Fast and accurate long-read alignment with Burrows-Wheeler transform. *Bioinformatics*. 2010; **26**(5): 589-95.
2. Li, H, Handsaker, B, Wysoker, A, *et al.* The Sequence Alignment/Map format and SAMtools. *Bioinformatics*. 2009; **25**(16): 2078-9.
3. DePristo, MA, Banks, E, Poplin, R, *et al.* A framework for variation discovery and genotyping using next-generation DNA sequencing data. *Nat Genet*. 2011; **43**(5): 491-8.
4. McKenna, A, Hanna, M, Banks, E, *et al.* The Genome Analysis Toolkit: a MapReduce framework for analyzing next-generation DNA sequencing data. *Genome research*. 2010; **20**(9): 1297-303.
5. Lu, DS, Lou, HY, Yuan, K, *et al.* Ancestral Origins and Genetic History of Tibetan Highlanders. *Am J Hum Genet*. 2016; **99**(3): 580-94.
6. Zhang, C, Lu, Y, Feng, QD, *et al.* Differentiated demographic histories and local adaptations between Sherpas and Tibetans. *Genome Biology*. 2017; **18**: 18.
7. Mallick, S, Li, H, Lipson, M, *et al.* The Simons Genome Diversity Project: 300 genomes from 142 diverse populations. *Nature*. 2016; **538**(7624): 201-+.
8. Karolchik, D, Hinrichs, AS, Furey, TS, *et al.* The UCSC Table Browser data retrieval tool. *Nucleic acids research*. 2004; **32**(Database issue): D493-6.
9. Dobin, A, Davis, CA, Schlesinger, F, *et al.* STAR: ultrafast universal RNA-seq aligner. *Bioinformatics*. 2013; **29**(1): 15-21.
10. Li, B, Dewey, CN. RSEM: accurate transcript quantification from RNA-Seq data with or without a reference genome. *BMC Bioinformatics*. 2011; **12**: 16.
11. McLaren, W, Gil, L, Hunt, SE, *et al.* The Ensembl Variant Effect Predictor. *Genome Biology*. 2016; **17**: 14.
12. Kircher, M, Witten, DM, Jain, P, *et al.* A general framework for estimating the relative pathogenicity of human genetic variants. *Nature Genet*. 2014; **46**(3): 310-+.
13. Cooper, GM, Stone, EA, Asimenos, G, *et al.* Distribution and intensity of constraint in mammalian genomic sequence. *Genome Res*. 2005; **15**(7): 901-13.
14. Chang, CC, Chow, CC, Tellier, L, *et al.* Second-generation PLINK: rising to the challenge of larger and richer datasets. *GigaScience*. 2015; **4**: 16.
15. Feng, QD, Lu, Y, Ni, XM, *et al.* Genetic history of Xinjiang's Uyghurs suggests Bronze Age multiple-way contacts in Eurasia. *Mol Biol Evol*. 2017; **34**(10): 2572-82.
16. Delaneau, O, Marchini, J, Zagury, JF. A linear complexity phasing method for thousands of genomes. *Nat Methods*. 2012; **9**(2): 179-81.
17. Howie, BN, Donnelly, P, Marchini, J. A Flexible and Accurate Genotype Imputation Method for the Next Generation of Genome-Wide Association Studies. *PLoS Genet*. 2009; **5**(6): 15.
18. Lazaridis, I, Patterson, N, Mittnik, A, *et al.* Ancient human genomes suggest three ancestral populations for present-day Europeans. *Nature*. 2014; **513**(7518): 409-+.
19. Altshuler, DM, Durbin, RM, Abecasis, GR, *et al.* A global reference for human genetic variation. *Nature*. 2015; **526**(7571): 68-+.
20. Alexander, DH, Novembre, J, Lange, K. Fast model-based estimation of ancestry in unrelated individuals. *Genome Res*. 2009; **19**(9): 1655-64.
21. Pagani, L, Lawson, DJ, Jagoda, E, *et al.* Genomic analyses inform on migration events during the peopling of Eurasia. *Nature*. 2016; **538**(7624): 238-+.
22. Gao, Y, Zhang, C, Yuan, LY, *et al.* PGG.Han: the Han Chinese genome database and analysis platform. *Nucleic Acids Res*. 2020; **48**(D1): D971-D6.
23. Zhang, C, Gao, Y, Ning, Z, *et al.* PGG.SNV: understanding the evolutionary and medical implications of human single nucleotide variations in diverse populations. *Genome biology*. 2019; **20**(1): 215.
24. Schuenemann, VJ, Peltzer, A, van Pelt, B, *et al.* Ancient Egyptian mummy genomes suggest an increase of Sub-Saharan African ancestry in post-Roman periods. *Nat Commun*. 2017; **8**: 11.
25. Olalde, I, Brace, S, Allentoft, ME, *et al.* The Beaker phenomenon and the genomic transformation of northwest Europe. *Nature*. 2018; **555**(7695): 190-+.
26. Mathieson, I, Lazaridis, I, Rohland, N, *et al.* Genome-wide patterns of selection in 230 ancient Eurasians. *Nature*. 2015; **528**(7583): 499-+.
27. Mathieson, I, Alpaslan-Roodenberg, S, Posth, C, *et al.* The genomic history of southeastern Europe. *Nature*. 2018; **555**(7695): 197-+.

28. Haak, W, Lazaridis, I, Patterson, N*, et al.* Massive migration from the steppe was a source for Indo-European languages in Europe. *Nature*. 2015; **522**(7555): 207-+.
29. Lipson, M, Szecsenyi-Nagy, A, Mallick, S*, et al.* Parallel palaeogenomic transects reveal complex genetic history of early European farmers. *Nature*. 2017; **551**(7680): 368-+.
30. Allentoft, ME, Sikora, M, Sjogren, KG*, et al.* Population genomics of Bronze Age Eurasia. *Nature*. 2015; **522**(7555): 167-+.
31. Yang, MA, Fan, X, Sun, B*, et al.* Ancient DNA indicates human population shifts and admixture in northern and southern China. *Science (New York, NY)*. 2020; **369**(6501): 282-8.
32. Freedman, ML, Haiman, CA, Patterson, N*, et al.* Admixture mapping identifies 8q24 as a prostate cancer risk locus in African-American men. *Proc Natl Acad Sci U S A*. 2006; **103**(38): 14068-73.
33. Lawson, DJ, Hellenthal, G, Myers, S*, et al.* Inference of population structure using dense haplotype data. *PLoS Genet*. 2012; **8**(1): 16.
34. Xu, S, Jin, W, Jin, L. Haplotype-Sharing Analysis Showing Uyghurs Are Unlikely Genetic Donors. *Mol Biol Evol*. 2009; **26**(10): 2197-206.
35. Yu, GC, Smith, DK, Zhu, HC*, et al.* GGTREE: an R package for visualization and annotation of phylogenetic trees with their covariates and other associated data. *Methods Ecol Evol*. 2017; **8**(1): 28-36.
36. Weir, BS, Cockerham, CC. Estimating F-statistics for the analysis of population-structure. *Evolution*. 1984; **38**(6): 1358-70.
37. Keinan, A, Mullikin, JC, Patterson, N*, et al.* Accelerated genetic drift on chromosome X during the human dispersal out of Africa. *Nature Genet*. 2009; **41**(1): 66-70.
38. Buniello, A, MacArthur, JAL, Cerezo, M*, et al.* The NHGRI-EBI GWAS Catalog of published genome-wide association studies, targeted arrays and summary statistics 2019. *Nucleic Acids Research*. 2019; **47**(D1): D1005-D12.
39. Durinck, S, Spellman, PT, Birney, E*, et al.* Mapping identifiers for the integration of genomic datasets with the R/Bioconductor package biomaRt. *Nat Protoc*. 2009; **4**(8): 1184-91.
40. Dias-Alves, T, Mairal, J, Blum, MGB. Loter: A Software Package to Infer Local Ancestry for a Wide Range of Species. *Mol Biol Evol*. 2018; **35**(9): 2318-26.
41. Tajima, F. Statistical-method for testing the neutral mutation hypothesis by DNA polymorphism. *Genetics*. 1989; **123**(3): 585-95.
42. Nei, M, Li, WH. Mathematical-model for studying genetic-variation in terms of restriction endonucleases. *Proc Natl Acad Sci U S A*. 1979; **76**(10): 5269-73.
43. Nei, M, Tajima, F. DNA polymorphism detectable by restriction endonucleases. *Genetics*. 1981; **97**(1): 145-63.
44. Danecek, P, Auton, A, Abecasis, G*, et al.* The variant call format and VCFtools. *Bioinformatics*. 2011; **27**(15): 2156-8.
45. McEvoy, BP, Powell, JE, Goddard, ME*, et al.* Human population dispersal "Out of Africa" estimated from linkage disequilibrium and allele frequencies of SNPs. *Genome Res*. 2011; **21**(6): 821-9.
46. Fu, WQ, Gittelman, RM, Bamshad, MJ*, et al.* Characteristics of neutral and deleterious protein-coding variation among individuals and populations. *Am J Hum Genet*. 2014; **95**(4): 421-36.
47. Kanehisa, M, Furumichi, M, Tanabe, M*, et al.* KEGG: new perspectives on genomes, pathways, diseases and drugs. *Nucleic Acids Research*. 2017; **45**(D1): D353-D61.
48. Clark, NR, Ma'ayan, A. Introduction to Statistical Methods for Analyzing Large Data Sets: Gene-Set Enrichment Analysis. *Sci Signal*. 2011; **4**(190): 5.
49. Voight, BF, Kudaravalli, S, Wen, XQ*, et al.* A map of recent positive selection in the human genome. *PLoS Biol*. 2006; **4**(3): 446-58.
50. Lonsdale, J, Thomas, J, Salvatore, M*, et al.* The Genotype-Tissue Expression (GTEx) project. *Nature Genetics*. 2013; **45**(6): 580-5.
51. Wu, SJ, Zhang, MF, Yang, XZ*, et al.* Genome-wide association studies and CRISPR/Cas9-mediated gene editing identify regulatory variants influencing eyebrow thickness in humans. *PLoS Genet*. 2018; **14**(9): 22.
52. Lamason, RL, Mohideen, M, Mest, JR*, et al.* SLC24A5, a putative cation exchanger, affects pigmentation in zebrafish and humans. *Science*. 2005; **310**(5755): 1782-6.
53. Soejima, M, Koda, Y. Population differences of two coding SNPs in pigmentation-related genes SLC24A5 and SLC45A2. *Int J Legal Med*. 2007; **121**(1): 36-9.
54. Mallick, CB, Iliescu, FM, Mols, M*, et al.* The light skin allele of SLC24A5 in South Asians and Europeans shares identity by descent. *PLoS Genet*. 2013; **9**(11): 13.

55. Rishishwar, L, Conley, AB, Wigington, CH, *et al.* Ancestry, admixture and fitness in Colombian genomes. *Sci Rep*. 2015; **5**: 16.
56. Garud, NR, Messer, PW, Buzbas, EO, *et al.* Recent Selective Sweeps in North American Drosophila melanogaster Show Signatures of Soft Sweeps. *PLoS Genet*. 2015; **11**(2): 32.
57. Harris, AM, Garud, NR, DeGiorgio, M. Detection and Classification of Hard and Soft Sweeps from Unphased Genotypes by Multilocus Genotype Identity. *Genetics*. 2018; **210**(4): 1429-52.
58. Kothapalli, KSD, Ye, K, Gadgil, MS, *et al.* Positive Selection on a Regulatory Insertion-Deletion Polymorphism in FADS2 Influences Apparent Endogenous Synthesis of Arachidonic Acid. *Mol Biol Evol*. 2016; **33**(7): 1726-39.
59. Shaffer, JR, Li, JX, Lee, MK, *et al.* Multiethnic GWAS Reveals Polygenic Architecture of Earlobe Attachment. *Am J Hum Genet*. 2017; **101**(6): 913-24.
60. Adhikari, K, Reales, G, Smith, AJP, *et al.* A genome-wide association study identifies multiple loci for variation in human ear morphology. *Nat Commun*. 2015; **6**.
61. Sabeti, PC, Varilly, P, Fry, B, *et al.* Genome-wide detection and characterization of positive selection in human populations. *Nature*. 2007; **449**(7164): 913-U12.
62. Yi, X, Liang, Y, Huerta-Sanchez, E, *et al.* Sequencing of 50 human exomes reveals adaptation to high altitude. *Science*. 2010; **329**(5987): 75-8.
63. Fujimoto, A, Ohashi, J, Nishida, N, *et al.* A replication study confirmed the EDAR gene to be a major contributor to population differentiation regarding head hair thickness in Asia. *Hum Genet*. 2008; **124**(2): 179-85.
64. Tan, JZ, Yang, YJ, Tang, K, *et al.* The adaptive variant EDARV370A is associated with straight hair in East Asians. *Hum Genet*. 2013; **132**(10): 1187-91.
65. Peng, QQ, Li, JX, Tan, JZ, *et al.* EDARV370A associated facial characteristics in Uyghur population revealing further pleiotropic effects. *Hum Genet*. 2016; **135**(1): 99-108.
66. Kimura, R, Yamaguchi, T, Takeda, M, *et al.* A Common Variation in EDAR Is a Genetic Determinant of Shovel-Shaped Incisors. *Am J Hum Genet*. 2009; **85**(4): 528-35.
67. Park, JH, Yamaguchi, T, Watanabe, C, *et al.* Effects of an Asian-specific nonsynonymous EDAR variant on multiple dental traits. *J Hum Genet*. 2012; **57**(8): 508-14.
68. Tan, JZ, Peng, QQ, Li, JX, *et al.* Characteristics of dental morphology in the Xinjiang Uyghurs and correlation with the EDARV370A variant. *Sci China-Life Sci*. 2014; **57**(5): 510-8.
69. Chang, SH, Jobling, S, Brennan, K, *et al.* Enhanced EDAR signalling has pleiotropic effects on Craniofacial and Cutaneous Glands. *PLoS One*. 2009; **4**(10): 8.
70. Kamberov, YG, Wang, SJ, Tan, JZ, *et al.* Modeling recent human evolution in mice by expression of a selected EDAR variant. *Cell*. 2013; **152**(4): 691-702.
71. Yuasa, I, Umetsu, K, Harihara, S, *et al.* Distribution of the F374 allele of the SLC45A2 (MATP) gene and founder-haplotype analysis. *Ann Hum Genet*. 2006; **70**: 802-11.
72. Stokowski, RP, Pant, PVK, Dadd, T, *et al.* A genomewide association study of skin pigmentation in a South Asian population. *Am J Hum Genet*. 2007; **81**(6): 1119-32.
73. Izagirre, N, Garcia, I, Junquera, C, *et al.* A scan for signatures of positive selection in candidate loci for skin pigmentation in humans. *Mol Biol Evol*. 2006; **23**(9): 1697-706.
74. Graf, J, Hodgson, R, van Daal, A. Single nucleotide polymorphisms in the MATP gene are associated with normal human pigmentation variation. *Hum Mutat*. 2005; **25**(3): 278-84.
75. Han, Y, Gu, S, Oota, H, *et al.* Evidence of positive selection on a class I ADH locus. *The American Journal of Human Genetics*. 2007; **80**(3): 441-56.
76. Li, H, Mukherjee, N, Soundararajan, U, *et al.* Geographically separate increases in the frequency of the derived ADH1B* 47His allele in eastern and western Asia. *The American Journal of Human Genetics*. 2007; **81**(4): 842-6.
77. Volpe, TA, Kidner, C, Hall, IM, *et al.* Regulation of heterochromatic silencing and histone H3 lysine-9 methylation by RNAi. *Science*. 2002; **297**(5588): 1833-7.
78. van der Harst, P, Verweij, N. Identification of 64 Novel Genetic Loci Provides an Expanded View on the Genetic Architecture of Coronary Artery Disease. *CircRes*. 2018; **122**(3): 433-43.
79. Kichaev, G, Bhatia, G, Loh, P-R, *et al.* Leveraging polygenic functional enrichment to improve GWAS power. *The American Journal of Human Genetics*. 2019; **104**(1): 65-75.
80. Swallow, DM. Genetics of lactase persistence and lactose intolerance. *Annu Rev Genet*. 2003; **37**: 197-219.
81. Tishkoff, SA, Reed, FA, Ranciaro, A, *et al.* Convergent adaptation of human lactase persistence in Africa

and Europe. *Nature Genet*. 2007; **39**(1): 31-40.
82. Morgan, MD, Pairo-Castineira, E, Rawlik, K*, et al.* Genome-wide study of hair colour in UK Biobank explains most of the SNP heritability. *Nat Commun*. 2018; **9**: 10.
83. Ashburner, M, Ball, CA, Blake, JA*, et al.* Gene Ontology: tool for the unification of biology. *Nature Genet*. 2000; **25**(1): 25-9.
84. Hoover, KC, Gokcumen, O, Qureshy, Z*, et al.* Global survey of variation in a human olfactory receptor gene reveals signatures of non-neutral evolution. *Chem Senses*. 2015; **40**(7): 481-8.
85. Liu, JZ, van Sommeren, S, Huang, HL*, et al.* Association analyses identify 38 susceptibility loci for inflammatory bowel disease and highlight shared genetic risk across populations. *Nature Genet*. 2015; **47**(9): 979-+.
86. Langley, SA, Miga, KH, Karpen, GH*, et al.* Haplotypes spanning centromeric regions reveal persistence of large blocks of archaic DNA. *eLife*. 2019; **8**: 15.
87. Gouy, A, Excoffier, L. Polygenic Patterns of Adaptive Introgression in Modern Humans Are Mainly Shaped by Response to Pathogens. *Mol Biol Evol*. 2020; **37**(5): 1420-33.
88. Trimmer, C, Keller, A, Murphy, NR*, et al.* Genetic variation across the human olfactory receptor repertoire alters odor perception. *Proc Natl Acad Sci U S A*. 2019; **116**(19): 9475-80.
89. Adhikari, K, Fuentes-Guajardo, M, Quinto-Sanchez, M*, et al.* A genome-wide association scan implicates DCHS2, RUNX2, GLI3, PAX1 and EDAR in human facial variation. *Nat Commun*. 2016; **7**: 11.
90. Qiao, L, Yang, YJ, Fu, PC*, et al.* Genome-wide variants of Eurasian facial shape differentiation and a prospective model of DNA based face prediction. *J Genet Genomics*. 2018; **45**(8): 419-32.
91. Shriver, MD, Parra, EJ, Dios, S*, et al.* Skin pigmentation, biogeographical ancestry and admixture mapping. *Hum Genet*. 2003; **112**(4): 387-99.
92. Bonilla, C, Boxill, LA, McDonald, SA*, et al.* The 8818G allele of the agouti signaling protein (ASIP) gene is ancestral and is associated with darker skin color in African Americans. *Hum Genet*. 2005; **116**(5): 402-6.
93. Miller, CT, Beleza, S, Pollen, AA*, et al.* cis-regulatory changes in kit ligand expression and parallel evolution of pigmentation in sticklebacks and humans. *Cell*. 2007; **131**(6): 1179-89.
94. Han, JL, Kraft, P, Nan, H*, et al.* A genome-wide association study identifies novel alleles associated with hair color and skin pigmentation. *PLoS Genet*. 2008; **4**(5): 11.
95. Cook, AL, Chen, W, Thurber, AE*, et al.* Analysis of Cultured Human Melanocytes Based on Polymorphisms within the SLC45A2/MATP, SLC24A5/NCKX5, and OCA2/P Loci. *J Invest Dermatol*. 2009; **129**(2): 392-405.
96. Edwards, M, Bigham, A, Tan, JZ*, et al.* Association of the OCA2 Polymorphism His615Arg with Melanin Content in East Asian Populations: Further Evidence of Convergent Evolution of Skin Pigmentation. *PLoS Genet*. 2010; **6**(3): 8.
97. Quillen, EE, Bauchet, M, Bigham, AW*, et al.* OPRM1 and EGFR contribute to skin pigmentation differences between Indigenous Americans and Europeans. *Hum Genet*. 2012; **131**(7): 1073-80.
98. Tsetskhladze, ZR, Canfield, VA, Ang, KC*, et al.* Functional Assessment of Human Coding Mutations Affecting Skin Pigmentation Using Zebrafish. *PLoS One*. 2012; **7**(10): 9.
99. Visser, M, Kayser, M, Palstra, RJ. HERC2 rs12913832 modulates human pigmentation by attenuating chromatin-loop formation between a long-range enhancer and the OCA2 promoter. *Genome Res*. 2012; **22**(3): 446-55.
100. Abe, Y, Tamiya, G, Nakamura, T*, et al.* Association of melanogenesis genes with skin color variation among Japanese females. *J Dermatol Sci*. 2013; **69**(2): 167-72.
101. Beleza, S, Johnson, NA, Candille, SI*, et al.* Genetic Architecture of Skin and Eye Color in an African-European Admixed Population. *PLoS Genet*. 2013; **9**(3): 15.
102. Jacobs, LC, Wollstein, A, Lao, O*, et al.* Comprehensive candidate gene study highlights UGT1A and BNC2 as new genes determining continuous skin color variation in Europeans. *Hum Genet*. 2013; **132**(2): 147-58.
103. Praetorius, C, Grill, C, Stacey, SN*, et al.* A Polymorphism in IRF4 Affects Human Pigmentation through a Tyrosinase-Dependent MITF/TFAP2A Pathway. *Cell*. 2013; **155**(5): 1022-33.
104. Zhang, MF, Song, FJ, Liang, LM*, et al.* Genome-wide association studies identify several new loci associated with pigmentation traits and skin cancer risk in European Americans. *Hum Mol Genet*. 2013; **22**(14): 2948-59.
105. Visser, M, Palstra, RJ, Kayser, M. Human skin color is influenced by an intergenic DNA polymorphism regulating transcription of the nearby BNC2 pigmentation gene. *Hum Mol Genet*. 2014; **23**(21): 5750-62.

106. Liu, F, Visser, M, Duffy, DL*, et al.* Genetics of skin color variation in Europeans: genome-wide association studies with functional follow-up. *Hum Genet*. 2015; **134**(8): 823-35.
107. Crawford, NG, Kelly, DE, Hansen, MEB*, et al.* Loci associated with skin pigmentation identified in African populations. *Science*. 2017; **358**(6365): 887-+.
108. Hernandez-Pacheco, N, Flores, C, Alonso, S*, et al.* Identification of a novel locus associated with skin colour in African-admixed populations. *Sci Rep*. 2017; **7**: 9.
109. Lloyd-Jones, LR, Robinson, MR, Moser, G*, et al.* Inference on the Genetic Basis of Eye and Skin Color in an Admixed Population via Bayesian Linear Mixed Models. *Genetics*. 2017; **206**(2): 1113-26.
110. Martin, AR, Lin, M, Granka, JM*, et al.* An Unexpectedly Complex Architecture for Skin Pigmentation in Africans. *Cell*. 2017; **171**(6): 1340-+.
111. Adhikari, K, Mendoza-Revilla, J, Sohail, A*, et al.* A GWAS in Latin Americans highlights the convergent evolution of lighter skin pigmentation in Eurasia. *Nat Commun*. 2019; **10**: 16.
112. Lona-Durazo, F, Hernandez-Pacheco, N, Fan, SH*, et al.* Meta-analysis of GWA studies provides new insights on the genetic architecture of skin pigmentation in recently admixed populations. *BMC Genet*. 2019; **20**: 16.
113. Yu, GC, Wang, LG, Han, YY*, et al.* clusterProfiler: an R Package for Comparing Biological Themes Among Gene Clusters. *Omics*. 2012; **16**(5): 284-7.
